# Consciously detecting and recognizing a past visual word after its sensory trace is gone

**DOI:** 10.1038/s44271-026-00478-9

**Published:** 2026-06-09

**Authors:** Daphné Rimsky Robert, Matteo Lisi, Kevin Nguy, Roxane Jannin, Thomas Hardy, Nathan Beraud, Claire Sergent

**Affiliations:** 1 Integrative Neuroscience and Cognition Center - UMR 8002 CNRS/Université Paris-Cité, Paris, France; 2 Department of Psychology, Royal Holloway, University of London, London, UK

**Keywords:** Human behaviour, Consciousness, Perception

## Abstract

What is the role of sensory processing in conscious perception? Current theories of consciousness are divided on this question. Some propose that conscious perception arises during the buildup of sensory representations. Others argue for a secondary process that broadcasts these representations to higher-level areas. This second view makes a counter-intuitive prediction: one could consciously perceive abstract representations untied to any low-level sensory feature. We tested this prediction by combining visual masking with retrospective cueing. We found that when visually masked words were followed by a semantically related auditory word, participants were better at detecting this past word and reporting its identity, but were strikingly unable to report its visual features (letter casing or position on screen). This suggests that retro-cueing can help a semantic representation reach awareness even after the associated sensory information has been masked. The mechanisms of conscious access might thus be largely independent of early sensory build-up.

**Figure:**
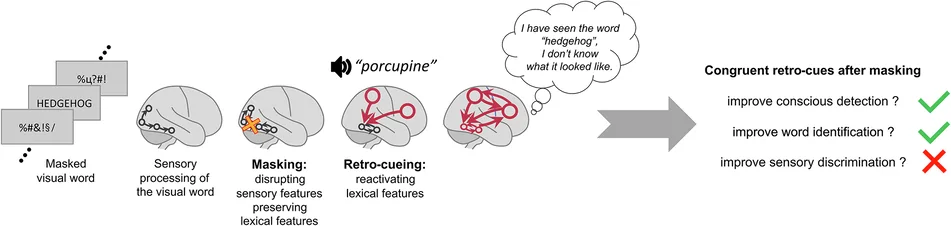

## Abstract (web summary)

This series of studies shows that conscious detection and recognition of a word can be triggered retrospectively, even after its visual features are masked. This suggests that conscious access may be largely independent of early sensory processing.

## Introduction

When does the awareness of an external stimulus emerge during the course of its processing by the brain? This question stands as a significant juncture in ongoing debates about the neural mechanisms of conscious perception^1,2^. Three main processing stages can be distinguished, based on their neurophysiological and computational characteristics^3–6^. First, the onset of a new sensory input, visual, auditory or other, triggers a “feed-forward” sweep of activity, which, via ascending connections, hierarchically mobilizes the different areas in the sensory system, reaching the top levels within approximately 100ms^4,5^. In vision, it goes from the retina to the lateral geniculate nucleus, the primary visual cortex (V1), higher-level visual areas (V2, V3, V4…), and then parietal and temporal areas. At this stage, the encoding of the stimulus is typically quite faithful to the sensory input. But whenever activity reaches a specific level, this also triggers recurrent connections that modulate lower levels in return (e.g., from V2 to V1). This can be identified as a “second stage” which allows the stabilization of sensory representations in a more interpreted form^3,7^. Finally, in a general “third stage”, functional connectivity extends beyond the sensory system, and typically involves frontal, parietal, and cingulate areas, associated with attentional selection, opening a wide repertoire of cognitive functions^5,8,9^.

There is a large consensus in assuming that the first feed-forward sweep is an unconscious stage. Beyond that, the different theories diverge. Theories such as the Local Recurrent Processing^10^ or the Integrated Information Theory^11^ consider that conscious perception arises with the establishment of local recurrent loops within the sensory system^12^, notably because at this stage, stimulus encoding departs from the strict rendering of the input to match the way the stimulus appears to the individual^13–15^. In this view, additional functional connectivity with supra-modal areas (third stage) only has a modulatory effect^16^.

In contrast, other views, such as the Global Neuronal Workspace^9,17^, or Higher-Order theories^18^, consider that this second stage is not sufficient to become conscious of the stimulus, and propose that conscious perception arises if and only if integrated sensory representations are further broadcast to a wider network of supra-modal areas (third stage), allowing a global and flexible functional impact on the cognition of the individual.

So far, experimental work contrasting brain activity for conscious versus non-conscious perception has not been very conclusive: all processing stages have been found to correlate with conscious perception in one study or another^19–22^, including pre-stimulus activity^23,24^. These seemingly contradictory results reflect one limitation of this contrastive method, which reveals *everything* that correlates with the final perception of a stimulus, including events that might favor the future conscious perception of the stimulus, while not being directly part of the mechanisms of conscious perception *per se*.

To move forward, we must identify scenarios where various theories yield contrasted predictions, and subject them to testing. Here, we highlight a strong and rather counter-intuitive prediction of high-level theories such as the Global Neuronal Workspace: if conscious access is largely independent from sensory build-up, then it should be possible to desynchronize conscious access from the timing of the external stimulus^25^. We have started to explore this possibility in several experiments testing whether conscious access to an initially unconscious stimulus could still be triggered by retrospective attention. We have shown that cueing spatial attention towards the former position of a barely visible Gabor patch, at various delays after its disappearance, substantially improved participants’ conscious perception of this stimulus, both on objective and subjective measures. This “retro-perception” effect was still robust 400 ms after the stimulus. It has since been replicated and extended in several studies^26–29^.

Although these studies suggest that it is possible to desynchronize conscious perception from the initial sensory build-up, it might still be argued that, in these experiments, only the first feed-forward sweep of activity was triggered by the stimulus, and that retrospective cueing allowed conscious perception not by connecting sensory representations with higher-level areas, but by allowing local sensory loops to be deployed for the first time.

In the present study, we pushed our approach further by asking the following question: is it possible to induce retrospective conscious access to a past stimulus even after its low-level sensory representations have been degraded by masking? While such a possibility is precluded by theories linking conscious perception with sensory build-up, it is a valid, albeit extreme prediction of theories advocating some independence between the two.

**Fig. 1:**
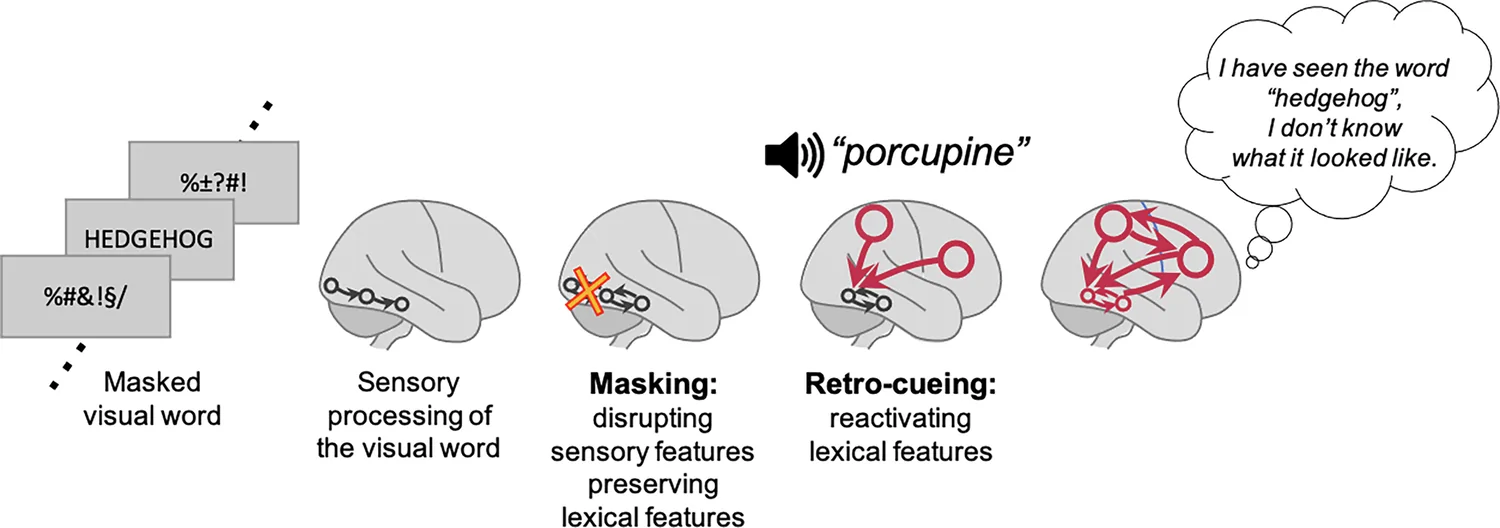
Hypothesis. This figure represents the postulated sequence of brain events associated with our protocol, combining masking and retro-cueing. When a target word is presented in a sequence of masks, it can be processed up to the level of lexical activations (first processing stage). The masks, which share many low-level visual properties with the target, act by stopping recurrent sensory processing and disrupting the low-level sensory representations of the target (masking). This is what prevents conscious perception of the target word in the first place. Still, we hypothesize that a contextually congruent auditory retro-cue may bring the target word to consciousness (retro-cueing), by reactivating the latent lexical representation of the past word and hence giving it a second chance to be broadcast through a fronto-parietal Global Workspace. This would yield conscious access to the word's meaning in the absence of associated low-level information.

We therefore designed an experimental protocol that combines visual word masking with retrospective cueing by an auditory word (Fig. 1). In visual pattern masking, conscious perception of a briefly presented written word can be drastically impaired if the word is immediately preceded and followed by other visual displays with similar low-level properties. Masking is assumed to occur because the masks prevent local recurrent loops to establish a coherent representation of the word^7,30^. in short, masking interferes with the second processing stage. But interestingly, even when the word is rendered totally undetectable by masking, many experiments have established that it can still be processed up to a lexical and/or semantic level, presumably because the feed-forward sweep reaches high-level areas before the masks interfere with low-level representations^31–34^. Hence, after masking, what is left from the visual word is an unconscious lexical and/or semantic trace in temporal areas, while the sensory representations that led to it have been disrupted (Fig. 1).

We know from the masked priming literature that lexical or semantic priming effects disappear for very short durations of the word, suggesting that for these too-brief stimuli, unconscious processing is too weak to reach this level^35^. Classical experiments showing unconscious semantic processing of masked words typically use target durations between 30 and 50 ms, where they find the best compromise allowing semantic processing to occur while conscious perception is still hindered^32,35^. Here we tested a range of four different target durations (12, 24, 36, and 48 ms), spanning both very short durations for which initial processing probably fails to reach semantic levels, and longer durations where unconscious semantic priming is documented^35^.

We tested whether a subsequent semantic cue could retrospectively promote conscious access to the latent semantic representation. The masked visual word was thus followed by an auditory word played over headphones, which could either be semantically related to it (congruent) or not (incongruent). For example, if the masked word was “hedgehog”, the retrospective cue could be either “porcupine” or “automaton” (Fig. 1). Congruent retro-cues might induce, via semantic diffusion^36^, a top-down reactivation of the unconscious trace left by the visual word at the lexical level, promote the sharing of this information to a wider network of areas, and allow conscious access. In principle, this lexical cue could also help retrieve visual traces of the written word, by sharpening expectations about its visual form: for example, the cue “porcupine” leading to associated words such as “hedgehog”, might sharpen visual expectations about a long word with several “h”. This should improve the retrieval of the visual features of the word, such as its case. However, if this cueing occurs after the mask has disrupted sensory features, cueing at this level should fail.

We predicted that following a congruent retro-cue, participants would be better at reporting the identity of the masked word, but unable to report any associated visual details, such as whether the word was written in uppercase or lowercase, or whether it had been presented above or below the fixation point. Crucially, we also predicted that better identification would go hand in hand with improved conscious access, as assessed by improved subjective visibility and improved objective ability to report whether a word has been presented or not (detection sensitivity), both being classical measures of conscious access^37,38^.

We tested these predictions in a series of three experiments involving a total of 51 included participants. We then preregistered and performed two additional sets of experiments to control for a potential alternative interpretation (72 new participants). Overall, the results suggest that retrospective cues can improve conscious access to the high-level representations of a past visual word after its low-level, sensory representations have been degraded by masking. They bring support to the notion that the mechanisms of conscious access may, to a large extent, operate independently from sensory build-up.

## Methods

### Participants

Participant number per experiment was constrained to 18 in order to ensure complete counterbalancing of stimulus presentation across participants. This is similar to the sample size of previous experiments testing other retrospective effects, which, based on a power analysis, included around twenty participants^39^. For Experiment 1, only fifteen participants (average age 23 ± 3.3, 10 females and 5 males) matched our selection criterion (see **participant screening** below), and further recruitment could not be achieved due to external constraints. For Experiment 2, 23 participants were recruited, of which 5 were excluded prior to analysis due to overall performance at the position discrimination task being at chance (<55%) or failure to comply with instructions (average age 27.5 ± 7.1, 9 females and 9 males). For Experiment 3, 26 participants were recruited, of which 8 were excluded prior to analysis due to overall performance at the position discrimination task being at chance (<55%) or failure to comply with instructions (average age 24.5 ± 3.7, 10 females and 8 males). In Experiments 4, 5, 6, and 7, 18 participants were included in each with no exclusion prior to data analysis (average age of the total of 72 participants across studies: 25.4 ± 4.2, 55 females and 17 males). All participants gave informed consent in writing prior to participation, self-selected their preferred gender, and the Université Paris Descartes Review Board CERES approved the protocols for the study in accordance with French regulations and the Declaration of Helsinki. Participants received a compensation of 10€ per hour of their time. Data on socio-economic status, communities of descent, or race/ethnicity were not collected.

### Participant screening

Given the difficulty of the task, participants underwent a screening session before Experiment 1 to exclude participants unable to perform the task, as well as provide training. This session lasted 1 hour and included 140 trials preceded by a short, 18-trial training block with progressively decreasing target duration, from 120 ms to 12 ms. During the screening session, participants were presented with visually masked words (different to the main experiment, matching the same criteria and procedure) but no auditory cue. Participants were asked to identify the word aloud in a microphone and report its case and visibility using the keyboard. The criterion for inclusion in our experiment was that performance at the case discrimination task should, for at least one target duration, be within [65% - 90%] correct responses. In other experiments, we used a simpler screening procedure. The experiment started with a 72-trial-long training session with progressively decreasing target duration as described above. If performance on the case discrimination task over all training trials was below 55% correct responses, we ran a second training session. If performance was again below 55%, they were precluded from participating in the experiment.

### Stimulus-list

Our stimulus list was composed of 432 unique words (targets) from the French language. These words ranged from 3 to 8 letters long ( + one 2-letter word, “os” which translates to “bone”), and did not carry accents of cedillas. They were selected for being strongly associated to another word from the French language, less than three-syllables-long, which could then be used as a congruent cue for that word. These associations were determined through 5 different free-association experiments (see **Free association experiments and word selection)**. Each word in the target stimulus list was associated with only one possible congruent cue and only one possible incongruent cue. Incongruent cues for each target were fixed once for all experiments by shuffling the list of congruent cues and manually verifying the absence of a semantic relation with the corresponding target word.

### Free association experiments and word selection

We conducted two online experiments, in which participants were shown a single visual word and prompted to type in the first word that came to mind in response. They were instructed that they had 15 minutes to respond to 100 words. The first experiment tested 203 words and had between 40 and 67 responders to each item. Data was pre-processed with a semi-automatic spellcheck using the Office suite for Windows. The software detected anomalies, and unequivocal spelling errors were corrected (unintelligible responses were kept as is). Out of these, 137 item pairs were selected with the following criterion: ≥ 25% of responders gave the same association answer to the considered target word. The second experiment tested 95 words and had 70 to 79 responders to each item. Out of these, 73 item pairs were kept. One individual was excluded from analysis, as they provided a single answer to all proposed items. We otherwise followed the methods of Debrenne et al.^40^ Besides these two experiments, we also collected associated pairs of words from a free association online database^40^: we filtered French native speakers, 18 to 25 years old, and selected words matching our criteria. Finally, our stimulus set was completed with existing articles on free association^41,42^ with the same selection criteria. Words were of all grammatical classes, but kept in dictionary form if possible. The resulting stimulus list is available in Supplementary Table 2 and on the OSF repository for this project (https://osf.io/kd2wh/).

### Experimental design

This experiment followed a 2 × 4 factorial design: congruency (2 levels) and target duration (4 levels), plus an extra level of stimulus-absent trials. Each of these 9 conditions included 48 individual trials, split into 15 or 12 blocks, for a total of 432 trials. Participants were informed that the target was absent on some trials. Word-lists for all participants were established prior to the experiments so as to balance cross-trial priming and prior exposure effects (**See Stimulus presentation counter-balancing across participants)**.

### Stimulus presentation counter-balancing across participants

For each participant, 48 words in the general list were removed, corresponding to the target absent trials. The words that were removed were counterbalanced across participants so that each target word in the general list would be absent the same number of times across all participants. Second, since each target word was presented only once per participant (to avoid any prior exposure effect), we made sure that, across all participants, each target would appear the same number of times in each of the different conditions. Third, potential priming from one trial to the next by auditory cues was controlled for by having pairs of participants receive the same auditory cues in the same order, but inverting the congruency relationship between the auditory cues and the preceding visual words (e.g., if for the first participant of a pair, a cue followed the associated congruent target word, for the other participant the same target would be followed by the incongruent cue). Each cue could either appear never, once or twice (in a congruent and an incongruent trial in this case) for each participant. In detail, pseudo-randomization was conducted with a focus on balancing cross-trial word priming: the alphabetical stimulus list was shuffled, then divided into 9 blocks of 48 stimuli. For the first pair of participants, the first block was set as target-absent trials. For participant 1, pseudorandom (balanced over the entire set) congruency, target duration, and feature type were attributed to each non-absent target. Target absent-trials were attributed a pseudorandom target duration. Stimulus order was then shuffled again to mix target-absent trials with target-present trials. Participant 2 was presented with the same twice-shuffled stimulus list, with congruency reversed. The procedure was repeated for 9 participant pairs so that each of the 9 blocks of stimuli could be used as target-absent trials.

### Procedure

In Experiment 1, each trial began with the onset of a circle around the central point, when fixation was successfully maintained for 200 ms using an EyeTracker (see **Materials**). Then, following a 1-2 s jittered delay, 6 random strings of non-alphanumerical characters (picked randomly without replacement among the following: *%#?&/!|±¶*) with the same font and size as target words would rapidly flash on screen with no inter-stimulus interval. These strings would vary both in length (3 to 8 items, all six possible lengths being displayed once in each trial) and duration (two masks of each possible duration: 12, 96 or 192 ms). We used 6 masks so that all possible target word lengths were presented before presentation of the actual target. This was designed to avoid easily detectable variations in image contrast correlated to target word length. A 7th non-alphanumerical mask would follow suit, be 9-characters long (1 character longer than the target to ensure masking it entirely), and last for 200 ms immediately before target onset. Targets would be pseudo-randomly displayed for 12, 24, 36, or 48 ms. There were 11% (48) “target absent” trials where a mask was presented instead of a target, with the same length and duration probability, to later derive the Area under the Receiver Operating Curve (AROC, see **Detection sensitivity, confidence sensitivity, and frequentist analyses**). The target was then followed by an 8th and last mask that was 9-characters long and lasted for 200 ms. All the sequences unfolded with no blank interval between the stimuli. Fifteen milliseconds after the offset of the last mask, i.e., 215 ms after the offset of the visual word target, an auditory cue was presented to the participant. The auditory cue varied in duration depending on spoken word length, and was buffered with silence at the end if necessary for the interval between cue onset and the following event to last for 800 ms. It was either semantically related to the target (congruent trials) or unrelated (incongruent trials). All audio words were pre-recorded with the same female voice and a neutral tone. Participants waited until the offset of the cue (800 ms) before they could respond. They first named the target word aloud into a microphone. If they had not seen any word, they were instructed to provide a random word to the best of their ability. Following this first response, participants had to press the space bar or wait for the end of the 10 second recording delay. They were then prompted to report whether the word was written in upper or lower case using the keyboard in a two-alternative forced choice task. Finally, they had to rate the word’s visibility on a 9-point horizontal scale (a small filled rectangle could be moved within a larger box with ends flanked by the labels “min” and “max”, respectively, to the left and the right. See Supplementary Note 1). Participants were instructed to use the lowest rating when they had not seen the stimulus at all, and to rate overall visibility using the rest of the scale if they had.

In Experiment 2, the procedure was identical to Experiment 1 except for the following: two synchronous streams of masks were presented just above and below a small fixation circle. Simultaneous masks were different strings of symbols of different lengths that lasted the same duration. On each trial, a single target word appeared in one of the streams, while on the other stream, another mask of the same length would be displayed. Note that while there is a known preference for reading in the right visual hemifield^43^ compared to the left, no such strong preference seems to exist between the upper and lower hemifields^44^. While in Experiment 1 the interval between the target offset and the cue onset (ISI) was kept constant at 215 ms, in Experiment 2 and 3 it was the interval between target onset and cue onset (SOA) that was kept constant at 248 ms, with the introduction of a blank period between last mask offset and cue onset with a duration adjusted according to target duration (between 0 and 36 ms). Immediately after the offset of the auditory cue, participants were prompted by a response screen to report where the target had appeared (top or bottom). They then had to provide a visibility rating on this feature using a cursor on a 9-point visual scale similar to Exp. 1, taking into account both detection and position discrimination (Supplementary Note 1). Then they were required to report the target word verbally (recorded via a microphone), and then provide a visibility rating, taking into account both detection and identification.

Experiment 3 was a follow-up from Experiment 1 in which we changed the order of the tasks (to check for potential task order effects) and included confidence ratings on the two tasks, instead of visibility ratings. The sizes of the stimuli were similar to the ones used in Experiment 2, but they were centered, as in Experiment 1. Participants were first prompted to report whether the target was uppercase or lowercase, and how confident they were about this decision on a 9-point scale. Then they were required to report the target word verbally and rate their confidence in their word identification response on a 9-point scale. This time, the scales were used to measure only the confidence in the previous decision; there was no mention of visibility. Participants were asked to use the lowest level when they were entirely not-confident about their decision i.e., respond at random, the first half of the scale if their confidence was low to medium, and the second half if they were fairly to definitely certain of their response.

In Experiments 4-7, the general structure of the design was kept. 18 participants were included in each group according to the criteria listed above; none undertook more than one experiment. The SOA in pre-cueing was 1.6 s with the cue appearing before the series of masks, whereas in retro-cueing, the SOA was 248 ms. Congruent cues were audio tracks of the same word as the target, neutral cues were the same audio tracks reversed (4&5) or pure tones with the same envelope (6&7). All tracks were tested by three independent reviewers to verify they did not resemble French words. The rating scales instructions were provided as in Experiment 2.

### Materials

The materials slightly differed between Experiment 1 and the others. Differences are indicated in parentheses throughout. Stimuli were generated and responses recorded using the Psychophysics Toolbox for Matlab^45^. Stimuli were presented on a CRT monitor, Sony Trinitron GDM-F520 (Sony MULTISCAN E400). Refresh rate was 85 Hz and screen resolution was 1280 by 1024 pixels (1152 by 864). Participants were seated 70 cm away from the monitor (57 cm), in a dimly lit room. Eye fixation was monitored and recorded using an Eye Tribe tracking device (an EyeLink 1000+). Stimuli were presented on a gray background (52.5 cd/m² in Exp. 1; 44.5 cd/m²), and participants were told to fixate a black dot inside a small black circle of 1.6° of visual angle in diameter (0.4°) at the onset of each trial. In all three experiments, the start of a trial was conditioned on appropriate fixation for at least 200 ms (less than 2° from center for Exp. 1; less than 1.2° in other experiments). Targets were words written in a mono-spaced font (Courier New), either in uppercase or lowercase. They were approximately 1° high and 2° to 7.4° large, depending on word-length in Exp. 1 (approximately 0.6° high by 1.6° to 5° large). Note that the entire display was smaller in Experiment 2 to ensure that all stimuli would still be presented within reading range, even though they were displaced from the center of the screen by 0.7°. These changes in size were kept for Experiment 3. A HyperX Cloud headset with a microphone (Kingston Technology Company Inc.) was used to play the auditory words (retro-cues) and record participants’ verbal responses. Audio-visual delay was measured using a photodiode applied to the computer screen and the computer’s audio output, together with an oscilloscope for visualization. The delay was within a range of 10 ms with no identifiable jitter. This delay was taken into account in the stimulation scripts.

### Data preprocessing

Trial exclusion criteria: For Experiments 1, 2 & 3, trials were excluded when the verbal response was null or inconsistent with instructions, or if expected timings during the trials were not respected. Word identification was performed orally to avoid spelling mistakes had they used the keyboard, and guessing with the help of the auditory cue had they been presented with multiple choices. Audio tracks from each participant were transcribed manually. Trials were rejected when participants were unintelligible, did not provide a verbal response, or responded with a variation of “I don’t know”, which they were prompted never to say. The average proportion of rejected trials in total was 4 ± 8% (including one outlying participant with 31% rejected trials, who did not comply with the instruction of always providing an answer at the end of a trial. All analyses were also conducted without said outlier, but led to the same conclusions: they were kept for analyses), 3.7 ± 2.8% in Experiment 2, and 1.1 ± 0.9% in Experiment 3, mostly due to participants not responding on some trials. No other exclusions were made on the basis of response times, as the time order of tasks varied in these protocols, and we preferred keeping a constant analysis plan. In Experiments 4–7, however, since probes occurred in the same order without verbal responses, we could exclude trials in which participants took over 2.5 median absolute deviation from the median response time to answer under the assumption that these trials corresponded to lapses in attention. This led to the exclusion of <5% of trials in all participants.

### Modeling of word identification and correction for guessing

We analyzed participants’ accuracies at identifying the masked word and discriminating its visual features (e.g., case or position) with a logistic regression^46^. Given that our dataset is grouped according to ‘observational units’ (participants), which could be considered as a random sample from a population, we adopted a hierarchical Bayesian approach. For word identification, the probability of identifying the word during a given trial is a mixture of true hits and guessed responses (heard “hive”, tried “bee” without having seen the word). In congruent trials, these guesses correspond to the correct answer and inflate performance, but not in incongruent trials. Under the assumption that participants are about as likely to guess in congruent and incongruent trials when visual information is scarce, we identified guess trials in the incongruent condition based on whether participants had reported the word associated with the cue, and treated them as correct answers when modeling, so as to bring both conditions to a comparable level, before removing the guesses from both, for visualization. The logistic regression used was of the form:

$$p\left(y=1\right)=\frac{1}{1+{e}^{-\left({\beta }_{0}{+\beta }_{1}.{congruency}+\left({\beta }_{2}{+\beta }_{3}.{congruency}\right).{duration}\right)}}$$

where p(y = 1) corresponds to the probability of correct responses, “duration” corresponds to the duration of the target, and “congruency” is either 0, for incongruent cues, or 1 for congruent cues. This corresponds to a standard logistic regression model estimating the effect of congruency, target duration, and their interaction while including an intercept to capture the estimated guess rate at target duration 0. We also included subject-specific random effects for all β parameters as well as word-specific random effects for the intercept and target duration, assuming that words of varying length and lexical frequency could be more or less difficult to identify with little visual information. All coefficients in the model were given standard priors with the help of the R package Rstanarm^47^.

Supplementary Fig. 1 shows that the proportion of guesses decreased in incongruent trials as a function of target duration. We observe that this proportion decreases as the duration of the visual target increases: when more information about the visual target is made available to the participant, thanks to longer target durations, it becomes less likely that they will guess using the information of the cue alone. The guess rate estimated from incongruent trials (guesses in congruent trials are mixed with true hits and cannot be identified) was fitted using a logistic regression estimating the effect of target duration and an intercept on the probability of a guess, with participant-specific random effects for both coefficients. Using a likelihood-ratio test^48^ we assessed that including target duration significantly improved the model fit vs. an intercept-only model in all experiments (Exp. 1: χ²(1) = 66.488, *p* < 0.001, Exp. 2: χ²(1) = 11.336, *p* < 0.001, Exp. 3: χ²(1) = 49.85, *p* < 0.001).

For visual representations of performance corrected for guess-rate in the figures throughout the article, we subtracted, separately for each target duration and each participant, the estimated guess rate in incongruent trials (% of trials where they reported the word associated with the cue) from the mean performance rate at each duration, and from the performances estimated by the model.

### Modelling of case (or position) discrimination

The same model and procedure were used for case or position discrimination performance, except for the correction for guesses, which was irrelevant. For Experiments 4 through 7, the same approach was used, but also included the type of cue (pre- or retro-) and all possible interactions between the three main effects (duration, congruency, type of cue).

Estimates from the model or their Bayesian credible intervals (BCI) are reported as odds-ratios (BCI-Odds Ratio). Note that odds ratios represent multiplicative changes on the response: for example, an interval of [5–20] for the effect of congruency indicates that the probability of a correct response was between 5 and 20 times more likely in the congruent condition than the incongruent one. Conversely, an interval of [0.1–0.5] indicates that the probability of a correct response was 0.1 to 0.5 times more likely, in other words, 2 to 10 times less likely. Consequently, if the interval contains 1, the effect is considered not significant. Pointwise estimates of the effect of congruency at various target durations, however, are expressed as a difference in expected accuracy (BCI-diff): an interval of [0.2–0.4] for the difference between congruent and incongruent conditions indicates that the probability of a correct response was increased by 0.2 to 0.4. Consequently, if the interval contains 0, the effect is considered not significant.

### Model fitting

The model was implemented in Stan^49^ with the help of the Rstanarm package^47^. Markov Chain Monte Carlo (MCMC) sampling was used to draw samples from the posterior distribution of model parameters. The model was fitted separately for each experiment, and the quality of fit was assessed by running 4 sampling MCMC chains and comparing the variance across and within chains with R-hat. In all models fitted, all R-hat statistics were < 1.01, indicating appropriate convergence (see Supplementary Table 3 for diagnostic tables of all model fits). We derived predictions for the expected effect of congruency by marginalizing over the distribution of word-specific random effects.

### Detection sensitivity, confidence sensitivity and frequentist analyses

Detection sensitivity and confidence sensitivity were derived from the distributions of visibility or confidence ratings, respectively, using Signal Detection Theory^50,51^. AROCs were computed by comparing distributions of ratings on target-present trials in each considered condition to ratings provided in target-absent trials, using the following relationship:

$${AROC}=\frac{U}{{n}_{1}* {n}_{2}}$$

Where U is the result of a Wilcoxon signed-rank test on two independent samples of respective sizes n_1_ and n_2_. These AROC measures were then analyzed using frequentist statistics. The full distributions of visibility and confidence ratings for all conditions can be found in the supplementary material.

### Frequentist analyses and parametric statistical testing

The data were visually inspected for deviation from a Normal distribution, and heteroscedasticity was tested for using a Bartlett test. Where ANOVAs were used in the text, the data were normally or near-normally distributed, and the Bartlett test was non-significant. Reported effect sizes are generalized eta-squared (η²G, < 0.01 is small, > 0.14 is large^52^) for ANOVAs and Cohen’s d (*d*, < 0.2 is small, > 0.8 is large^53^) for pointwise mean differences. Wherever necessary, we used Bonferroni corrections for multiple comparisons. All tests were two-sided unless stated otherwise.

### Conditional probability analysis: estimating case or position performance in trials with correct word identification

For the conditional performance panels, performance at the case or position task was calculated for trials where the word was correctly identified. In the congruent condition, this required discarding “correct-guesses” trials in the congruent condition where they cannot be easily identified. We calculated an individual, per target duration, visibility cut-off below which a correct trial could safely be classified as a guess (since the response was correct but the subjective rating was low). If a participant’s point-estimate of the guess rate (based on the guesses identified in the incongruent condition) was n% of congruent trials, we excluded all trials with visibility equal to or below that of the nth percentile. This cut-off was most of the time between visibility 0 and visibility 1 (0.27 ± 0.46 for 12 ms, 0.47 ± 0.64 for 24 ms, 0.74 ± 0.88 for 36 ms, 0.87 ± 0.64 for 48 ms for Experiment 1).

### Levenshtein distance analysis

To compute string distance in partial awareness controls, we calculated the Levenshtein distance by means of the Wagner-Fischer algorithm^54^. This distance between a string A and a string B is the number of substitutions, insertions, or deletions of individual letters needed to turn string A into string B. We used this metric because it is more specific than position-independent letter-matching (is there an “a” in A and B), and less specific than position-dependent letter matching (is there an “a” in position 2 in strings A and B), as it accounts for position in-word (it accounts for fragments of more than one letter, e.g. bigrams or trigrams) but allows for character insertion and deletion. This distance was then divided by the total number of letters in the target word to obtain reasonably comparable measures. For these analyses, the Bartlett test for heteroscedasticity was significant; however, there is no non-parametric equivalent to a two-way ANOVA. We still conducted two-way ANOVAs as it was the best approximation to the desired analyses, but it should be noted that assumptions were not fully met.

### Pre-registrations

The experimental protocol and hypotheses tested were pre-registered for Experiments 4/5 on Jul 27, 2021, and Experiments 6/7 on Jun 28, 2024. The proposed design and analysis plan was followed except for the conduction of Bayesian analyses on case discrimination performance, which was eventually conducted on all 4 experiments pooled rather than pairs.

## Results

Experiments 1 to 3 investigated the effect of retrospective semantic cues on the perception of masked visual words at different levels: identification of their meaning, discrimination of lower-level visual features, and visibility/confidence associated with such perception. Experiments 4 to 7 were designed to control for additional factors, including the time-order effect of the cue (pre- vs. retro-cueing).

### Experiment 1: Effect of retrospective cueing on detection, identification, and case discrimination of masked words

**Fig. 2:**
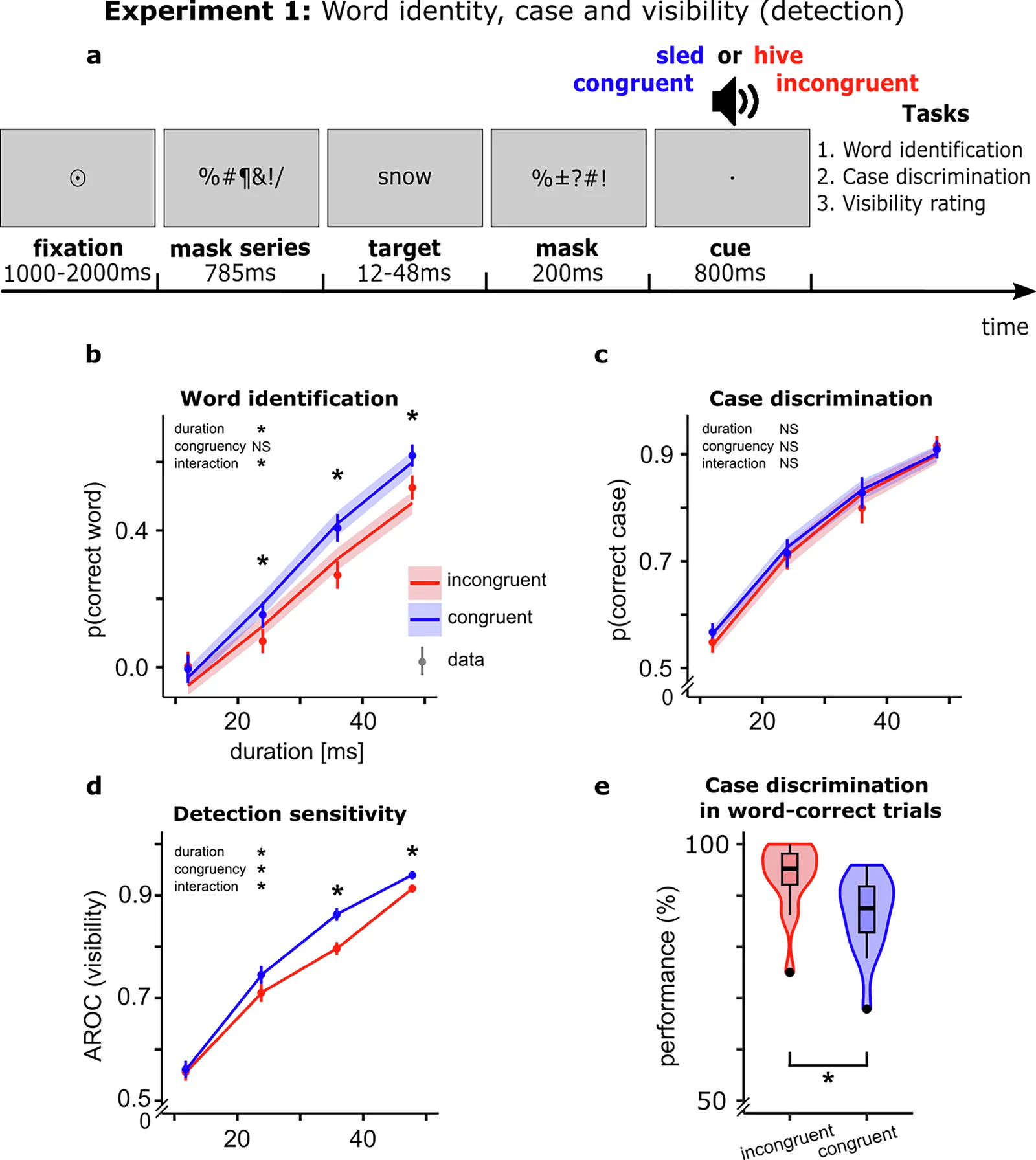
Experiment 1. **a** Participants were presented with one stream of centered stimuli. Response screens asked for word identification, case discrimination and overall visibility rating, in this order. **b** Word identification performance, corrected for guessing (see text for details): filled circles correspond to the data; lines represent performance estimated by the model. Error bars are the standard error (SE) of the mean across observers (*n* = 15), and error bands are the SE of model predictions across observers (*n* = 15). **c** Case discrimination performance, symbols are as in (**b**) (*n* = 15). **d** Detection sensitivity derived from the AROC of visibility ratings: symbols are as in (**b**) except error bars represent standard errors of the mean across observers (*n* = 15). **e** Case discrimination performance when word identification is correct, corrected for guessing (*n* = 15). Thick black horizontal lines represent median values; each box plot ranges from the 25th to the 75th percentile of the distribution, whiskers extend to 1.5 interquartile range at each end, and outliers are shown as black circles. A violin plot is overlaid to show the distribution of the data. In all panels, stars indicate the significance of the statistical tests as described in the text. Deep blue items correspond to congruent trials, red to incongruent trials.

The protocol of Experiment 1 is illustrated in Fig. 2a and described in the **Methods** section.

#### Word identification

Figure 2b shows the datapoints corrected for guess rate and the model’s estimates for word identification performance (see Methods for details). Our model revealed a positive main effect of target duration (95%BCI-Odds Ratio [1.51, 2.08]), no main effect of congruency (95%BCI-Odds Ratio [0.71, 1.39]), and a significant interaction between target duration and congruency, with the congruency effect increasing with increasing target duration (95%BCI-Odds Ratio [1.02, 1.36]). On post-hoc comparisons for each target duration, a significant effect of congruency was found for all target durations above 12 ms (all the results of post-hoc statistics can be found in Supplementary Note 2).

#### Case discrimination

Modeling performance for case discrimination (Fig. 2c) revealed a positive effect of target duration on performance (95% BCI-Odds Ratio [1.88, 2.52]), but no effect of congruency (95%BCI-Odds Ratio [0.82, 1.50]), nor any interaction between these factors (95%BCI-Odds Ratio [0.86, 1.13]), in sharp contrast with what was observed for word identification.

#### Detection sensitivity

We next estimated participants’ detection sensitivity by comparing the distributions of visibility ratings for target present versus target absent trials (AROC, see Methods for details) (Fig. 2d). A repeated measures two-way ANOVA revealed a significant increase in detection sensitivity with target duration (F(3.00, 42.00) = 92.00, *p* < 0.001, η²_G_ = 0.73, 95% CI for η²_G_ [0.55 0.80]), and congruency (F(1.00,14.00) = 15.79, *p* = 0.001, η²_G_ = 0.04, 95% CI for η²_G_ [0.00 0.22]) with an interaction between these two factors (F(3.00,42.00) = 3.62, *p* = 0.02, η²_G_ = 0.02, 95% CI for η²_G_ [0.00 0.08]). Bootstrapped 95% confidence intervals of the mean difference between congruent and incongruent conditions revealed a significant increase in detection sensitivity when the retro-cue is congruent for target durations of 36 ms and 48 ms, and a trend in the same direction for target durations of 24 ms (Supplementary Note 2). This pattern of results suggests that congruent retro-cues improves participants’ ability at detecting the target.

Supplementary Fig. 2 provides a similar analysis on mean visibility ratings, and shows the full distributions of visibility ratings in the different conditions.

Of note, in this experiment, performances for case discrimination and detection were overall slightly higher than in the other similar experiments (experiments 3, 5 and 7), notably with performances at the shortest stimulus duration that were significantly better than chance (t(14) = 2.99 *p* = 0.009, *d* = 0.77, 95%CI for *d* [0.34 1.44] for detection, and t(14) = 3.6454 *p* = 0.003, *d* = 0.94, 95%CI for *d* [0.44 1.99] for case discrimination). This suggests that participants in this experiment might have lower thresholds overall (the psychometric curves are displaced towards lower durations) compared to the others. This difference could be due to the fact that, for this first experiment, participants came for a screening session that also constituted a 1-hour training (although on a different set of words; see **Methods)**.

#### Controlling for partial awareness: conditional probabilities between tasks

The discrepancy between detection sensitivity/word identification and case discrimination was consistent with the hypothesis that retro-cues might induce conscious access to semantic features after the mask has degraded its low-level sensory representations. However, an alternative interpretation, in terms of partial awareness, was also possible: participants may have sometimes retrieved a few letters from a masked word, even if they did not perceive it in full^55^. In this scenario, congruent retro-cues did not act by retrospectively improving awareness of the target’s semantic attributes, but instead by allowing to combine a semantic information with existing partial awareness of some letters. For example, if participants had partial awareness of the letters “edg” and heard the retro-cue “porcupine”, they could efficiently combine both pieces of information to infer “hedgehog”.

These interpretations can be sorted out by analyzing conditional performance; if congruent retro-cues induce conscious access to the word meaning after the mask has erased information about its case, then case discrimination on these trials should be poor, and hence case discrimination for word correct trials should be worse for congruent than incongruent retro-cues. In contrast, this should not be observed if congruent retro-cues help identify the target on trials where people were already aware of a few letters.

As shown in Fig. 2e, performance for case discrimination on “word-correct” trials was very high for incongruent cues (92.9%), suggesting that, when participants reported the word’s identity in this condition, they knew most of the time what the word’s case was. It significantly decreased for congruent cues, with 86.3% correct case discrimination on “word-correct” trials (see Methods for how these trials were corrected for guesses; mean difference – 7.21, 95%CI of the mean difference in %correct = [−12.76, −2.39], *d* = −1.01, 95%CI for *d* [−2.01 -0.3]), confirming a dissociation of the effect of congruency on identification versus case discrimination.

#### Controlling for orthographic partial awareness: orthographic distance in error trials

We conducted an additional analysis to control for a more subtle version of the partial awareness hypothesis: participants might sometimes be aware of a few letters not in their visual form, but in a more abstract orthographic form. For example, participants might be aware that the triplet of letters “e, d and g” was presented without having seen their visual attributes; this information might nevertheless combine with the congruent semantic retro-cue to produce successful identification.

We tested for this possibility by examining the orthographic distance between participants’ response and the actual target in error trials, using the Levenshtein distance (**see Methods**). Indeed, if orthographic partial awareness exists in our protocol, then participants’ responses should include these orthographic fragments even when they fail to correctly identify the word. For example, if they have perceived “edg” they might report “knowledge” instead of “hedgehog”. Furthermore, if improved word identification with congruent retro-cues was due to these trials, then their proportion among error trials should decrease for congruent compared to incongruent retro-cues.

As shown in Supplementary Fig. 3, orthographic distance in error trials decreased significantly with increasing target duration (F(3,42) = 40.16, p < 0.001, η²G = 0.42, 95%CI for η²G = [0.15 0.56]), confirming that there was some partial orthographic awareness in this protocol, and that it increased with increasing target duration. However, there was no evidence of a statistically significant effect of congruency on this orthographic distance (F(1,14) = 0.05, *p* = 0.82, η²G = 0.0003, value too low for estimating CI) and no evidence of a statistically significant interaction between congruency and target duration (F(3,42) = 1.055, *p* = 0.38, η²G = 0.02, 95%CI for η²G = [0.00, 0.07]), suggesting that the congruency effect we observed on identification did not rely on partial orthographic awareness.

#### Conclusion

In conclusion, we observed that congruent retro-cues improved participant’s identification as well as their sensitivity to detect the presence of the words (Fig. 2b, d), without improving their ability to report their case (Fig. 2c). We made sure that this effect could not be accounted for by partial awareness. Next, we conducted two additional experiments with small variations from this protocol in order to test the replicability of these effects and their generality. We also took advantage of these experiments to further characterize the associated subjective experience. In Experiment 2 we changed the visual task from case to position discrimination, and in Experiment 3 we collected ratings about the confidence in the response instead of the visibility of the word.

### Experiment 2: effect of retrospective cueing on detection, identification, and position discrimination of masked words

**Fig. 3:**
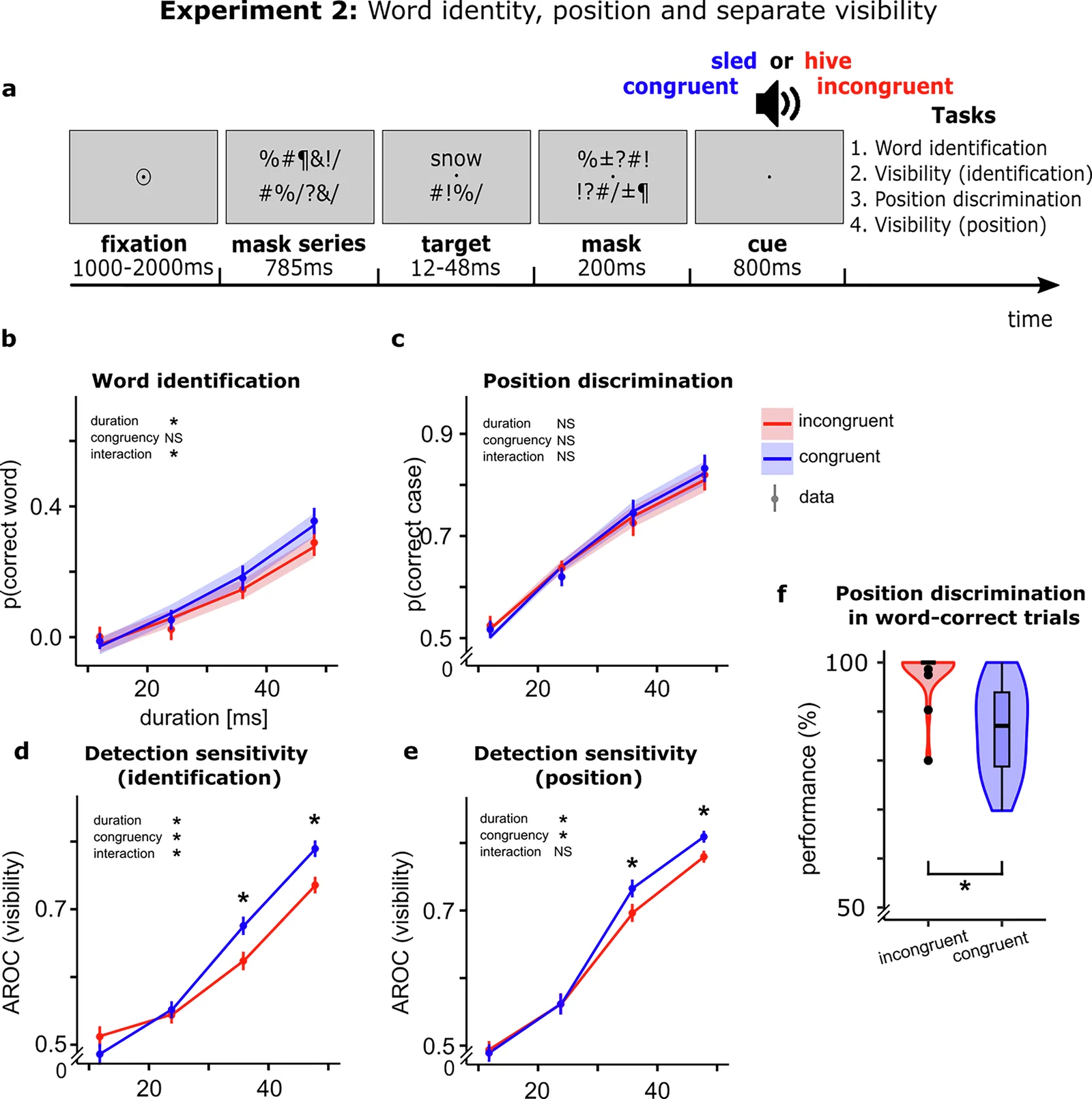
Experiment 2. **a** Participants were presented with two streams of stimuli above and below fixation. Response screens asked for position discrimination, visibility on position, word identification, and visibility on word identity, in this order. **b** Word identification performance, corrected for guessing (see text for details): filled circles correspond to the data; lines represent performance estimated by the model. Error bars are standard error (SE) of the mean across observers (*n* = 18), and error bands are the SE of model predictions across observers (*n* = 18). **c** Position discrimination performance: symbols are as in (**b**) (*n* = 18). **d** Detection sensitivity derived from the AROC of visibility ratings for word identification: symbols are as in (**b**), error bars are standard errors of the mean across observers (*n* = 18). **e** Detection sensitivity derived from the AROC of visibility ratings for position discrimination: symbols are as in (**d**) (*n* = 18). **f** Position discrimination performance when word identification is correct (*n* = 18). Thick black horizontal lines represent median values; each box plot ranges from the 25th to the 75th percentile of the distribution, whiskers extend to 1.5 interquartile range at each end, and outliers are shown as black circles. A violin plot is overlaid to show the distribution of the data. In all panels, stars indicate the significance of the statistical tests as described in the text. Deep blue items correspond to congruent trials, red to incongruent trials.

In Experiment 2 we tested whether the effects found in Experiment 1 extended to another fundamental visual attribute: the position of the visual word on screen (Fig. 3a). The response screens prompted for position discrimination first, followed by a visibility rating specific to the position of the target, then participants reported on word identification, and last they gave a visibility rating specific to word meaning (see Supplementary Note 1). This exploratory manipulation aimed at testing whether participants could distinguish over which dimension their subjective visibility increased. If they could, the prediction was that the effect of congruency on subjective visibility and derived detection sensitivity should only be observed for word identification, not position. However, we were also interested in another possibility, that participants might have had an illusion of seeing the low-level feature when they felt they detected the stimulus, in which case this dissociation in subjective visibility along the two dimensions should not be present.

Eighteen participants took part in this experiment. When analyzing the results, we first made sure that participants were not biased into attending a specific stream by analyzing their position response on target-absent trials. Participants' absolute deviation from an ideal 50% of each type of response in catch trials was 14.58 ± 9.20% (maximum deviation 31.25%), presumably reflecting individual biases towards one stream or the other in the absence of an overt strategy.

#### Word identification

The main analyses were conducted as in Experiment 1 (see **Methods**): for word identification (Fig. 3b), we evidenced a positive effect of target duration (95%BCI-Odds Ratio [1.34, 1.80]), and a significant interaction between congruency and target duration (95%BCI-Odds Ratio [1.002, 1.27]) but no main effect of congruency (95%BCI-Odds Ratio [0.63 1.19]). None of the post-hoc pointwise comparisons at each target duration reached significance (the results of all post-hoc comparisons can be found in Supplementary Note 2). This pattern of results suggests that the interaction effect on word identification was present in Experiment 2 but was less strong than in Experiment 1.

#### Position discrimination

For position discrimination (Fig. 3c), performance increased significantly with longer target durations (95%BCI-Odds Ratio [1.78, 2.24]), but there was no effect of congruency (95%BCI-Odds Ratio [0.91, 1.56]) and no interaction (95%BCI-Odds Ratio [0.87 1.09]), suggesting that, in contrast to word identification, congruency had no effect on position discrimination.

#### Detection sensitivity

Detection sensitivity derived from the first visibility scale, related to word identification (Fig. 3d), showed a significant effect of target duration (F(3.00, 51.00) = 38.84, *p* < 0.001, η²_G_ = 0.48, 95% CI for η²_G_ [0.25, 0.60]), congruency (F(1.00, 17.00) = 9.24, p = 0.007, η²_G_ = 0.01, 95% CI for η²_G_ [0.00, 0.11]), and a significant interaction (F(3.00,51.00) = 8.50, *p* < 0.001, η²_G_ = 0.02, 95% CI for η²_G_ [0.00, 0.10]). Post-hoc pointwise comparisons at each target duration showed a significant increase in detection sensitivity with congruency at durations 36 ms and 48 ms (Supplementary Note 2). Detection sensitivity analysis for the other visibility scale, related to word position (Fig. 3e), showed a significant effect of target duration (F(3.00,51.00) = 48.61, *p* < 0.001, η²_G_ = 0.54, 95% CI for η²_G_ [0.32 0.65]), a significant yet small effect of congruency (F(1.00,17.00) = 7.53, *p* = 0.01, η²_G_ = 0.005, 95% CI for η²_G_ [0.00 0.07]), and no evidence of an interaction (F(3.00,51.00) = 2.35, p = 0.08, η²_G_ = 0.006, 95% CI for η²_G_ [0.00, 0.02]). Pointwise comparisons at each target duration showed an increase in detection sensitivity with congruency at durations 36 ms and 48 ms (Supplementary Note 2). Finally, we conducted a 2-way repeated measures ANOVA on mean difference in detection sensitivity between congruent and incongruent conditions, by target duration and type of visibility rating (related to word identification or position discrimination) to assess whether congruency affected detection sensitivity differently according to type of stimulus dimension that was emphasized. This analysis found no evidence of a statistically significant effect of type of task (F(1.00,17.00) = 0.52, *p* = 0.48, η²_G_ = 0.004, 95% CI for η²_G_ [0.00 0.06]), nor of an interaction between task and target duration (F(3,51) = 1.72, p = 0.17, η²_G_ = 0.02, 95% CI for η²_G_ [0.00 0.10]).

Supplementary Fig. 4 provides a similar analysis on mean visibility ratings, and shows the full distributions of visibility ratings in the different conditions.

#### Controlling for partial awareness: conditional probabilities between tasks

Position discrimination performance in word-correct trials (Fig. 3f) decreased from 98.5% in incongruent trials to 85.7% in congruent trials (mean difference congruent minus incongruent: −11.88%, 95%CI [−15.87 −8.45], *d* = −1.60, 95%CI for *d* [-2.74, −0.87]), arguing against an interpretation in terms of partial awareness of physical attributes.

#### Controlling for orthographic partial awareness: orthographic distance in error trials

We next tested if partial orthographic awareness of the target word could account for our results. As explained in Experiment 1, under this hypothesis, the proportion of errors that share orthographic constituents with the masked word should decrease for congruent compared to incongruent retro-cues, hence increasing the orthographic distance between the erroneous responses and the true responses. As shown in Supplementary Fig. 3, no such effect was observed: the repeated measures ANOVA (duration x congruency) showed an effect of duration (F(3,51) = 3.58, *p* = 0.02, η²G = 0.05, 95%CI for η²G = [0.00 0.15]) but no evidence of a significant effect of congruency (F(1.00,17.00) = 0.209, p = 0.65, η²G = 0.001, 95%CI for η²G = [0.00 0.04]) nor evidence in favor of an interaction (F(3,51) = 1.15, *p* = 0.34, η²G = 0.02, 95%CI for η²G = [0.00 0.09]).

#### Conclusion

In conclusion, Experiment 2 replicated the effect of retrospective cueing on word identification and word detection observed in the previous experiment, and extended the dissociation between word identification performance and discrimination of visual features to the spatial location of the stimulus. Overall performance as well as the effect of congruency was reduced at the word-identification task, possibly due to participants attending two locations on screen compared to one in Experiment 1. The results obtained when distinguishing visibility ratings for identification and for position could have different interpretations, an issue we address in Experiment 3.

### Experiment 3: effect of retrospective cueing on identification, case discrimination, and confidence in both tasks

We designed Experiment 3 to provide an additional replication of our initial results (Experiment 1) and probe a possible distinction between subjective visibility ratings and confidence ratings. Indeed, in Experiment 2, detection sensitivity, as measured with visibility ratings, increased similarly with congruent retro-cues for both visual (position) and more abstract (semantic) dimensions of the stimulus, contrasting with the dissociated effects of congruency on word identification versus position discrimination. This could have two interpretations: either improved perception of the word identity with retro-cues was accompanied by an illusion of better perceiving its visual features, or the similarity of the instructions for both visibility ratings induced this similarity in responses (notably, both scales shared instructions regarding visibility “0”, see Supplementary Note 1). Here we addressed this issue by changing visibility scales for confidence scales, with each scale being dedicated to judging confidence over either the identification response or the case response. If the similar patterns obtained for visibility ratings were due to overlapping instructions, these well-separated confidence ratings should instead reflect the dissociation observed in first-order performance.

**Fig. 4:**
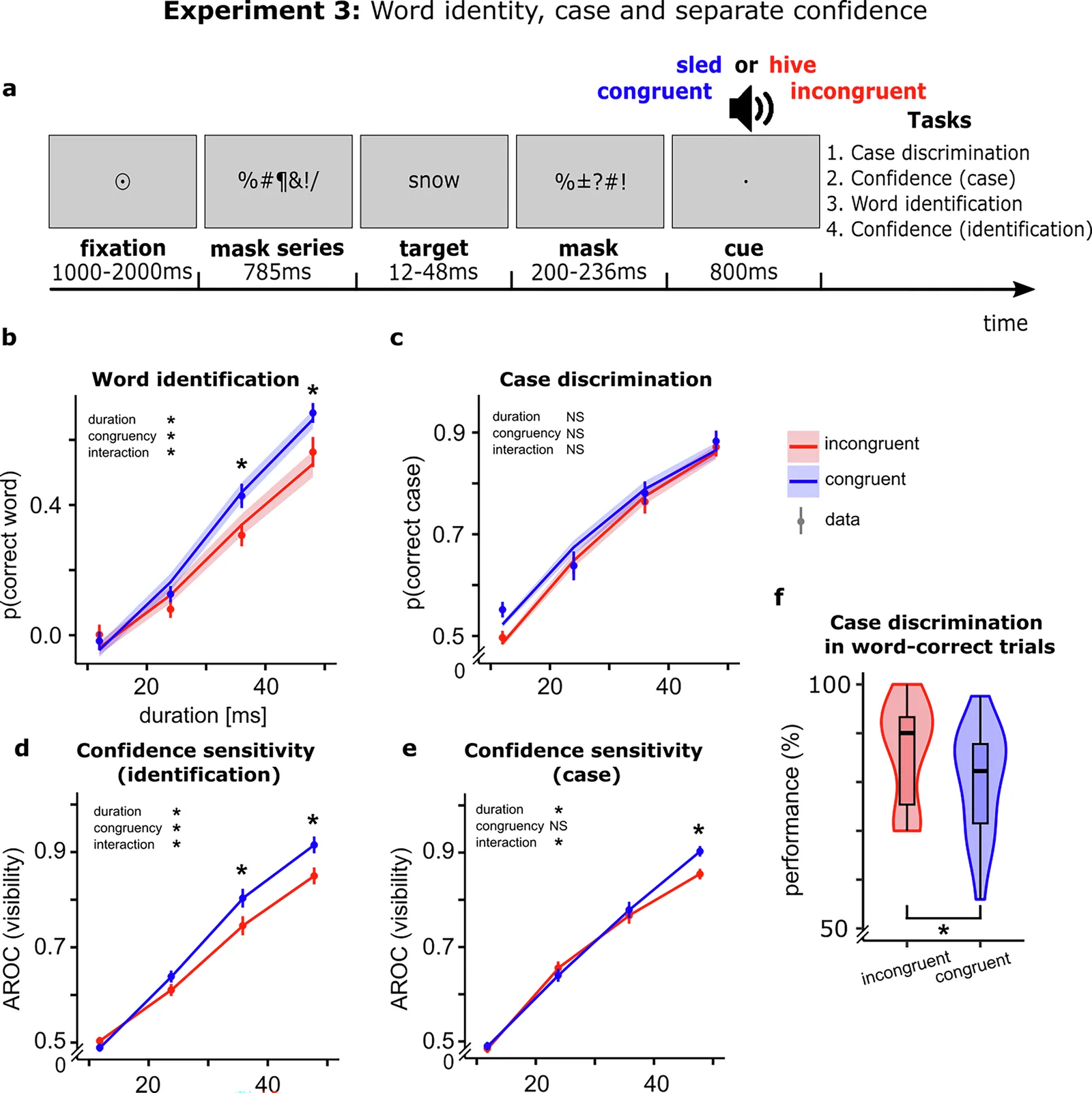
Experiment 3. **a** Participants were presented with a single centered stream of stimuli. Response screens asked for case discrimination, confidence on case, word identification, and confidence on word identity, in this order. **b** Word identification performance, corrected for guessing (see text for details): filled circles correspond to the data; lines represent performance estimated by the model. Error bars are standard error (SE) of the mean across observers (*n* = 18), and error bands are the SE of model predictions across observers (n = 18). **c** Case discrimination performance: symbols are as in (**b**) (*n* = 18). **d** Detection sensitivity derived from the AROC of visibility ratings for word identification: symbols are as in (**b**), error bars are standard errors of the mean across observers (*n* = 18). **e** Detection sensitivity derived from the AROC of visibility ratings for position discrimination: symbols are as in (**d**) (*n* = 18). **f** Case discrimination performance when word identification is correct (*n* = 18). Thick black horizontal lines represent median values; each box plot ranges from the 25th to the 75th percentile of the distribution, whiskers extend to 1.5 interquartile range at each end, and outliers are shown as black circles. A violin plot is overlaid to show the distribution of the data. In all panels, stars indicate the significance of the statistical tests as described in the text. Deep blue items correspond to congruent trials, red to incongruent trials.

The structure of the experiment was the same as in Experiment 1, as illustrated in Fig. 4a, except that, at the end of each trial participants had to report the case of the target word, followed by their confidence in this response, and then they had to report the word identity in a microphone, followed by their confidence in this response. On both confidence scales, “0” corresponded to an absence of confidence in the corresponding response, regardless of whether they perceived the stimulus or not (see instructions in Supplementary Note 1).

#### Word identification

The main analyses were conducted as in Experiment 1: for word discrimination (Fig. 4b), there was a positive effect of target duration (95%BCI-Odds Ratio [1.75 2.65]), congruency (95%BCI-Odds Ratio [1.02 1.50]), as well as a significant interaction between these two factors (95%BCI-Odds Ratio [1.16 1.50]). Post-hoc pointwise comparison at each target duration showed an increase in performance with congruency at durations above 24 ms (Supplementary Note 2).

#### Case discrimination

For case discrimination (Fig. 4c), we found an effect of target duration (95%BCI-Odds Ratio [1.45, 1.92]), but no effect of congruency (95% BCIs: Odds Ratio [0.69, 1.16]) and no interaction (95% BCIs: Odds Ratio [0.95, 1.18]).

#### Confidence sensitivity

Confidence sensitivity analysis for word identification (Fig. 4d) showed a significant effect of target duration (F(1.03,17.64) = 121.19, *p* < 0.001, η²_G_ = 0.73, 95% CI for η²_G_ [0.58, 0.80]), congruency (F(1.00, 17.00) = 14.90, *p* = 0.001, η²_G_ = 0.04, 95% CI for η²_G_ [0.00, 0.20]), and a significant interaction (F(3.00, 51.00) = 6.26, *p* < 0.001, η²_G_ = 0.03, 95% CI for η²_G_ [0.00, 0.11]). Pointwise comparisons at each target duration showed an increase with congruency at durations 36 ms (Supplementary Note 2) and 48 ms.

Confidence sensitivity analysis for the case discrimination response (Fig. 4e) showed an effect of target duration (F(3.00,51.00) = 96.94, p < 0.001, η²_G_ = 0.67, 95% CI for η²_G_ [0.51 0.76]) and an interaction (F(2.17,36.98) = 4.45, p < 0.001, η²_G_ = 0.01, 95% CI for η²_G_ [0.00 0.06]), but no evidence of a main effect of congruency (F(1.00,17.00) = 2.84, p = 0.11, η²_G_ = 0.004, 95% CI for η²_G_ [0.00 0.06]). Pointwise comparisons at each target duration showed an increase in case confidence sensitivity with congruency at duration 48 ms (Supplementary Note 2). This suggests a smaller effect of congruency on confidence sensitivity in this case, in the form of an interaction where confidence on case discrimination appears to increase only at the longest target duration, together with no evidence of an effect for less visible stimuli.

Finally, we conducted a 2-way repeated measures ANOVA on the mean difference in confidence sensitivity between congruent and incongruent conditions, by target duration and type of visibility rating (related to word identification or position discrimination), to assess whether congruency affected confidence sensitivity differently according to the dimension that was emphasized. This analysis revealed a main effect of type of confidence scale (F(1.00, 17.00) = 8.30, *p* = 0.01, η²_G_ = 0.03, 95% CI for η²_G_ [0.00, 0.03]), as well as an interaction between type of confidence scale and target duration (F(3,51) = 7.79, p < 0.001, η²_G_ = 0.005, 95% CI for η²_G_ [0.00, 0.009]) on this congruency effect. This contrasts with the absence of evidence for an interaction between type of task and congruency in Experiment 2.

Supplementary Fig. 5 provides a similar analysis on mean confidence ratings, and shows the full distributions of visibility ratings in the different conditions. A closer look at these distributions shows that the slight effect observed at 48 ms for confidence sensitivity relates to an increase in confidence for ratings that were already confident (from confidence 6 to confidence 8), not a change from low confidence to high confidence, in stark contrast with the distributions observed for word identification confidence.

Overall, these results show that when participants were asked to rate their confidence separately on each dimension, their ratings reflected the differential effect of congruency on identification versus case discrimination, suggesting that, although congruent retro-cues made them detect the stimulus presence better, this was not accompanied by an illusion of confidence about their ability to report the word’s case. The similar patterns observed over the two dimensions when using visibility ratings in Experiment 2 were thus probably due to a partial overlap in the instructions for reporting visibility over the two dimensions.

#### Controlling for partial awareness: conditional probability analysis

Case discrimination performance corrected for guessing in word-correct trials (Fig. 4f) was significantly decreased in congruent trials compared to incongruent trials (mean −6.27%, 95%CI [−10.31 −2.47], *d* = −0.58, 95%CI for *d* [−1.33 0.05]).

#### Controlling for orthographic partial awareness: orthographic distance in error trials

As in previous experiments, we observed a decrease in orthographic Levenshtein distance in error trials with target duration (F(2.17,36.93) = 22.790, *p* < 0.001, η²G = 0.26, 95%CI for η²G = [0.05 0.40]), but no evidence of an effect of congruency (F(1.00,17.00) = 1.891, *p* = 0.19, η²G = 0.008, 95%CI for η²G = [0.00 0.09]) nor evidence of an interaction (F(1.68,28.57) = 0.960, *p* = 0.39, η²G = 0.01, 95%CI for η²G = [0.00, 0.07]) (Supplementary Fig. 3), contrary to what would be expected if the effect of congruent retro-cues relied on orthographic partial awareness.

#### Conclusion

In conclusion, this experiment replicated our initial results and showed that retro-cueing improved word identification without improving case discrimination. Furthermore, while Experiment 2 showed that detection sensitivity for both types of ratings increased in congruent trials, we found a different pattern for confidence sensitivity.

### Experiments 4 through 7: comparing the effect of pre-cues and retro-cues

In these pre-registered (https://osf.io/cnf68/ and https://osf.io/8rfw7/) experiments, we tested whether performance dissociation between high-level and low-level information was specific to retrospective cueing. Our main hypothesis would predict so, since we postulated that this dissociation arises because the retro-cue triggers conscious access after low-level features have been disrupted by masking. However, as mentioned earlier, an alternative interpretation of our results could be that the cue does not change the awareness status of the various representations of the stimulus, but only allows better identification and detection performance by combining partial information on the word with a semantic context. If this were the case, then the temporal order of the visual and auditory presentation should not matter: in both cases, the combination of information should occur, producing the same dissociation. Alternatively, if the retro-perception interpretation is correct, we predicted that the dissociation should be unique to retro-cueing, while pre-cueing should alleviate masking at both the low and high levels.

**Fig. 5:**
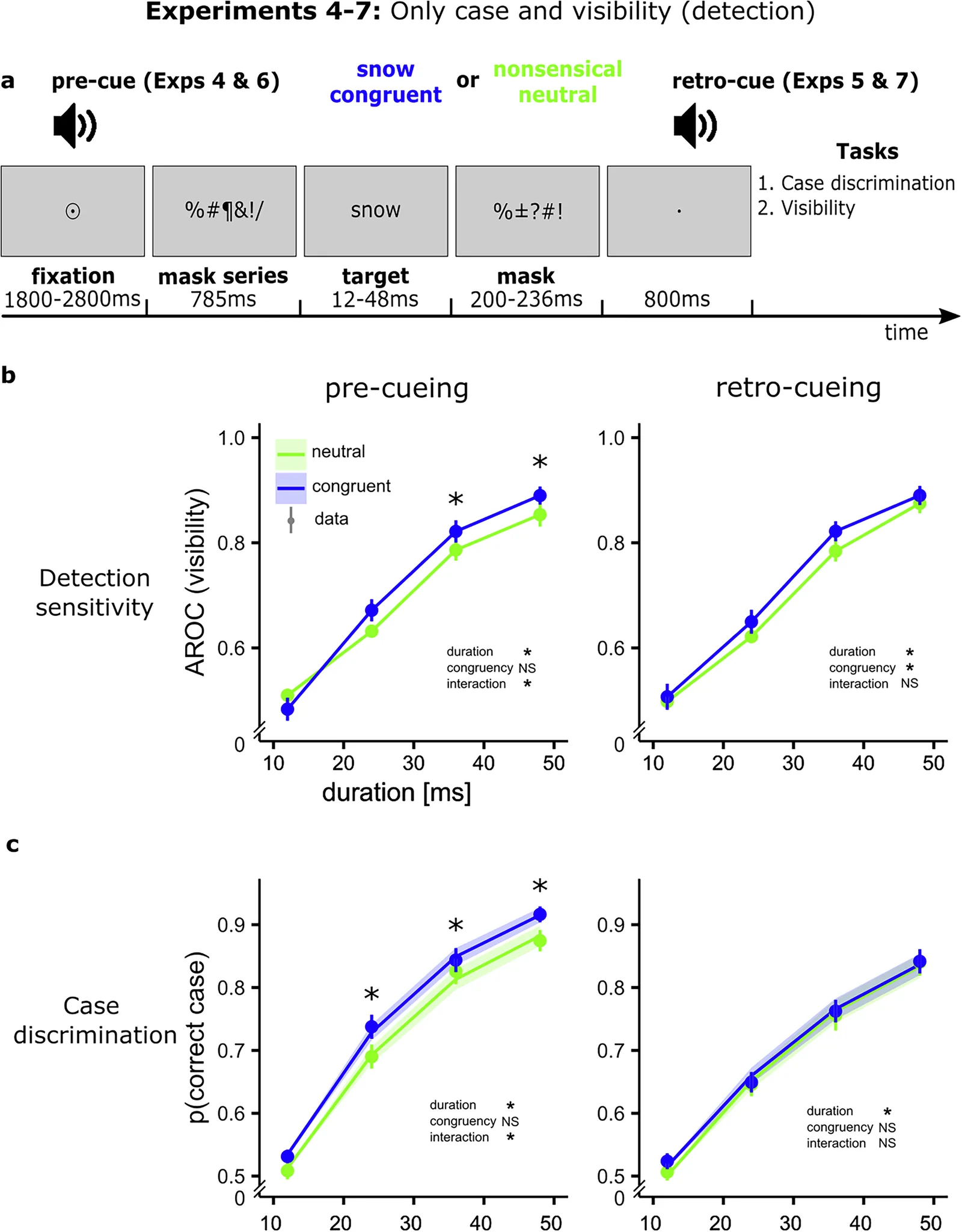
Experiments 4–7. **a** Participants were presented with one stream of centered stimuli. In experiments 4 and 6, a pre-cue was heard before the masks-target-mask sequence with a SOA of 1600 ms. In experiments 5 and 7, a retro-cue was heard after the sequence with a SOA of 248 ms. Response screens asked for case discrimination and overall visibility rating, in this order. Detection sensitivity (**b**) and case discrimination performance (**c**) are shown for pre-cueing (left panels) and retro-cueing (right panels). Points correspond to the data; lines represent performance estimated by the model. Error bars are the standard error (SE) of the mean across observers (*n *= 36), and error bands are the SE of model predictions across observers (*n* = 36). Deep blue items correspond to congruent trials, and green items to neutral trials. Stars indicate intervals where post-hoc comparisons were significant or panels where the statistical analysis showed a significant effect of congruency with no significant post-hoc test.

Eighteen participants were included in each of these 4 experiments (see **Methods** and Fig. 5a). We took advantage of these experiments to control for additional factors from our initial design. First, to ensure that the observed improvements in performance were due to a positive effect of congruent cues and not, or not only, due to a negative effect of incongruent ones (priming the incorrect lexicon could be thought to decrease performance), we replaced incongruent cues by neutral non-words that were either the reversed audio track of congruent cues (4 and 5) or pure tones with the same duration and envelope as the congruent cue (6 and 7). We also changed cueing from associative (“porcupine” – hedgehog) to repetition (“hedgehog” – hedgehog) in all Experiments. Crucially, the task was also simplified: participants did not have to report the word identity (which became trivial when the cue repeated the word), they only had to report letter-casing and visibility of the whole stimulus. This maximized our chance to observe cueing effects at the level of visual representations, since participants could focus all their attention on the visual attributes of the target. Neutral cues were changed between Experiments 4&5 and 6&7 because participants sometimes reported being able to recover the original word from the reversed audio tracks on neutral trials, hence bringing neutral trials closer to congruent trials. The patterns of results were similar for both pairs of experiments (see Supplementary Fig. 6). In what follows the two pre-cueing experiments (4 and 6) and the two retro-cueing experiments (5 and 7) were pooled for analysis.

Case discrimination was analyzed using separate logistic regressions for pre-cueing and retro-cueing experiments, using target duration and congruency as factors. These analyses were followed by a third logistic regression model that included the type of cue (pre or retro-cue) as a factor, alongside congruency and target duration. One-sided tests were used, given that our previous experiments allowed strong priors about the directions of the expected effects (positive effect of congruent cues vs incongruent ones, positive effect of increasing target duration, negative effect of retro-cueing vs pre-cueing). Results of both experiments are presented in Fig. 5.

For detection sensitivity, the patterns obtained matched our predictions as both pre and retro-cueing influenced detection (Fig. 5b). For pre-cueing, detection sensitivity significantly increased with target duration (F(1.89,66.00) = 236.59, *p* < 0.001, η²G = 0.62, 95% CI for η²_G_ [0.50, 0.70]). There was no evidence for a statistically significant effect of congruency with a marginal *p*-value (F(1,35) = 3.34, *p* = 0.08, η²G = 0.009, 95% CI for η²_G_ [0.00, 0.07]) but there was a significant interaction between duration and congruency (F(3,105) = 9.37), *p* < 0.001, η²G = 0.01, 95% CI for η²_G_ [0.00, 0.06]). Further one-sided post-hoc pairwise comparisons showed an increase with congruent pre-cues for target durations 36 and 48 ms (Supplementary Note 2). For retro-cueing, we found a significant main effect of target duration on detection sensitivity (F(1.54,54.06) = 202.96, *p* < 0.001, η²G = 0.64, 95% CI for η²_G_ [0.52 0.71]), a significant main effect of congruency (F(1,35) = 5.83, *p* = 0.02, η²G = 0.01, 95% CI for η²_G_ [0.00, 0.08]), and no evidence of a statistically significant interaction between duration and congruency (F(1.54,54.06) = 1.70, *p* = 0.1, η²G = 0.003, 95% CI for η²_G_ [0.00 0.007]).

For case discrimination (Fig. 5c), analyses conducted separately for the two types of cues showed that congruency improved case discrimination for pre-cues as evidenced by a significant interaction with target duration (target duration 95%BCI-Odds Ratio [1.99, 2.42], congruency 95%BCI-Odds Ratio [0.80, 1.14], interaction 95%BCI-Odds Ratio [1.04, 1.23]), but for retro-cues we found no significant effect of congruency and no interaction with duration (target duration 95%BCI-Odds Ratio [1.73, 2.13], congruency 95%BCI-Odds Ratio [0.94, 1.30], interaction 95%BCI-Odds Ratio [0.90, 1.04]). Data were then analyzed using the full model (congruency x target duration x type of cue) which revealed a main effect of target duration (95%BCI-Odds Ratio [2.00, 2.47]), an interaction between congruency and target duration (95%BCI-Odds Ratio [1.02, 1.19]), target duration and type of cue (95%BCI-Odds Ratio [0.74, 0.99]), and, crucially, a triple interaction between target duration, congruency and type of cue (95%BCI-Odds Ratio [1.02, 1.20]). No main effect of congruency (95%BCI-Odds Ratio [0.84 1.19]) or type of cue (95%BCI-Odds Ratio [0.91 1.35]), or interaction between congruency and type of cue (95%BCI-Odds Ratio [0.86, 1.39]) was found.

Overall, this pattern of results suggests that, while both congruent pre-cues and retro-cues improved detection, only pre-cues, not retro-cues, improved case discrimination, although participants could focus all their attention on this visual attribute since they did not have to report the word’s identity.

## Discussion

In this study, we probed a counter-intuitive prediction, derived from the Global Neuronal Workspace Theory, that it should be possible to trigger conscious access to high-level representations of a stimulus even after the associated low-level sensory representations have been masked. We conducted a first series of three experiments which all replicated the same observations: over a range of several durations of a masked visual word, on the rising part of the psychometric function, participants’ ability to report the identity of the word was substantially improved when it was followed by a semantically related auditory word (congruent retro-cue) compared to an unrelated word (incongruent retro-cue). The range of durations where this improvement occurred matched the typical durations where unconscious semantic processing is known to take place^32,35^. This improvement was accompanied by increased objective ability to detect the presence of the target, and increased subjective visibility, showing that the effect did not rely on subliminal processing but rather on the fact that retro-cues retrospectively improved conscious access. In contrast, no improvement was observed for reporting lower-level features such as letter casing (Experiments 1 and 3) or position on screen (Experiment 2), although semantic cues also allow improved expectations at the level of the visual form of the word. Further analyses confirmed that congruent retro-cues induced a dissociation between the ability to identify the word and to report its visual features. Experiments 5 and 7 replicated the observation that retro-cues induced increased detection sensitivity and visibility, but had no effect on case discrimination.

Although these results verified our initial predictions, we had to consider another possible interpretation. Congruent retro-cues could have improved performance not by retrospectively improving awareness but by providing additional information on trials where participants already had some partial awareness of the masked word^55–57^. For example, an initial glimpse of the letters “edg”, combined with the retro-cue “porcupine”, could allow one to infer “hedgehog”. We controlled for this at three different levels. First, if better identification occurs on trials where people saw a few letters, congruent retro-cues should increase the association between the ability to report the word’s visual features and its identity, but here we observed the opposite effect (Figs. 2e, 3f, 4f). Second, we controlled for a more subtle version where partial awareness would concern abstract, non-visual, orthographic features. Contrary to what would be predicted in this case, retro-cueing did not influence the orthographic similarity between the target and the words produced by participants on error trials (Supplementary Fig. 3). Finally, we preregistered and performed two series of two experiments each to test the importance of temporal order between masking and cueing. Under our initial hypothesis, performance dissociation should critically depend on cue-target order, while it should not matter under the partial awareness hypothesis, which assumes that better performance only arises from combining the cue with partial information about the target. The results validated our predictions that pre and retro-cues impact performance differently (Fig. 5): while pre-cues improved both detection and case discrimination, retro-cues deployed on the same protocol improved participants’ detection without improving case discrimination, replicating our previous observations again. These controls hence emphasize that the dissociation between detection and case discrimination specifically arises when the cue is placed after the target and its masks, validating our proposition that the impact of retro-cues on conscious detection occurs after low-level features of the target have been degraded by masking.

Several previous studies have shown that a retrospective context word can influence the lexical processing or identification of a preceding masked word^58–63^. Here, we further investigated its impact on conscious access by using two benchmark measures^64^: objective detection sensitivity (or d’) and subjective visibility. Our results showed that improved identification with retro-cueing was not akin to a form of “blindsight”^65,66^, but that it was instead genuinely associated with a change in participants’ conscious experience, as reflected by increased subjective visibility and objective detection sensitivity.

We further attempted to probe whether participants were able to distinguish over which dimension, high-level or low-level, their subjective visibility increased. We obtained mixed results: in Experiment 2, an effect of retro-cue congruency could be observed on subjective visibility and derived detection sensitivity for both dimensions, although the effect was a bit weaker for the low-level dimension (Fig. 3d,e). One interpretation could be that improved detection of the word meaning was accompanied by an illusion of having seen also lower features of the stimulus despite no effect on objective performance; however, this result could also be due to how instructions were delivered. In Experiment 3, we hence replaced visibility for confidence; this time, each scale was clearly associated with judging confidence over either the identification response or the case response. We observed a dissociation in confidence, matching the dissociation observed on first-level performance (Fig. 4d,e). There was still a slight effect of congruency for the longest duration for confidence in the low-level response: on the confidence distributions, this corresponded to a transfer of trials with high confidence to trials with maximal confidence, in contrast with the effect observed for word identification, where we observe a transfer of responses from low and mid-level confidence to maximal confidence (Supplementary Fig. 5). Overall, this suggests that participants are generally willing to express better perception of the stimulus when using a visibility scale, especially given partially shared instructions across the different scales, but that they probably do not experience an illusion of seeing the low-level feature, as the effect of congruency on this dimension only concerns a modulation of confidence on trials that were already confident.

### Limitations

Although we were able to conduct a large number of experiments to test our hypotheses in detail, our study suffers from several limitations. We aimed at investigating the subjective experiences associated with retro-perception by using ratings for visibility and confidence on different features of a single stimulus. Our choice of instructions for visibility ratings in Experiment 2 was coarse and may not have allowed individuals to distinguish between features: indeed, both scales shared the instruction to use visibility values bigger than zero as soon as they had a feeling that they might have detected something. Future work will be needed to better characterize participants’ subjective experience at each level independently. As highlighted in studies on sound-induced flash illusions, metacognitive awareness can be much more subtle and difficult to reveal than first-order effects on behavior, especially in rapid multisensory integration situations^67,68^. In our case, null effects for subjective visibility could be specifically reassessed in a dedicated protocol. We provided instructions with a focus on distinguishing between the presence and absence of the stimulus, which may not have been suited to explore subtle differences in the perceptual quality of experiences of word-meaning and word-form.

Another major limitation of the present study is that, although we could make sure that partial awareness could not account for the cueing effect we observed, it is also clear that partial awareness did occur across all conditions, in the form of catching a glimpse of a few letters and using this information to respond. This limitation is inherent to our protocol: a balance must be struck between the intensity of visual masking and the unmasking potential of the cue. Indeed, semantic processing of masked words is highly sensitive to masking strength^35^ and decays rapidly over time^31,34^. It would, however, be possible to circumvent this limitation, as masking is not the only method for producing unconscious stimuli: for example, diverting attention away from the target by using the attentional blink^69^ could shed a complementary light on the results presented here.

These results bring important evidence for current debates about the role of sensory processing in conscious perception. They seem to challenge theories postulating that conscious perception is linked with the establishment of local loops within sensory cortices^10,12^, since visual masking typically disrupts these loops, and hence should definitively preclude conscious perception thereafter^30,35,70^. More precisely, the local recurrency hypothesis proposes that we can become phenomenally conscious when local recurrency happens, and that reportability is optional, comes on top of this phenomenal awareness, and is supported by longer-range loops. According to this model, there can be phenomenal consciousness in the absence of access if local recurrency occurs in the absence of long-range connectivity (a proposition that is disputed, see Cohen and Dennett^71^). But conversely, no conscious perception, neither phenomenal nor access, should be possible in the absence of local recurrency. Since masking disrupts local recurrency and we still observe improved conscious access (improved detection sensitivity) with our retrospective cue, it suggests that awareness of the presence of the word can occur even when local recurrencies at the visual level have been disrupted. This observation constitutes a challenge to the local recurrency proposition, although it is not definitive proof.

These results appear to validate a radical prediction of contender theories, and corroborate the neurophysiological mechanism proposed in Fig. 1. We know from previous literature that the processing of a visual word can reach lexical or semantic analysis before masking disrupts the associated low-level representations^35,72,73^. Although these high-level representations can prime subsequent conscious words, they typically remain unconscious. Here, we propose that retrospective cueing triggers conscious access to these initially unconscious high-level representations via the following mechanism: first, the retro-cue induces a spreading of activations in lexical and/or semantic networks^36^; if the unconscious high-level representations left by the past visual word belong to the same lexical field, this should reactivate them^74–77^. These reactivated representations, being relevant to the task, might be selected by attention and broadcast within a global workspace^9,17^, allowing for conscious access despite the degradation of the associated visual details, several hundred milliseconds after stimulus disappearance.

Although these results seem to contradict classical formulations of local loop theories, which typically focus on the construction of sensory representations within the first levels of the hierarchy –that are disrupted by pattern masking^4,70,78^, we could still interrogate whether an extended formulation of this local loops framework could, in hindsight, accommodate our results. One could argue that retro-cues act by retrospectively triggering local recurrent processing in higher-level areas such as temporal visual areas. We would need to assume that, although our masks only disrupt recurrent processing at low levels, they nevertheless manage to affect recurrency also at higher levels, and that retro-cues allow to establish these initially failed local recurrencies. The role of local recurrencies in the build-up of sensory representations is well established for low-level sensory attributes (typically, a figure-ground context modulates the encoding of primary visual attributes via these recurrencies)^4,70,78^, and it is this type of phenomenon that has grounded the Recurrent Processing Theory. It is less clear what type of additional local recurrencies would be required at the lexical or semantic level for allowing conscious perception of representations at this level only, knowing that unconscious lexical or semantic representations of the masked word exist in the first place, independently of retro-cueing. So, while it remains possible to interpret these results within an extended formulation of the local loops framework, such an interpretation would be less straightforward.

Importantly, over and beyond arbitrating between different models, these results highlight the flexibility of conscious access mechanisms with regard to the initial stages of sensory build-up, an observation that any model of conscious perception should take into account. They corroborate the previously described retro-perception phenomenon, namely the possibility to trigger conscious perception retrospectively^25,26,28,29,39,79^, and extends it to visual masking. In doing so it shows that conscious access is not only flexible in time but also in type of content. Indeed, present results reveal a phenomenon of dissociated conscious percepts, where participants report having seen a stimulus that they correctly identify, despite being almost clueless about its visual details. This suggests that the mechanisms for conscious access are relatively insensitive to the “completeness” of an experience, so that they can operate on higher-order representations in the absence of a precise sensory base. This is in line with the theoretical proposal made in the Partial Awareness hypothesis^55^ which suggests not only that there can be access to fractions of letters during masking, but more generally that higher and lower levels of representation of the same stimulus can potentially be accessed independently. Here we further show that this can happen retrospectively, after the disappearance of the stimulus, and that even the capacity to detect the presence of a visual word can be dissociated from the ability to report its visual features.

These results also resonate with the Reverse Hierarchy Theory^80^, which proposes that implicit feedforward processes trigger the appropriate receptive fields in higher-level areas for fast and effortless gist-perception (here, word meaning), and that conscious perception of details (here, letter casing or location) may be optional and requires focused reactivation of low-level areas from the top. In Reverse Hierarchy terms, the present results would suggest that retro-cues induce top-of-the-hierarchy conscious experiences followed by a downward stream of detail enhancement, which fails when it reaches levels affected by masking.

Along a similar line, these results also extend the idea of a “predictive brain”^81^. There is now ample evidence that what we will perceive next, and even *whether* we will perceive it consciously or not^82^ is strongly influenced by the past, and by a projection of what can be expected next based on that past. For example, Lupyan & Ward show that semantic prediction influences whether a stimulus crosses the consciousness threshold or not^82^. Here, we show that combining information to get more than instantaneous evidence is a process that can go in both directions: the predictive brain is also a retrodictive brain.

In conclusion, these findings are highly relevant to current debates over the role of sensory processing in consciousness. They demonstrate the extreme flexibility of the form that can be taken by conscious perception and conscious access to an external stimulus, both in terms of timing and content. They suggest that conscious perception of a word can be reduced to its most essential property, its meaning: we know that a stimulus has been presented; we know what it is; we agree to say that we have “seen it”, but we don’t know what it looks like. This brings support to theories of consciousness for which the processes of becoming conscious of a representation can be distinguished from the processes underlying its elaboration, suggesting a wide range of ways to experience any single event.

This is reminiscent of the large inter-individual variations in how a representation can be experienced, for example, in aphantasia, an absence or near-absence of mental imagery, which concerns about 2.5% of the population^83^. These kinds of phenomena are all the more interesting in that they challenge our intuitions about conscious perception and its relationship with conscious experience in general. More generally, these results challenge our understanding of the relationship between conscious experience and external events, and interrogate the very nature of what we call a conscious percept. They also open perspectives for renewing the way we explore the neural underpinnings of conscious processing.

## Supplementary information

Transparent Peer Review file

Supplementary Information PDF

## Data Availability

Data and scripts are available at https://osf.io/kd2wh/, https://osf.io/t6hxb and https://osf.io/sqcpm as well as from the corresponding author upon reasonable request.
