# Supplementary material for: Consciously detecting and recognizing a past visual word after its sensory trace is gone
Source: Commun Psychol. 2026 Jun 9;4:137. doi: 10.1038/s44271-026-00478-9 (PMC13658779; doi:10.1038/s44271-026-00478-9)
Supplement: Supplementary file 1 [file 44271_2026_478_MOESM1_ESM.pdf]

nature portfolio

Peer Review File

Consciously detecting and recognizing a past visual word after its sensory trace is gone

Corresponding Author: Dr Daphne Rimsky Robert

This file contains all editorial decision letters in order by version, followed by all author rebuttals in order by version.

Version 0:

Decision Letter:

Dear Dr Rimsky-Robert,

Thank you for your patience during the peer-review process. Your manuscript titled "Consciously detecting and recognizing a past visual word after its sensory trace is gone" has now been seen by 3 reviewers, whose comments are appended below. I have discussed the reports with my colleagues and I regret to inform you that we decided that in light of the referee reports, we cannot publish your manuscript in Communications Psychology.

The reviewers express enthusiasm about the research question and the potential novel insights. However, you will see that they also raise substantive concerns, in particular with regard to the strength evidence put forward to support your conclusions. Taking these points together with our editorial considerations, these reservations preclude publication of the current version of this manuscript in Communications Psychology.

We would be open to reconsidering this decision should you be able to collect substantive amounts of new data, which convincingly address the concerns regarding the perception of the stimuli even at shortest durations, the way that guessing contaminate the results and how the Bayesian regression accounts for it, and the suitability of the design to address your research questions. We consider these requests beyond what the journal can ask for in revisions.

I am sorry that we cannot be more positive on this occasion and thank you for the opportunity to consider your work.

Best regards,

Mael Lebreton

Mael Lebreton, PhD
Editorial Board Member
Communications Psychology
orcid.org/0000-0002-2071-4890

REVIEWERS' EXPERTISE:
Reviewer #1: conscious perception & masking protocols, Bayesian and logistic models
Reviewer #2: conscious perception & semantic priming
Reviewer #3: consciousness, masking, semantic priming

REVIEWER COMMENTS:

Reviewer #1 (Remarks to the Author):

This study presents five experiments that investigate whether retrospective cueing can improve conscious access to semantic information without access to the lower level sensory information. The experiments extend earlier results showing that retrospective attentional cueing can induce an improved conscious perception of a low-level visual stimulus (Gabor patches). The results are highly similar across experiment and show that retrospective cueing improves access to

unconscious semantical information without improving access to low level perceptual information.

This is an interesting manuscript with experiments that test an interesting hypothesis to arbitrate between different theories of consciousness. While the paper is nicely written and the experimental design is easy to understand, the methods (models) used to analyze the data are not. The multivariate models that are construed do seem to be logic, but how can we now if these are accurate models with respect to the observed data? Is there any way to calculate a fit of the model to the data? This makes it difficult to judge the – very consistent - results across experiments and the validity of the conclusions. Some of my comments below are related to this (described in relation to the first experiment but applicable to all of them). I describe some minor and less minor remarks in more details below.

- Line 167: it says that the strength of masking is varied between trials but this is not entirely correct as the mask remains identical across trials. The stimulus strength varies across trial.
- Line 171: Aren't catch trials added to see if there is a bias in the participants' responses rather than their sensitivity?
- Lines 217-219: The significant interaction effect in word identification is interpreted as if there is a decrease in the congruency effect when stimulus duration increases. However, the congruency effect in the shortest target duration can hardly be said to be the largest (numerically nor statistically).
- The results from case discrimination do not show an interaction effect but they do show a main effect of target duration. From the figure(s) it looks as if the proportion of correct responses is above chance level (i.e., 0.5) even for the shortest target durations. Doesn't this indicate some awareness of the targets? Also in Figure 2 of the supplementary material does the visibility rating of the shortest duration trials seem to be higher than the catch trials, suggesting some visibility for this condition. Furthermore, only about 50% of the 12ms trials have a rating of 0 (all ratings > 0 suggest some visibility).
- The interpretation of the results on detection sensitivity is confusing to me (Figure 2d). Why would congruency be relevant here? Isn't the crucial comparison here whether or not the ROC is different from the diagonal, i.e. whether or not the area under the curve is different from 0.5? And if congruency would be the relevant factor here, because it is informative with respect to the semantical information, shouldn't it be a significant factor for the shortest durations too?

Reviewer #2 (Remarks to the Author):

Summary
The objective of the paper was to test the Global Workspace Theory (GWT) and recurrent Processing Theory (RPT) against each other. To do that the authors tested the effects of a retro-cue (a spoken word) on conscious perception of a masked written word. They suggested that the mask should obliterate the low-level perceptual representations of the word (e.g., its letter case and location) but not its semantic representation. According to the authors, GWT predicts that the retro-cue should boost conscious access of semantic but not of lower-level perceptual representations. They further argue that this should not be possible according to RPT. In 3 experiments, they presented a masked word (target present) or a blank (target absent), and then a retro-cue consisting of an auditory word that was either a close semantic associate of the target or an unrelated word. A perceptual property of the target was also manipulated (letter case in E1 and E3 and location in E2). To evaluate conscious access of the semantic and low-level perceptual information, the authors mainly relied on naming (identification) of the word and forced-choice discrimination of the low-level property, respectively. In addition, they measured the visibility of the word on a multi-point scale in E1, the visibility of the word identity and of its location, on separate scales, in E2, and confidence in the identification/discrimination response separately for the word identity and its case in E3. E4 and E5 were control experiments in which the effects of the retro-cue were compared to the effects of a pre-cue. The results showed that in all experiments the retro-cue improved identification of the word but not discrimination of its low-level properties. However, the retro-cue also improved the visibility of the low-level properties, a finding that the authors do not dwell on. The results of the control experiments show that pre-cues were not particularly successful in boosting conscious access to case information. Nevertheless, the authors concluded that overall, the results supported their hypothesis.
Evaluation
This study presents an interesting extension of previous findings using retro-cues: the attempt to show that retro-cues selectively enhance conscious access of the representations that are not obliterated by masks provides a provocative test of the idea that the timing of conscious access can be decoupled from the timing of sensory stimulation, with conscious access resulting from offline reactivation of trailing representations. However, several aspects of this work limit my enthusiasm. From a theoretical viewpoint, I am not sure that the present study is a good test of the GWT against RPT. The retro-cue can be viewed as boosting recurrent processing and thereby as rescuing from unawareness representations of the word that are not erased by the mask (its semantic representation) – an outcome that would be in line with RPT. More importantly, I don't think that the present data provide strong support for the author's hypothesis, for several reasons.
1. In E1, the only outcome on which the authors rely to claim that there was as a dissociation between conscious perception of the word's meaning vs. its case is the fact that word discrimination is boosted by cue-word congruency, whereas case discrimination is not. However, several additional aspects of this experiment are worth considering. First, mean visibility and detection sensitivity, which the authors take to also index conscious perception of the word's meaning, show a pattern that seems to be dissociated from word discrimination performance: while for the former, the effect of congruence increased with duration, for the latter, the effect of congruence decreased with duration. The authors do not address this dissociation, yet it undermines the idea that all three measures index the same process (access to awareness). Second and relatedly, for the 12-ms duration, visibility was null (i.e., participants reported not seeing anything at all on virtually all trials) and congruence had no effect on visibility (Supplementary Figure 2), yet the authors report a significant congruence effect on word identification. Two accounts for this pattern of results come to mind: (1) word identification accuracy indexes subliminal perception rather than conscious perception in this experiment, hence the dissociations between visibility and word identification accuracy. (2) the procedure that the authors used to correct for guesses (which was rather technical despite the simplicity of the underlying logic) was not successful at entirely eliminating the influence of guesses; this interpretation is in

line with the finding that both the impact of congruence and the impact of guessing on word identification performance decreased with target duration (whereas the impact of congruency on visibility increased with target duration). Such guessing of course, could not occur for case discrimination, hence the dissociation.
2. Note that the argument that word-identification performance on congruent trials may have been contaminated by guessing based on the retro-cue (rather than based on the trailing semantic representation) can also explain the response consistency results: response consistency was higher for incongruent trials (in which participants relied only on visual processing of the target for both decisions) than for congruent trials (in which they relied also on guessing for semantic decision and only on visual information for the low-level decision).
3. In E2, the authors claim that they show a dissociation between the effect of the retro-cue on conscious perception of semantic vs. position information ("In conclusion, Experiment 2 …successfully extends the dissociation between awareness of abstract features versus physical features to the spatial location of the stimulus" p.16)., but the data seem to show otherwise. Again, the authors rely on the fact that congruence improves word identification performance but not localization performance. However, (1) again, the impact of congruence as a function of duration is different for word identification performance vs. for detection sensitivity and visibility of the word's identity, (2) detection sensitivity data (as well as visibility data in the Supplementary materials) clearly show a strikingly similar effect of congruence for word and location detection sensitivity/visibility. Therefore, the data provide evidence quite at odds with the authors conclusions. And again, the subliminal perception and residual guessing accounts can explain the data.
4. The rationale for E3 is not clearly stated. From what I could understand, the authors aimed at explaining why the retro-cue improved location visibility in E2. However, the predictions were vague: "Here we wanted to test whether such increased visibility and detection in the absence of increased objective performance on the tested visual feature corresponded to an illusion of being able to discriminate this visual feature… and assessed whether, in contrast with visibility ratings, confidence ratings would reflect the dissociation observed in first-order performance". Since congruence did affect confidence for the case discrimination for some durations, I am not sure what the authors' conclusion is. The data show that the congruent cue increased participants' confidence for the semantic decision (which could result from the fact that the cue helped them guess). Congruence also improved confidence for the case decision (with the 48-ms duration for confidence sensitivity and 36-ms and 48-ms durations for mean confidence rating), for which there is little reason to expect that the retro-cue could give participants the illusion of seeing the case better. So, it seems that the data actually go precisely against the authors' claims. However, since the authors did not clearly explain their rationale, I may have missed the point.
5. The control experiments with pre-cuing show that attention does not benefit case discrimination much: there was an interaction between congruence and duration but for no duration was the effect of congruence significant. Finding a clear effect with precues was essential for validating the authors' hypothesis that precues would facilitate conscious perception of both low-level and semantic information. In fact, the task used in the control experiment should have yielded stronger effects than would be expected in the previous experiments because (a) it required fewer responses from the participants and (b) it induced them to focus exclusively on case. Yet, the results showed very weak trends in the expected direction.
6. Even if we assume that the congruence effect on word identification performance was not contaminated by subliminal perception (i.e., that it did not boost unconscious processing) nor by guessing, it is not clear that the retro-cue affected access to conscious perception rather than improving faint conscious perception. The authors attempted to invalidate the idea that there was partial awareness of the stimuli in fairly indirect ways (response consistency analyses). However, the data clearly show that in all experiments, the effect of congruence on word visibility / confidence occurred only for durations at which the incongruent word was perceived above catch-trial level. The relevant evidence would be to show that for a duration at which incongruent words are at catch-trial level, congruent words are above that level.
7. The authors performed sophisticated analyses to assess position discrimination on correct identification trials for retro-perceived words and claimed that such performance was at chance. In E1, it stood at about 70%: the fact that it was not significant only hints that the measure may be very noisy or that the study was underpowered for this analysis. In E2, it seems to be significant – yet the authors do not discuss these findings.
8. I think the ms would gain from adding some clarity and precision to the predictions for each measure, and to the discussion of the findings. Each experiment ends with a conclusion that simply states that the predictions were supported, when in fact there are many reasons to question that it was indeed the case. An example of this is the contrast between the conclusion of E2 and the intro of E3: the authors ignore the problem of congruency effects on location visibility in the former and hint to it in the intro to E3 – this is confusing and misleading, I think. The authors should come to grips more explicitly with the discrepancies between their measures and with alternative accounts for their data.
9. It would be useful to add an intermediate level of explanation for the important aspects of the methods. The procedures used to correct for guessing and to control for partial awareness were pretty technical and their description in the main text was overly simplified. The reader should get a better sense of what was done, before diving into the technical details presented in the methods section.
10. The sample size seems small, and I could not see a clear justification of it.

To summarize, although the question addressed in this study should be of great interest to the field, the data do not strongly support the authors' claims: (1) guessing and/or subliminal perception can account for the main finding; (2) the subjective-awareness and objective-performance data are inconsistent with each other, and this inconsistency is not discussed; (3) the pre-cue results are very weak and do not suffice to justify the authors' conclusions and (4) the study seems to be underpowered and (5) overall, the authors oversell their claims and do not attempt to cope with the problems associated with the pattern of results they report.

Minor comments
- The visibility measure in E1 was ambiguous: it is not clear whether participants had to report their experience of the word's meaning – as intended by the authors - or of the physical attributes of the word. This measure was improved in E2, but contrary to what is written in the main manuscript (that one visibility measure targeted only the word's location and the other the word's meaning), supplementary note 1 suggests that the former measure tapped both the word's location and its

meaning: indeed, the 4 questions were similar for the identity and location judgments except for levels 5-8. I did not understand this choice. If there was a rationale behind this decision it would be useful to spell it out. In this regard, why not use a variant of PAS (the Perceptual Awareness Scale), which have been validated by many studies?
- In Figure 1, it would be useful to add that the semantic representation is not erased.
- The auditory cue lasted less than 800ms – how long did it last? Why were there 6 premasks? How long was the recording delay?

Reviewer #3 (Remarks to the Author):

Rimsky-Robert et al. elegantly used a retrospective cueing paradigm to examine the prediction that consciousness can be independent of local signal build-up due to the recurrent loops in the sensory system, which is put forth by the global workspace theory. In five experiments, the authors convincingly showed that a retrospective semantically congruent cue boosted the identification of an earlier word, but not the discrimination of low-level visual characteristics, such as case (experiment 1), location (experiment 2). Experiment 3 further showed no increased confidence associated with congruent cues. Finally, experiments 4 and 5 clearly showed that the dissociation between the semantic and local visual feature rescue occurred only to the retro-cues but not pre-cues, suggesting that different mechanisms are involved. In general, I find the manuscript well written, the analyses well executed, and the results are convincing. Still, I have some questions that I hope the authors can address to further strengthen their work.

Major comments

1. I like how the authors used experiment 3 to address the possibility that the increased visibility and detection can be conflated with confidence. Although the results largely refuted this idea, I have to point out that the metacognitive differences in such an ultra-fast identification task could be subtle and hard to detect. For example, a recent study showed higher confidence in an audio-visual illusion (sound-induced flash illusion) when the two modalities were matched (Mayes et al., 2023). This metacognitive component was not particularly considered in the earlier work and was assessed when a variation of the illusion was introduced (Stiles et al., 2018). However, no confidence difference was found in the illusory and non-illusory conditions in this study, which could possibly be attributed to the limited sample size. That is, most studies are equipped with strong enough statistical power to detect the main phenomenon while the same participant pool may not be sufficient to further detect a more nuanced, metacognitive difference. I would like to see this addressed in the context of the current study.

Maynes, R., Faulkner, R., Callahan, G., Mims, C. E., Ranjan, S., Stalzer, J., & Odegaard, B. (2023). Metacognitive awareness in the sound-induced flash illusion. Philosophical Transactions of the Royal Society B, 378(1886), 20220347.

Stiles, N. R., Li, M., Levitan, C. A., Kamitani, Y., & Shimojo, S. (2018). What you saw is what you will hear: Two new illusions with audiovisual postdictive effects. PloS one, 13(10), e0204217.

2. I find the results consistent with an earlier account: reverse hierarchy proposed by Hochstein & Ahissar (2002). In this account, they proposed that conscious perception begins at the end (top) of the sensory system, which allows quick access to gist-like, abstract, or higher-level forms of visual representation. While the more detailed representations come later after scrutiny. This is consistent with the current findings where conscious identification can be rescued by a semantically congruent retro-cue, even when the lower-level features are erased. I find a more thorough discussion on this separation of low-level and high-level sensory signals and their respective interaction with conscious access will greatly improve the impact of the current article.

Hochstein, S., & Ahissar, M. (2002). View from the top: Hierarchies and reverse hierarchies in the visual system. Neuron, 36(5), 791-804.

Minor comment

1. The stars in Figure 5 indicate the congruency effect as illustrated in the legend. However, visually it can be confusing since they do not follow the same duration-specific rules as previous figures. I suggest drawing the stars between the two colored lines with an arrow or pointer.

Shao-Min Hung

Note on appeals: In exceptional circumstances, it is in authors' interest to appeal an editorial decision. More information on appeals is available here: https://www.nature.com/commspsychol/submit/resources#appeals

Version 1:

Decision Letter:

** Please ensure you delete the link to your author homepage in this e-mail if you wish to forward it to your coauthors **

Dear Dr Rimsky Robert,

Thank you for your patience during the peer-review process. Your manuscript titled "Consciously detecting and recognizing a past visual word after its sensory trace is gone" has now been seen by 3 reviewers, and I include their comments at the end of this message. They find your work of interest but raised some important points. We are interested in the possibility of publishing your study in Communications Psychology, but would like to consider your responses to these concerns and assess a revised manuscript before we make a final decision on publication.

We therefore invite you to revise and resubmit your manuscript, along with a point-by-point response to the reviewers. Please highlight all changes in the manuscript text file.

Editorially, we consider important that you address the remaining limitations highlighted by Reviewer #2, who questions the validity and interpretation of your control conditions. Considering the feedback from Reviewers 1 and 2, you should ensure that the theoretical scope of your findings, assumptions, and interpretations does not extend beyond what your approach and data reasonably support.

As you revise the manuscript in response to these issues, please also implement all requests in the attached Mandatory Revision Requests document. All requirements listed in this document need to be fully met, or the work will be returned to you for further revisions without peer review. This workflow is in place to increase the likelihood that the paper will be accepted for publication. It reduces the number of rounds of revision (and review) and ensures that the reviewers vet a version of the article that is compliant with journal policies. If you have any questions regarding the required revisions, please contact the journal prior to resubmission to avoid a negative outcome.

We are committed to providing a fair and constructive peer-review process. Please don't hesitate to contact us if you wish to discuss the revision in more detail.

Please submit the following items:

- Revised manuscript
- Point-by-point response to the referees' comments
- Mandatory Revision Requests Table (attached).
- Cover letter (as a separate document)

via this link: Link Redacted .

** This url links to your confidential home page and associated information about manuscripts you may have submitted or are reviewing for us. If you wish to forward this email to co-authors, please delete the link to your homepage first **

We hope to receive your revised paper within 8 weeks; please let us know if you aren't able to submit it within this time so that we can discuss how best to proceed. If we don't hear from you, and the revision process takes significantly longer, we may close your file. In this event, we will still be happy to reconsider your paper at a later date, provided it still presents a significant contribution to the literature at that stage.

We look forward to seeing the revised manuscript and thank you for the opportunity to review your work.

Best regards,

Mael Lebreton

Mael Lebreton, PhD
Editorial Board Member
Communications Psychology
orcid.org/0000-0002-2071-4890

REVIEWER EXPERTISE:
Reviewer #1:conscious perception, masking protocols
Reviewer #2:conscious perception, semantic priming
Reviewer #3:conscious perception, masking protocols

REVIEWER REPORTS:

Reviewer #1 (Remarks to the Author):

This revised manuscript addresses all of my comments. It presents a nice series of experiments with interesting and very results that replicate well across experiments. Although the theoretical scope of the findings and some of assumptions in the analysis of the data might still be up for discussion, the manuscript presents them in a convincing way. I think many readers will find this an fascinating study and I therefore fully endorse its publication.

Reviewer #2 (Remarks to the Author):

I was a reviewer on the previous version of this paper. I appreciate that the authors clarified many of the imprecisions that made understanding the previous version somewhat challenging. However, lit seems to me that these clarifications raised more concerns than they resolved.
1. I will start with the control experiments. Although the authors explained their motivation for using repetition cues instead of associative cues, it seems to me that this change created a problem: specifically, participants face a very different situation in the pre-cue and retro-cue conditions. In the former, when the pre-cue is a word, participants can focus exclusively on the case of the target because they already know the identity of the word, and all they will have to do is repeat it - whereas when the pre-cue is neutral, they have to divide their attention between the word meaning and its case, hence the observed "congruence" effect. By contrast, with the retro-cue, since participants do not know in advance whether or not the cue will be a word, they have to divide their attention in both conditions, hence the absence of an effect.
2. Relatedly, it is not clear in the first place why one would expect a semantic cue to retrieve / enhance the case of the target. The authors did not flesh out the argument. The results of the control experiments conveyed the impression that, somehow, the cue did increase case-discrimination performance when it was a pre-cue, and I confess that the demonstration initially convinced me - but as I explained above, it seems to me that the finding arose from a confound. I apologize for not spotting this problem in the earlier version.
3. The authors refer to a dissociation between word detection/identification and case discrimination, but I still cannot figure out what the dissociation is. If I understood correctly, the authors rely on two findings: (a) the congruence effects occur for meaning and not for case – but see my above question about why they should and the alternative account of the control experiments' results; (b) On the fraction of trials where targets were retro-perceived, case discrimination was not at ceiling – but performance was rather high (around 70%) and when it was at chance, the CI was huge.
4. Figure 1 suggests that sensory features are erased whereas semantic ones are preserved but there is no evidence for that: it is entirely possible that the sensory feature traces exist and remain unconscious because the cue is not useful for retrieving / enhancing them and bring them to consciousness. Alternatively, they could survive the mask but decay faster (a bit in the same vein as in Posner's classical 1969 paper on the time course of AA vs. Aa representations).
5. I found the description of the recurrent processing theory as a "local loops" framework misleading: in Lamme's theory, local loops support phenomenal awareness whereas longer-range loops support report - which is what the authors measured here. I still think that the findings cannot disentangle the two models.

6. In short, I think that what the study shows is that different aspects of a stimulus can be selectively brought to conscious perception by retro-cues: here, the target's meaning by a cue that is useful to rescue it from unconsciousness (a finding that is in line with the framework suggested by Kouider et al., 2010). In passing, I think that this paper is relevant here not only because of the partial awareness argument but also for its theoretical claims (namely, their hypothesis that "representations at each level can be accessed independently from each other" (p. 304).

Dominique Lamy

References

Kouider, S., De Gardelle, V., Sackur, J., & Dupoux, E. (2010). How rich is consciousness? The partial awareness hypothesis. Trends in cognitive sciences, 14(7), 301-307.

Posner, M. I., Boies, S. J., Eichelman, W. H., & Taylor, R. L. (1969). Retention of visual and name codes of single letters. Journal of experimental psychology, 79(1p2), 1.

Reviewer #3 (Remarks to the Author):

The authors have addressed my previous comments adequately. I am happy to see this study added to the literature.

* TRANSPARENT PEER REVIEW: Communications Psychology uses a transparent peer review system. This means that we publish the editorial decision letters including Reviewers' comments to the authors and the author rebuttal letters online as a supplementary peer review file. However, on author request, confidential information and data can be removed from the published reviewer reports and rebuttal letters prior to publication. If your manuscript has been previously reviewed at another journal, those Reviewers' comments would not form part of the published peer review file.

** Visit Nature Research's author and referees' website at www.nature.com/authors for information about policies, services and author benefits**

Communications Psychology is committed to improving transparency in authorship. As part of our efforts in this direction, we are now requesting that all authors identified as ‘corresponding author’ create and link their Open Researcher and Contributor Identifier (ORCID) with their account on the Manuscript Tracking System prior to acceptance. ORCID helps the scientific community achieve unambiguous attribution of all scholarly contributions. You can create and link your ORCID from the home page of the Manuscript Tracking System by clicking on ‘Modify my Springer Nature account’ and following the instructions in the link below. Please also inform all co-authors that they can add their ORCIDs to their accounts and that they must do so prior to acceptance.
https://www.springernature.com/gp/researchers/orcid/orcid-for-nature-research

For more information please visit http://www.springernature.com/orcid

If you experience problems in linking your ORCID, please contact the Platform Support Helpdesk.

Version 2:

Decision Letter:

** Please ensure you delete the link to your author homepage in this e-mail if you wish to forward it to your coauthors **

Dear Dr Rimsky Robert,

Your manuscript titled "Consciously detecting and recognizing a past visual word after its sensory trace is gone" has now been seen by our reviewers, whose comments appear below. In light of their advice I am delighted to say that we are happy, in principle, to publish a suitably revised version in Communications Psychology.

We therefore invite you to revise your paper one last time to address the remaining concerns of our reviewers and a list of editorial requests. At the same time we ask that you edit your manuscript to comply with our format requirements and to maximise the accessibility and therefore the impact of your work.

EDITORIAL REQUESTS:

Please review our specific editorial comments and requests regarding your manuscript in the attached "Editorial Requests Table". Please outline your response to each request in the right hand column. Please upload the completed table with your manuscript files as a Related Manuscript file.

If you have any questions or concerns about any of our requests, please do not hesitate to contact me.

SUBMISSION INFORMATION:

In order to accept your paper, we require the files listed here https://www.nature.com/documents/commsj-file-checklist.pdf .

OPEN ACCESS:

Communications Psychology is a fully open access journal. Articles are made freely accessible on publication. For further information about article processing charges, open access funding, and advice and support from Nature Research, please visit https://www.nature.com/commspsychol/open-access

At acceptance, you will be provided with instructions for completing the open access licence agreement on behalf of all authors. This grants us the necessary permissions to publish your paper. Additionally, you will be asked to declare that all required third party permissions have been obtained, and to provide billing information in order to pay the article-processing charge (APC).

* TRANSPARENT PEER REVIEW: Communications Psychology uses a transparent peer review system. On author

request, confidential information and data can be removed from the published reviewer reports and rebuttal letters prior to publication. If you are concerned about the release of confidential data, please let us know specifically what information you would like to have removed. Please note that we cannot incorporate redactions for any other reasons.

* CODE AVAILABILITY: All Communications Psychology manuscripts must include a section titled "Code Availability" at the end of the methods section. We require that the custom analysis code supporting your conclusions is made available in a publicly accessible repository at this stage; please choose a repository that generates a digital object identifier (DOI) for the code; the link to the repository and the DOI must be included in the Code Availability statement. Publication as Supplementary Information will not suffice.

* DATA AVAILABILITY:
All Communications Psychology manuscripts must include a section titled "Data Availability" at the end of the Methods section. More information on this policy, is available in the Editorial Requests Table and at http://www.nature.com/authors/policies/data/data-availability-statements-data-citations.pdf.

Please use the following link to submit the above items:
Link Redacted
** This url links to your confidential home page and associated information about manuscripts you may have submitted or be reviewing for us. If you wish to forward this email to co-authors, please delete the link to your homepage first **

We hope to hear from you within two weeks; please let us know if you need more time.

Best regards,

Jennifer Bellingtier

Jennifer Bellingtier, PhD
Senior Editor
Communications Psychology

Mael Lebreton, PhD
Editorial Board Member
Communications Psychology
orcid.org/0000-0002-2071-4890

REVIEWER EXPERTISE:
Reviewer #2:conscious perception, semantic priming

REVIEWERS' COMMENTS:

Reviewer #2 (Remarks to the Author):

The authors have addressed my comments to a level that does not require further revisions. I therefore recommend publication in the manuscript's present form.

** Visit Nature Research's author and referees' website at www.nature.com/authors for information about policies, services and author benefits**

Open Access This Peer Review File is licensed under a Creative Commons Attribution 4.0 International License, which permits use, sharing, adaptation, distribution and reproduction in any medium or format, as long as you give appropriate credit to the original author(s) and the source, provide a link to the Creative Commons license, and indicate if changes were made.
In cases where reviewers are anonymous, credit should be given to 'Anonymous Referee' and the source.
The images or other third party material in this Peer Review File are included in the article's Creative Commons license, unless indicated otherwise in a credit line to the material. If material is not included in the article's Creative Commons license and your intended use is not permitted by statutory regulation or exceeds the permitted use, you will need to obtain permission directly from the copyright holder.
To view a copy of this license, visit https://creativecommons.org/licenses/by/4.0/

Response to reviewers

Our responses are written in blue, and references to the changes we applied in the new version of the manuscript are highlighted in yellow.

As a general note:
during the course of this revision, we not only corrected our model (see details in the responses) but we also verified all analyses and rewrote the main scripts in R rather than Matlab for better accessibility of the dataset on the OSF. This led to the correction of a few typos and marginal variations in ANOVAs F values and bootstrapped confidence intervals reported in the text. There was no change in significance of these tests.

REVIEWER COMMENTS:

Reviewer #1 (Remarks to the Author):

This study presents five experiments that investigate whether retrospective cueing can improve conscious access to semantic information without access to the lower level sensory information. The experiments extend earlier results showing that retrospective attentional cueing can induce an improved conscious perception of a low-level visual stimulus (Gabor patches). The results are highly similar across experiment and show that retrospective cueing improves access to unconscious semantical information without improving access to low level perceptual information.

This is an interesting manuscript with experiments that test an interesting hypothesis to arbitrate between different theories of consciousness. While the paper is nicely written and the experimental design is easy to understand, the methods (models) used to analyze the data are not. The multivariate models that are construed do seem to be logic, but how can we now if these are accurate models with respect to the observed data? Is there any way to calculate a fit of the model to the data? This makes it difficult to judge the – very consistent - results across experiments and the validity of the conclusions. Some of my comments below are related to this (described in relation to the first experiment but applicable to all of them). I describe some minor and less minor remarks in more details below.

We thank the reviewer for their attentive reading and their positive remarks. We actually agree with the main concern expressed here, regarding the accuracy of our model, and we are grateful for this remark as it has incited us to revise our model and propose a much better and much simpler one. The logic of the new model is the same, but we get rid of a boundary problem that made the previous one inaccurate.

More specifically: in order to correct for the guesses in identification performance for congruent cues (namely: reporting an associated word that happens to be the target just by chance, without having any information from the target) we have to estimate their proportion from the incongruent condition, in which these guesses are directly identifiable since they result in an error. Subtracting these "incongruent guesses" from performances in the congruent condition (for each participant and each duration) is very efficient and makes the datapoints for congruent and incongruent conditions overlap for the shortest target duration of 12ms (corrected datapoints are now directly shown in the figures for more clarity, see an example in the figure below on the right panel: the points are the corrected datapoints for experiment 1).

The logic of our initial model was the same as this direct correction, however it is clear that it did not correct as well, as shown in the figure below on the left, which reproduces the figure of the previous version of the manuscript for experiment 1: estimated performance for congruent condition is always higher than for incongruent condition, even for 12ms targets.

While re-working on the manuscript, we realized that this imperfect fit of the model could be due to a boundary effect. Indeed, the direct subtraction on the datapoints can sometimes bring corrected performance slightly below zero: when performance in congruent trials is the same as the rate of incongruent guesses, as appears to be the case for 12ms targets, both numbers actually

come from the same distribution so the subtraction can be negative. But in a logistic regression, estimates are strictly bounded at zero, and this might explain why the fit was off at the lowest durations.

In order to circumvent this problem, we built a new model in which we changed the order of the operations: we first brought the performance for the incongruent condition at a level that was comparable to the congruent condition by adding the incongruent guesses to the correct trials in the incongruent condition. We then fitted a new model, which was much simpler since we could then use the same logistic regression for both conditions (see new main text lines 232-243 and new Methods lines 1027-1069). This got rid of the boundary problem since guess rate at 12ms is quite high (around 25%, see Supplementary Figure 1). We then removed the estimates of the incongruent guesses from both curves to obtain the corrected word identification in both conditions. As shown below for experiment 1, this new procedure allowed a much better fit.

This new procedure has been applied to all the analyses of word identification, in experiments 1, 2 and 3, as shown in the new figures 2b, 3b and 4b, and corresponding text sections. This more robust analysis highlights that the effect is less strong in experiment 2 than in the other two experiments, but it is still clearly significant.

Finally, to further address the reviewer's remark that we should provide indicators of the validity of our model, we now include the diagnostics of all our fits in Supplementary table 3 and mention them in the Methods from line 1070.

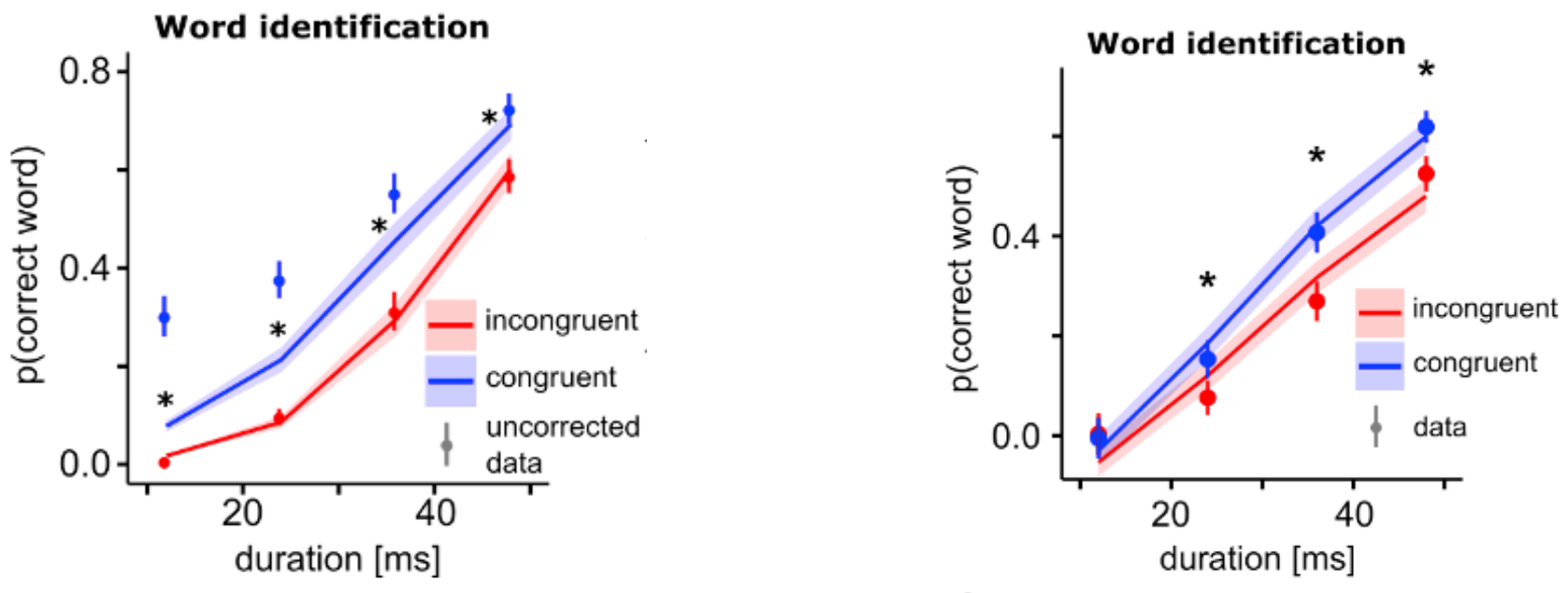

Word identification in experiment 1. Left: previous version of Figure 2b with raw datapoints (uncorrected) and estimates from the previous model. Right: current version of Figure 2b with corrected datapoints (direct correction with no modelling) and the estimates of the new model.

- Line 167: it says that the strength of masking is varied between trials but this is not entirely correct as the mask remains identical across trials. The stimulus strength varies across trial.

We have corrected that in the text line 186.

- Line 171: Aren't catch trials added to see if there is a bias in the participants' responses rather than their sensitivity?

Actually, catch trials allow to derive both detection bias and detection sensitivity, since they allow to calculate false alarms. This has been added to the text lines 189 192.

- Lines 217-219: The significant interaction effect in word identification is interpreted as if there is a decrease in the congruency effect when stimulus duration increases. However, the congruency effect in the shortest target duration can hardly be said to be the largest (numerically nor statistically).

This was indeed a distortion from our previous model. With the new model, the interaction shows that the effect of congruency does increase with stimulus duration, see new manuscript line 248:

> "Our model revealed […] a significant interaction between target duration and congruency, with the congruency effect increasing with increasing target duration (95%BCI-Odds Ratio [1.02 1.36])".

- The results from case discrimination do not show an interaction effect but they do show a main effect of target duration. From the figure(s) it looks as if the proportion of correct responses is above chance level (i.e., 0.5) even for the shortest target durations. Doesn't this indicate some awareness of the targets? Also in Figure 2 of the supplementary material does the visibility rating of the shortest duration trials seem to be higher than the catch trials, suggesting some visibility for this condition. Furthermore, only about 50% of the 12ms trials have a rating of 0 (all ratings > 0 suggest some visibility).

We made the comparison to chance level for case discrimination and detection sensitivity at the shortest target duration and it confirmed the reviewer's observation that both metrics are significantly above chance in experiment 1. This is probably due to the fact that, in experiment 1, participants came for a first session where we assessed whether they could perform the task on a different list of words (see Methods and Supplementary Note 4 on Participants' preselection procedure in Experiment 1). This first session also constituted a solid training which explains why they might have been slightly better than chance for case discrimination and detection even for very short targets in the test session. In all the other experiments, from 2 to 7, we had more efficient instructions and training phases, so participants underwent a shorter training and directly entered the test phase on the same session. We added a sentence in the text to explain it, paragraph 1C, lines 274-285.

Concerning visibility ratings (shown in Supplementary figures 2, 5 and 6), it is important to note that, even when the stimulus is absent, not all visibilities are at zero, but other low visibilities are also used whereby participants express that they might have had a glimpse of something. So, the important comparison to assess participant's detection sensitivity is the comparison between the distribution of visibilities for catch trials versus target present trials. Our sensitivity measure (AUC) is derived from this comparison. We updated Supplementary figures 2, 5 and 6 to show the distribution of visibility ratings also for target absent trials, and complemented the figure legend to explain this better.

- The interpretation of the results on detection sensitivity is confusing to me (Figure 2d). Why would congruency be relevant here? Isn't the crucial comparison here whether or not the ROC is different from the diagonal, i.e. whether or not the area under the curve is different from 0.5? And if congruency would be the relevant factor here, because it is informative with respect to the semantical information, shouldn't it be a significant factor for the shortest durations too?

Improvement in detection sensitivity by the congruent semantic retro-cues was actually one of our strong predictions. Indeed, we hypothesize that semantically congruent retro-cues might not only improve word identification performance, but that it would be associated with improved conscious access to this information. Such improved conscious access should translate in improved subjective visibility and improved objective ability to report the presence of the word (detection sensitivity), both being standard measures of conscious access (Sandberg et al., 2010; Sergent & Dehaene, 2004). We explain this point better in the new manuscript, in the introduction (lines 149-152) and in the discussion (lines 726-731).

As pointed out by the reviewer, we could also have observed an effect at the shortest duration, but as it turns out, even in experiment 1 where case discrimination is slightly above chance and participants probably do sometimes have a glimpse of the stimulus at this duration, sensory processing might still be too weak to reach the level where semantic information can be extracted. We know from the masked priming literature that lexical or semantic priming effects disappear for very short durations of the word, suggesting that for these too brief stimuli, unconscious processing is too weak to reach this level (Kouider & Dehaene, 2007). Hence, classical experiments showing unconscious semantic processing of masked words typically use target durations between 30 and 50ms, where they find the best compromise allowing semantic processing to occur while conscious perception is still hindered (Dehaene et al., 1998; Kouider & Dehaene, 2007). Our present results are perfectly in line with this classical literature: we find retro-cueing effects mostly for target durations of 36 and 48ms, durations for which sensory processing of the target can reach lexical and semantic levels. We used four different target durations, and not

just one duration within the 30-50ms range, as is usually done in studies on unconscious semantic priming, in order to assess more precisely the range for which a retro-cueing effect might emerge. We explain this point better in the new manuscript, lines 125-135 and then in the discussion lines 723-728

Reviewer #2 (Remarks to the Author):

Summary

The objective of the paper was to test the Global Workspace Theory (GWT) and recurrent Processing Theory (RPT) against each other. To do that the authors tested the effects of a retro-cue (a spoken word) on conscious perception of a masked written word. They suggested that the mask should obliterate the low-level perceptual representations of the word (e.g., its letter case and location) but not its semantic representation. According to the authors, GWT predicts that the retro-cue should boost conscious access of semantic but not of lower-level perceptual representations. They further argue that this should not be possible according to RPT. In 3 experiments, they presented a masked word (target present) or a blank (target absent), and then a retro-cue consisting of an auditory word that was either a close semantic associate of the target or an unrelated word. A perceptual property of the target was also manipulated (letter case in E1 and E3 and location in E2). To evaluate conscious access of the semantic and low-level perceptual information, the authors mainly relied on naming (identification) of the word and forced-choice discrimination of the low-level property, respectively. In addition, they measured the visibility of the word on a multi-point scale in E1, the visibility of the word identity and of its location, on separate scales, in E2, and confidence in the identification/discrimination response separately for the word identity and its case in E3. E4 and E5 were control experiments in which the effects of the retro-cue were compared to the effects of a pre-cue. The results showed that in all experiments the retro-cue improved identification of the word but not discrimination of its low-level properties. However, the retro-cue also improved the visibility of the low-level properties, a finding that the authors do not dwell on. The results of the control experiments show that pre-cues were not particularly successful in boosting conscious access to case information. Nevertheless, the authors concluded that overall, the results supported their hypothesis.

Evaluation

This study presents an interesting extension of previous findings using retro-cues: the attempt to show that retro-cues selectively enhance conscious access of the representations that are not obliterated by masks provides a provocative test of the idea that the timing of conscious access can be decoupled from the timing of sensory stimulation, with conscious access resulting from offline reactivation of trailing representations. However, several aspects of this work limit my enthusiasm.

From a theoretical viewpoint, I am not sure that the present study is a good test of the GWT against RPT. The retro-cue can be viewed as boosting recurrent processing and thereby as rescuing from unawareness representations of the word that are not erased by the mask (its semantic representation) – an outcome that would be in line with RPT. More importantly, I don't think that the present data provide strong support for the author's hypothesis, for several reasons.

We thank the reviewer for their very careful reading and evaluation of the manuscript. We surmise from their comments that the reviewer found the topic and the study interesting, but considered that our results did not entirely support our conclusions.

We understand and acknowledge these criticisms; as detailed below, we have tackled them on several fronts: (1) we have corrected the model we used for word identification performance, which leads to more robust results that are also more coherent with the other measures, (2) where the reviewer highlighted a possible lack of power, we conducted additional preregistered studies

which backed up our initial results (3) we also rewrote the manuscript to take into account the reviewer's remarks and better explain the rationale of our experimental manipulations, better present our predictions, and better justify our conclusions.

The reviewer also noted as a general remark that the present study might not be a good test of the GWT against RPT, as the retro-cue could be viewed as boosting recurrent processing. This is a possibility which we now acknowledge in the discussion section, alongside a more developed argumentation for why we, nevertheless, think that post-sensory models of conscious perception such as the GWT provide a much more straightforward account of these results than theories that link conscious perception with local sensory processing (lines 822-840).

1. In E1, the only outcome on which the authors rely to claim that there was as a dissociation between conscious perception of the word's meaning vs. its case is the fact that word discrimination is boosted by cue-word congruency, whereas case discrimination is not. However, several additional aspects of this experiment are worth considering. First, mean visibility and detection sensitivity, which the authors take to also index conscious perception of the word's meaning, show a pattern that seems to be dissociated from word discrimination performance: while for the former, the effect of congruence increased with duration, for the latter, the effect of congruence decreased with duration. The authors do not address this dissociation, yet it undermines the idea that all three measures index the same process (access to awareness). Second and relatedly, for the 12-ms duration, visibility was null (i.e., participants reported not seeing anything at all on virtually all trials) and congruence had no effect on visibility (Supplementary Figure 2), yet the authors report a significant congruence effect on word identification. Two accounts for this pattern of results come to mind: (1) word identification accuracy indexes subliminal perception rather than conscious perception in this experiment, hence the dissociations between visibility and word identification accuracy. (2) the procedure that the authors used to correct for guesses (which was rather technical despite the simplicity of the underlying logic) was not successful at entirely eliminating the influence of guesses; this interpretation is in line with the finding that both the impact of congruence and the impact of guessing on word identification performance decreased with target duration (whereas the impact of congruency on visibility increased with target duration). Such guessing of course, could not occur for case discrimination, hence the dissociation.

We agree that these points were problematic in the previous version of the article. We should have acknowledged them. Possibly the reason why we did not focus on these incoherencies (the fact that congruency effect seemed to decrease with duration for word identification, contrary to visibility or detection sensitivity, and the significant effect of congruency even for the shortest duration) was that they were not present when we performed the correction of guesses directly on the data, without the intermediate of a model. Obviously, it was also our mistake that we did not show these more direct data in the previous version of the manuscript. Thanks to these remarks, we realized that there was a problem with our model and we corrected it; we also now show the results of the direct correction. The logic of the new model is the same, but we get rid of a boundary problem that made the previous one inaccurate, as explained in details below.

In order to correct for the guesses in identification performance for congruent cues (namely: reporting an associated word that happens to be the target just by chance, without having any information from the target) we have to estimate their proportion from the incongruent condition, in which these guesses are directly identifiable since they result in an error. Subtracting these "incongruent guesses" from performances in the congruent condition (for each participant and each duration) is very efficient and makes the datapoints for congruent and incongruent conditions overlap for the shortest target duration of 12ms (corrected datapoints are now directly shown in the figures for more clarity, see an example below on the right panel: the points are the corrected datapoints for experiment 1). The logic of our initial model was the same as this direct correction, however it is clear that it did not correct as well, as shown in the figure below on the left, which reproduces the figure of the previous version of the manuscript for experiment 1: estimated performance for congruent condition is always higher than for incongruent condition, even for 12ms targets, which the reviewer accurately noticed.

We realized that this imperfect fit of the model could be due to a boundary effect. Indeed, the direct subtraction on the datapoints can sometimes bring corrected performance slightly below zero: when performance in congruent trials is the same as the rate of incongruent guesses, as is the case for 12ms targets, sometimes the subtraction is slightly below zero, since both numbers actually come from the same distribution. But in a logistic regression, estimates are strictly bounded at zero, and this might explain why the fit was off at the lowest durations. In order to circumvent this problem, we built a new model in which **we changed the order of the operations**: we first brought the performance for the incongruent condition at a level that was comparable to the congruent condition by adding the incongruent guesses to the correct trials in the incongruent condition. We then fitted a new model, which was much simpler since we could then use the same logistic regression for both conditions (see new Methods lines 1027-1069). This got rid of the boundary problem since guess rate at 12ms is quite high (around 25%, see Supplementary Figure 1). We then removed the estimates of the incongruent guesses from both curves to obtain the corrected word identification in both conditions. As shown below for experiment 1, this new procedure allowed a much better fit.

This new procedure has been applied to all the analyses of word identification, in experiments 1, 2 and 3, as shown in the new figures 2b, 3b and 4b, and corresponding text sections. Finally, we now include the diagnostics of all our fits in supplementary table 3 and mention them in the Methods lines 1070-1077.

These more robust analyses show that the patterns of results are indeed coherent between word identification, visibility and detection sensitivity, and confirm the dissociation with case discrimination. This constitutes a very important improvement to the manuscript, and we are very grateful to the reviewers for raising these issues.

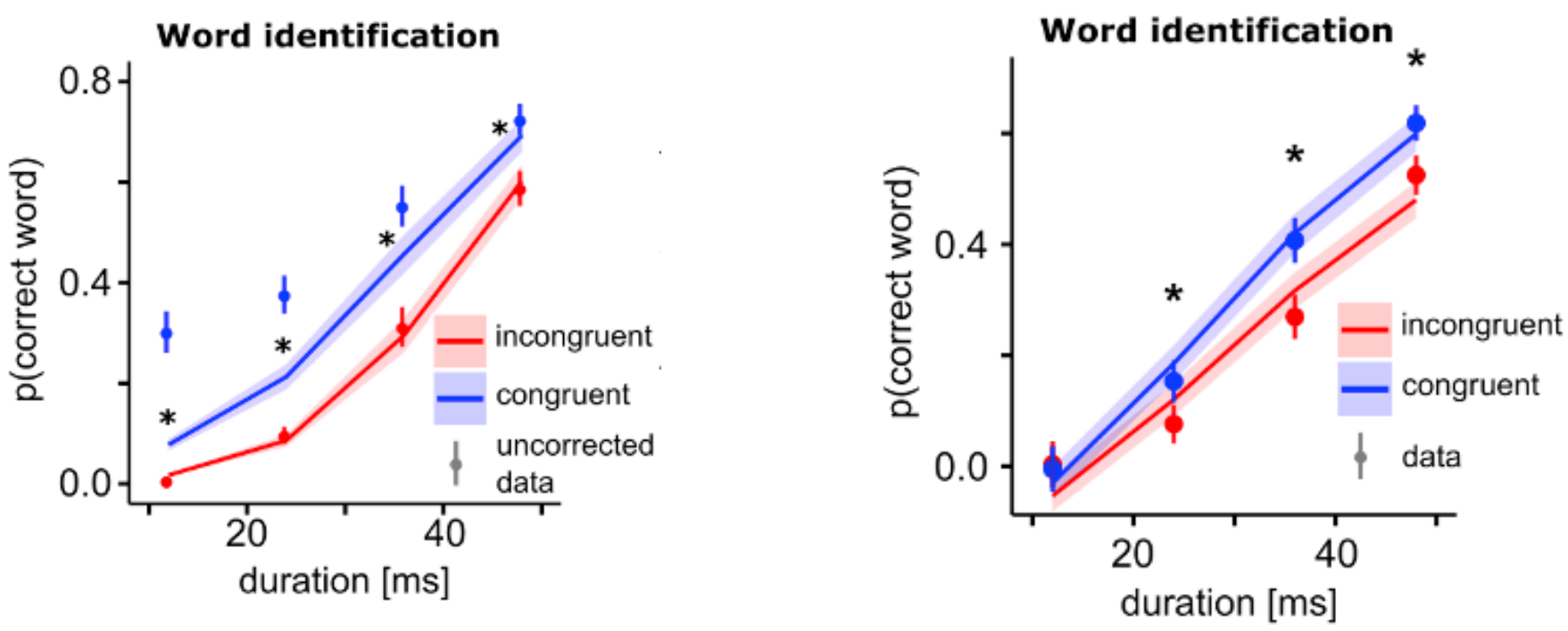

Word identification in experiment 1. Left: previous version of Figure 2b with raw datapoints (uncorrected) and estimates from the previous model. Right: current version of Figure 2b with corrected datapoints (direct correction with no modelling) and the estimates of the new model.

2. Note that the argument that word-identification performance on congruent trials may have been contaminated by guessing based on the retro-cue (rather than based on the trailing semantic representation) can also explain the response consistency results: response consistency was higher for incongruent trials (in which participants relied only on visual processing of the target for both decisions) than for congruent trials (in which they relied also on guessing for semantic decision and only on visual information for the low-level decision).

The new model does not allow to derive the response consistency anymore. But we still have the other analysis that assesses potential dissociations between word identification and case discrimination (or position discrimination) performance which consists in assessing case or position discrimination in word correct trials. In this analysis, we corrected for identification guesses in congruent trials on an individual trial basis, taking advantage of the trial-by-trial visibility rating: we calculated an individual, per target duration, visibility cut-off below which a correct trial could safely be classified as guess (since the response was correct but the subjective rating was low). If a participant's point-estimate of the guess rate (based on the guesses identified in the incongruent

condition) was n%, we excluded all trials with visibility equal or below that of the nth percentile. This cut-off was most of the time between visibility 0 and visibility 1. This procedure is explained in Methods, lines 1090-1101 of the revised manuscript. We observed that, following this correction for guesses, case (or position) discrimination performance was significantly lower for congruent than incongruent in experiments 1, 2 and 3 (Figure 2.e, Figure 3.f and Figure 4.f), suggesting that it is not the guesses that induce this dissociation.

3. In E2, the authors claim that they show a dissociation between the effect of the retro-cue on conscious perception of semantic vs. position information ("In conclusion, Experiment 2 …successfully extends the dissociation between awareness of abstract features versus physical features to the spatial location of the stimulus" p.16)., but the data seem to show otherwise. Again, the authors rely on the fact that congruence improves word identification performance but not localization performance. However, (1) again, the impact of congruence as a function of duration is different for word identification performance vs. for detection sensitivity and visibility of the word's identity, (2) detection sensitivity data (as well as visibility data in the Supplementary materials) clearly show a strikingly similar effect of congruence for word and location detection sensitivity/visibility. Therefore, the data provide evidence quite at odds with the authors conclusions. And again, the subliminal perception and residual guessing accounts can explain the data.

In response to point (1): as in experiments 1 and 3, in experiment 2 these incoherencies have disappeared in the new version of the article where we applied a direct correction on the data themselves, and got rid of the bias in our model (see above).

As for point (2): we acknowledge that the interpretation of the results for visibility and detection sensitivity for words versus position is not very straightforward. We were aiming at testing whether participants could distinguish over which dimension their subjective visibility increased. It is true that one possible prediction is that participant's subjective visibility should only be affected on the semantic dimension, not the low-level dimension. However, we were also interested in another possibility: that they might have an illusion to see the low-level feature when they "feel" they detected the stimulus. Hence, this manipulation was exploratory, as both predictions were plausible. And the results we obtained were intermediate: there was an effect of increased subjective visibility and increased detection sensitivity on both dimensions, but less strong on the low-level dimension (position).

In the new manuscript, we state these predictions explicitly in the introduction to E2 (line 397-413), then relate the results to these predictions (line 456-464), and then in the discussion section we provide a more thorough and careful, discussion of these results, in relation to results on confidence in E3 (lines 771 to 794)

4. The rationale for E3 is not clearly stated. From what I could understand, the authors aimed at explaining why the retro-cue improved location visibility in E2. However, the predictions were vague: "Here we wanted to test whether such increased visibility and detection in the absence of increased objective performance on the tested visual feature corresponded to an illusion of being able to discriminate this visual feature… and assessed whether, in contrast with visibility ratings, confidence ratings would reflect the dissociation observed in first-order performance". Since congruence did affect confidence for the case discrimination for some durations, I am not sure what the authors' conclusion is. The data show that the congruent cue increased participants' confidence for the semantic decision (which could result from the fact that the cue helped them guess). Congruence also improved confidence for the case decision (with the 48-ms duration for confidence sensitivity and 36-ms and 48-ms durations for mean confidence rating), for which there is little reason to expect that the retro-cue could give participants the illusion of seeing the case better. So, it seems that the data actually go precisely against the authors' claims. However, since the authors did not clearly explain their rationale, I may have missed the point.

Clarifying the predictions and conclusions of the visibility results in E2, as done in response to the previous question, also helps clarifying the rationale for E3 which we now fully develop in the introduction to this study (line 516 to 529):

> "We designed Experiment 3 to provide an additional replication of our initial results (Experiment 1) and probe a possible distinction between subjective visibility ratings and confidence ratings. Indeed, in Experiment 2, detection sensitivity, as measured with visibility ratings, increased similarly with congruent retro-cues for both visual (position) and more abstract (semantic) dimensions of the stimulus, contrasting with the dissociated effects of congruency on word identification versus position discrimination. This could have two interpretations: either improved perception of the word identity with retro-cues is accompanied by an illusion of better perceiving its visual features, or the partially shared instructions for both visibility ratings induced this similarity in responses. Here we address this issue by changing visibility scales for confidence scales, with each scale being dedicated to judging confidence over either the identification response or the case response. If the similar patterns obtained for visibility ratings was due to overlapping instructions, these well-separated confidence ratings should instead reflect the dissociation observed in first-order performance."

This time there was a clear contrast between confidence sensitivity for high level versus low level information (Figure 4 d and e), despite a slight congruency effect for 48ms targets for the low-level dimension highlighted by the reviewer. The difference between the patterns of results obtained on the two dimensions was significant (lines 567-575), contrasting with an absence of significant difference in the previous experiment. This suggests that participants are not deluded concerning their performance on the low-level dimension when asked about their confidence. As for the effect at 48ms for low-level dimension, a closer look at the distributions of confidence ratings suggests that it relates to an increase in confidence for ratings that were already confident (from confidence around 6 to confidence around 8), not a change from no confidence to high confidence, which contrasts with the distributions observed for word confidence (Supplementary Figure 6). We now develop these points better in the results description and conclusion line 582-589, and again in the discussion lines 771 to 794.

5. The control experiments with pre-cuing show that attention does not benefit case discrimination much: there was an interaction between congruence and duration but for no duration was the effect of congruence significant. Finding a clear effect with precues was essential for validating the authors' hypothesis that precues would facilitate conscious perception of both low-level and semantic information. In fact, the task used in the control experiment should have yielded stronger effects than would be expected in the previous experiments because (a) it required fewer responses from the participants and (b) it induced them to focus exclusively on case. Yet, the results showed very weak trends in the expected direction.

It is important to emphasize that, although the effects of pre-cueing on case discrimination were modest, they were significant, in stark contrast with all the other four experiments with retro-cues in which we did not find any significant effect of cueing (either main effect or interaction with duration) on case discrimination or position discrimination.

However, we acknowledge that the effect sizes in these experiments were small. Hence, following this remark, and in order to further alleviate doubts on whether this might be a false positive, we decided to consolidate this result by performing a preregistered replication of these two experiments (one with pre-cues, one with retro-cues), now included in the new manuscript as Experiments 6 and 7 (lines 627 to 709, Figure 5 and Supplementary Figure 7). Figure 5 shows the results merged from these two pairs of experiments, hence including 36 participants for pre-cues and 36 participants for retro-cues. This confirmed the previous pattern of results; notably post-hoc t-tests now show significant effects of pre-cueing on case discrimination for several durations, while no effect of congruency was found for retro-cues on case discrimination. We performed a statistical comparison between the patterns of results obtained for pre-cueing and retro-cueing on case discrimination, which confirmed their difference with a significant triple interaction between type of cue, congruency and duration (lines 691-699).

Finally, regarding the reviewer's other remark: it is true that asking participants to focus exclusively on case was expected to increase the effect size, at least on case discrimination; however, the use of a neutral condition instead of an incongruent condition was expected to have

the opposite effect, so that in sum it was reasonable to expect effect sizes in the same range as other effects observed in the study.

6. Even if we assume that the congruence effect on word identification performance was not contaminated by subliminal perception (i.e., that it did not boost unconscious processing) nor by guessing, it is not clear that the retro-cue affected access to conscious perception rather than improving faint conscious perception. The authors attempted to invalidate the idea that there was partial awareness of the stimuli in fairly indirect ways (response consistency analyses). However, the data clearly show that in all experiments, the effect of congruence on word visibility / confidence occurred only for durations at which the incongruent word was perceived above catch-trial level. The relevant evidence would be to show that for a duration at which incongruent words are at catch-trial level, congruent words are above that level.

It is important to highlight that a semantic retro-cueing effect is only expected to occur if semantic processing of the target word has happened in the first place, as illustrated in Figure1. We know from the masked priming literature that lexical or semantic priming effects disappear for very short durations of the word, suggesting that for these too brief stimuli, unconscious processing is too weak to reach this level of processing (Kouider & Dehaene, 2007). Hence, classical experiments showing unconscious semantic processing of masked words typically use target durations between 30 and 50ms, where they find the best compromise allowing semantic processing to occur while conscious perception is still hindered (Dehaene et al., 1998; Kouider & Dehaene, 2007). The present results are perfectly in line with this classical literature: we find retro-cueing effects mostly for target durations of 36 and 48ms, durations for which sensory processing of the target can reach lexical and semantic levels. We explain this point better in the new manuscript, lines 125-133.

It is true that there can be partial awareness of the stimuli at these durations, but we did not attempt to invalidate this idea; on the contrary we directly measured this partial awareness using the orthographic distance between erroneous responses and the target word, see Supplementary Figure 4. This is a very direct measure of partial awareness as it assesses to what extent words given by the participants when they missed the target nevertheless resembled the target word orthographically, matching the definition of partial awareness (Kouider & Dupoux, 2004). We show that this orthographic distance decreases slightly but significantly with target duration, demonstrating that there is partial awareness at least for the three longest durations. But crucially we demonstrate that this partial awareness is not responsible for the retro-cueing effect that we observe, since it is unaffected by the congruency of the retro-cue (Supplementary Figure 4). As we explain in the text (paragraphs 1E, 2E and 3E), if improved word identification and detection with congruent retro-cues was due to words that were already partially seen, leading to the correct response when combined with the cue, then these words should disappear from the errors, hence increasing the average orthographic distance between the remaining erroneous responses and the targets. This is not what we observe, in neither of the three experiments where this measure could be applied. We believe that this provides a robust and direct demonstration that partial orthographic awareness is not responsible for the effect we observe.

The response consistency analyses could not be performed with this new corrected model. But we still provide another control by analyzing case (or position) discrimination in word-correct trials for congruent versus incongruent cues. The argument is also quite direct in our opinion: if the effect of retro-cues relied on trials where participants had a glimpse of the target, then their performance in reporting the case or position of the target should be particularly good on those trials, and hence we should expect performance for these low-level discriminations to be at least as good in word correct trials for congruent that for incongruent cues. But we observe the opposite: low-level discrimination significantly worsens on word-correct trials for congruent cues as compared to incongruent cues, in all three experiments where we could test it, suggesting that the congruency effect does not rely on trials where participants had some partial awareness of the low-level features of the stimulus in the first place. We reformulated this logic to make it clearer in the text, lines 308-321.

7. The authors performed sophisticated analyses to assess position discrimination on correct identification trials for retro-perceived words and claimed that such performance was at chance. In E1, it stood at about 70%: the fact that it was not significant only hints that the measure may be very noisy or that the study was underpowered for this analysis. In E2, it seems to be significant – yet the authors do not discuss these findings.

We acknowledge that the important result in these analyses is not the estimate of the performance for retro-perceived words, which is undoubtedly noisy, but the fact that we observe decreased performance for congruent trials compared to incongruent trials, which clearly argues against the idea that improved identification with congruent cues relies on trials where participants had awareness of the low-level features of the stimulus (see previous response). We thus amended the text to bring more caution to the interpretation of the estimate of low-level performance for retro-perceived words, and highlight the fact that it is only indicative (end of paragraphs 1D, 2D and 3D).

8. I think the ms would gain from adding some clarity and precision to the predictions for each measure, and to the discussion of the findings. Each experiment ends with a conclusion that simply states that the predictions were supported, when in fact there are many reasons to question that it was indeed the case. An example of this is the contrast between the conclusion of E2 and the intro of E3: the authors ignore the problem of congruency effects on location visibility in the former and hint to it in the intro to E3 – this is confusing and misleading, I think. The authors should come to grips more explicitly with the discrepancies between their measures and with alternative accounts for their data.

We fully understand this remark and the review has indeed helped us state our predictions and discuss our results more clearly and precisely, as developed in response to points 3 and 4 above.

9. It would be useful to add an intermediate level of explanation for the important aspects of the methods. The procedures used to correct for guessing and to control for partial awareness were pretty technical and their description in the main text was overly simplified. The reader should get a better sense of what was done, before diving into the technical details presented in the methods section.

In the new version of the manuscript, the model was greatly simplified. As explained in response to point 1, it now corresponds to a standard mixed-effect logistic regression.

For analysis of partial awareness, the most complex analysis rested on what we had called “response consistency”, which can no longer be derived from our regression parameters. For conditional probability analyses, we added clearer definitions of terms lines 1090-1101 in the Methods, and precisions in the main text lines 308-315. For Levensthein distance analysis, we added a description of the algorithm’s purpose in the main text lines 340-343.

10. The sample size seems small, and I could not see a clear justification of it.

The number of participants per experiment was constrained to 18 in order to ensure complete counterbalancing of stimulus presentation across participants. In the previous version of the manuscript, this constraint was already mentioned, but only in the methods section, and explained in details in the Supplementary Note 4. But following this remark we acknowledge that we should also provide justification for the sample size in the main text, which we now do in Line 174-180:

> “In each experiment, the number of participants was constrained to 18 in order to ensure complete counterbalancing of stimulus presentation across participants (explained in details in **Supplementary Note 3**). This is similar to the sample size of previous experiments testing other retrospective effects which, based on a power analysis, included around twenty participants (Garnier-Allain et al., 2023). The replicability of the effects was

tested across three different variants of the experiment (experiments 1 to 3), plus two sets of control experiments (experiments 4 to 7)."

We should also emphasize that Bayesian inference focuses on the precision of the posterior distribution rather than on asymptotic properties tied to large sample sizes. As now shown in supplementary table 3, which lists the diagnostics of our model fits, the effective sample size (n_eff) for the effects of congruency (main effect and interaction with duration) is between 1,800 and 3,500, far above commonly recommended thresholds (e.g., > 1,000 for stable posterior estimates). This indicates that the posterior estimates are precise and that inference is not limited by sampling uncertainty in the Markov Chain Monte Carlo procedure.

To summarize, although the question addressed in this study should be of great interest to the field, the data do not strongly support the authors' claims: (1) guessing and/or subliminal perception can account for the main finding; (2) the subjective-awareness and objective-performance data are inconsistent with each other, and this inconsistency is not discussed; (3) the pre-cue results are very weak and do not suffice to justify the authors' conclusions and (4) the study seems to be underpowered and (5) overall, the authors oversell their claims and do not attempt to cope with the problems associated with the pattern of results they report.

In summary, we replied to the reviewer's 5 points above in the following way:
(1) our corrected model now guarantees a total correction for guessing, as attested both on datapoints and model. We confirm that our effect cannot be due to guessing. Furthermore, if such performance increase was due to subliminal perception, by definition it should not reflect in better detection sensitivity and increased subjective visibility, a point that we now better emphasize in the manuscript (in the introduction lines 149-152, in the discussion lines 726-731).
(2) the correction of our model has rectified this inconsistency
(3) we have performed two new preregistered replications of our control experiments. They replicate our initial result and confirm that pre-cues enhance both detection sensitivity and case discrimination while retro-cues only improve detection sensitivity (Figure 5).
(4) we better explain our sample size and we provide diagnoses showing that our studies are not underpowered. Finally, following the addition of two new experiments, the article now includes 5 replications of our central observation.
(5) following this revision, the pattern of results is much more solid, but we have also used the reviewers' remarks to add more nuance in our interpretations and better acknowledge other possible interpretations in the discussion.

Minor comments

- The visibility measure in E1 was ambiguous: it is not clear whether participants had to report their experience of the word's meaning – as intended by the authors - or of the physical attributes of the word. This measure was improved in E2, but contrary to what is written in the main manuscript (that one visibility measure targeted only the word's location and the other the word's meaning), supplementary note 1 suggests that the former measure tapped both the word's location and its meaning: indeed, the 4 questions were similar for the identity and location judgments except for levels 5-8. I did not understand this choice. If there was a rationale behind this decision it would be useful to spell it out. In this regard, why not use a variant of PAS (the Perceptual Awareness Scale), which have been validated by many studies?

In E1 we used a global measure of visibility; we made no mention of differentiating between attributes, and if participants asked, we told them to "make a global assessment of visibility". This is in line with similar uses of a global visibility scale in numerous other experiments (Del Cul et al., 2007; Garnier-Allain et al., 2023; Sergent et al., 2013; Sergent & Dehaene, 2004); it is very similar to the instructions of the Perceptual Awareness Scale, only that it includes more levels, which offers

the possibility to distinguish between unimodal and bimodal distributions of ratings (see Sergent & Dehaene, 2004). This measure was general and was further divided in two scales in E2.

In the French version of the instructions, the difference between the two scales was clearer, and the experimenter was present to provide additional information. In the new version of the manuscript we improved the translation to reflect that both scales were distinct except for zero, as was in fact the case (Supplementary note 1).

- In Figure 1, it would be useful to add that the semantic representation is not erased.

This has been added.

- The auditory cue lasted less than 800ms – how long did it last? Why were there 6 premasks? How long was the recording delay?

The auditory cue varied in duration depending on word length, and was buffered with silence at the end if necessary for the interval between cue onset and the following event to last for 800ms. This has been added in the Methods section, lines 951-953.
We used 6 masks so that all possible target word lengths were presented before presentation of the actual target. This was designed to mask the target word's length appropriately and avoid easily detectible variations in image contrast correlated to target word length. This has been added in the Methods section, lines 940-944.
Following the first response, participants had to press the space bar or wait for the end of the 10 second recording delay. This has been added to the Methods lines 959-960.

Reviewer #3 (Remarks to the Author):

Rimsky-Robert et al. elegantly used a retrospective cueing paradigm to examine the prediction that consciousness can be independent of local signal build-up due to the recurrent loops in the sensory system, which is put forth by the global workspace theory. In five experiments, the authors convincingly showed that a retrospective semantically congruent cue boosted the identification of an earlier word, but not the discrimination of low-level visual characteristics, such as case (experiment 1), location (experiment 2). Experiment 3 further showed no increased confidence associated with congruent cues. Finally, experiments 4 and 5 clearly showed that the dissociation between the semantic and local visual feature rescue occurred only to the retro-cues but not pre-cues, suggesting that different mechanisms are involved. In general, I find the manuscript well written, the analyses well executed, and the results are convincing. Still, I have some questions that I hope the authors can address to further strengthen their work.

Major comments

1. I like how the authors used experiment 3 to address the possibility that the increased visibility and detection can be conflated with confidence. Although the results largely refuted this idea, I have to point out that the metacognitive differences in such an ultra-fast identification task could be subtle and hard to detect. For example, a recent study showed higher confidence in an audio-visual illusion (sound-induced flash illusion) when the two modalities were matched (Mayes et al., 2023). This metacognitive component was not particularly considered in the earlier work and was assessed when a variation of the illusion was introduced (Stiles et al., 2018). However, no confidence difference was found in the illusory and non-illusory conditions in this study, which could possibly be attributed to the limited sample size. That is, most studies are equipped with strong enough statistical power to detect the main phenomenon while the same participant pool may not be sufficient to further detect a more nuanced, metacognitive difference. I would like to see this addressed in the context of the current study.

Maynes, R., Faulkner, R., Callahan, G., Mims, C. E., Ranjan, S., Stalzer, J., & Odegaard, B. (2023).

Metacognitive awareness in the sound-induced flash illusion. Philosophical Transactions of the Royal Society B, 378(1886), 20220347.

Stiles, N. R., Li, M., Levitan, C. A., Kamitani, Y., & Shimojo, S. (2018). What you saw is what you will hear: Two new illusions with audiovisual postdictive effects. PloS one, 13(10), e0204217.

We thank the reviewer for pointing this relevant literature; we have added a relating section to the discussion lines 795 to 803.

2. I find the results consistent with an earlier account: reverse hierarchy proposed by Hochstein & Ahissar (2002). In this account, they proposed that conscious perception begins at the end (top) of the sensory system, which allows quick access to gist-like, abstract, or higher-level forms of visual representation. While the more detailed representations come later after scrutiny. This is consistent with the current findings where conscious identification can be rescued by a semantically congruent retro-cue, even when the lower-level features are erased. I find a more thorough discussion on this separation of low-level and high-level sensory signals and their respective interaction with conscious access will greatly improve the impact of the current article.

Hochstein, S., & Ahissar, M. (2002). View from the top: Hierarchies and reverse hierarchies in the visual system. Neuron, 36(5), 791-804.

We agree with the reviewer that our results are compatible with this classical account of conscious visual perception, and that it is important to highlight this connection. We added a relevant section to the discussion lines 858 to 865.

Minor comment

1. The stars in Figure 5 indicate the congruency effect as illustrated in the legend. However, visually it can be confusing since they do not follow the same duration-specific rules as previous figures. I suggest drawing the stars between the two colored lines with an arrow or pointer.

Representations of statistical significance were modified in the figures for better legibility.

Shao-Min Hung

References

Dehaene, S., Naccache, L., Le Clec'H, G., Koechlin, E., Mueller, M., Dehaene-Lambertz, G., van de Moortele, P.-F., & Le Bihan, D. (1998). Imaging unconscious semantic priming. *Nature*, *395*(6702), 597.

Del Cul, A., Baillet, S., & Dehaene, S. (2007). Brain dynamics underlying the nonlinear threshold for access to consciousness. *PLoS Biol*, *5*(10), e260. https://doi.org/07-PLBI-RA-0389%2520%255Bpii%255D%252010.1371/journal.pbio.0050260

Garnier-Allain, A., Pressnitzer, D., & Sergent, C. (2023). Retrospective cueing mediates flexible conscious access to past spoken words. *Journal of Experimental Psychology. Human Perception and Performance*, *49*(7), 949-967. https://doi.org/10.1037/xhp0001132

Kouider, S., & Dehaene, S. (2007). Levels of processing during non-conscious perception : A critical review of visual masking. *Philos Trans R Soc Lond B Biol Sci*, *362*, 857-875.

Kouider, S., & Dupoux, E. (2004). Partial awareness creates the "illusion" of subliminal semantic priming. *Psychological science*, *15*(2), 75-81.

Sandberg, K., Timmermans, B., Overgaard, M., & Cleeremans, A. (2010). Measuring consciousness : Is one measure better than the other? *Consciousness and Cognition*, *19*(4), 1069-1078. https://doi.org/10.1016/j.concog.2009.12.013

Sergent, C., & Dehaene, S. (2004). Is consciousness a gradual phenomenon ? Evidence for an all-or-none bifurcation during the Attentional Blink. *Psychol Sci*, *15*(11), 720-728.

Sergent, C., Wyart, V., Babo-Rebelo, M., Cohen, L., Naccache, L., & Tallon-Baudry, C. (2013). Cueing attention after the stimulus is gone can retrospectively trigger conscious perception. *Current Biology*, *23*(2), 150-155.

Dear Editors at Communications Psychology,

We would like to thank the reviewers again for their attentive proofing which has greatly helped us improve our article. Our responses are written in blue, and references to the changes we applied in the new version of the manuscript are highlighted in yellow.

Reviewer #1 (Remarks to the Author):

This revised manuscript addresses all of my comments. It presents a nice series of experiments with interesting and very results that replicate well across experiments. Although the theoretical scope of the findings and some of assumptions in the analysis of the data might still be up for discussion, the manuscript presents them in a convincing way. I think many readers will find this an fascinating study and I therefore fully endorse its publication.

We would like to thank again the reviewer for their help in this process and their appreciative comments.

Reviewer #2 (Remarks to the Author):

I was a reviewer on the previous version of this paper. I appreciate that the authors clarified many of the imprecisions that made understanding the previous version somewhat challenging. However, Iit seems to me that these clarifications raised more concerns than they resolved.

We thank Dr Lamy for a very thorough reviewing of our article; it has already helped us improve it substantially. We also thank her for signing the review, we are very honored to receive advise from one of the best experts in the field. We are sorry that Dr Lamy is under the impression that this revised version raised more concerns than it resolved; we think we can satisfactorily address all of them and we have used them to further consolidate our argumentation and nuance our discussion.

1. I will start with the control experiments. Although the authors explained their motivation for using repetition cues instead of associative cues, it seems to me that this change created a problem: specifically, participants face a very different situation in the pre-cue and retro-cue conditions. In the former, when the pre-cue is a word, participants can focus exclusively on the case of the target because they already know the identity of the word, and all they will have to do is repeat it - whereas when the pre-cue is neutral, they have to divide their attention between the word meaning and its case, hence the observed “congruence” effect. By contrast, with the retro-cue, since participants do not know in advance whether or not the

cue will be a word, they have to divide their attention in both conditions, hence the absence of an effect.

Dr Lamy raises an alternative interpretation to the results of our control experiment. Individuals might have to split their attention between the word's case and the word's identity: when the cue is congruent and appears before the target, it provides participants with the correct word identity and allows them to allocate all their attentional resources to the word's case. This could explain the effect of congruent pre-cues on case discrimination performance: because individuals do not need to split their attention, they display significantly better performance at the case discrimination task. For retro-cues, their attention would have to remain split until the cue arrives, after the stimulus, therefore dampening the effect on case discrimination.

We recognize that this could have been a possible interpretation had participants been instructed to perform both case discrimination and word identification. But importantly, in these control experiments individuals did not have to report the word's identity: they only had to report the word's case and its visibility (cf lines 653-655 of previous version: "participants only had to report letter-casing and visibility of the whole stimulus. This maximized our chance to observe cueing effects at the level of visual representations, since participants were focused on the visual attributes of the target."). It is particularly telling that, despite focusing exclusively on case, participants are still not better at case discrimination when a retro-cue provides them the word's identity, although their detection sensitivity is improved. Again, this renders the partial awareness hypothesis particularly unlikely, which is the purpose of this control: if retro-cueing effects were due to combining a glimpse of a few letters with a cue on the word's identity, improved case discrimination should at least be observed in these conditions where the only focus is on case and the identity of the word is directly provided.

In conclusion, Dr Lamy's concern seems to arise from the notion that individuals reported the word's identity at the end of each trial in the controls, but this was not the case. Thanks to this comment we realize that this significant change in protocol should be highlighted even further. To this effect, protocol panels were updated in each figure, and a particular emphasis has been put on this point in the description and conclusion of Experiments 4-7 (lines 893-895 and lines 938-941).

We would also like to emphasize that these control experiments are far from being the only control we have: they complement several control analyses performed on each of the main experiments which all converge onto suggesting that partial awareness cannot entirely account for the effects we observe, and that our results instead reflect a genuine retrospective effect on conscious perception itself.

2. Relatedly, it is not clear in the first place why one would expect a semantic cue to retrieve / enhance the case of the target. The authors did not flesh out the argument. The results of the control experiments conveyed the impression that, somehow, the cue did increase case-discrimination performance when it was a pre-cue, and I confess that the demonstration initially convinced me - but as I explained above, it seems to me that the finding arose from a confound. I apologize for not spotting this problem in the earlier version.

First, as we explain in response to point 1, there is no confound in terms of split attention in our findings that pre-cues improve both detection and case discrimination. But it is true that we did not explain in detail why we should expect semantic cues to improve case discrimination or other visual features, at least with pre-cues.

In all our experiments, the semantic cues are known to be potentially informative about what word is likely to be presented on screen. When hearing the cue, participants could form not only semantic but also visual expectations, since they have a better template of what the word could look like. For example, if the visual word was “bee”, the auditory cue “hive” should reactivate the traces that the visual word has left at the lexical level, but this should also allow to form better expectations about the visual form of the word: a short word with a “b” at the beginning and a double “e”, enhancing whatever traces are left in the visual word form area, or lower, matching this orthographic template. If such visual traces exist, then their signal to noise ratio should increase with this cueing, improving the retrieval of these traces. Such cueing by visual expectations is particularly encouraged in the present studies since participants are always explicitly asked to report a visual feature of the word, either case or position on screen, and even more so in the control experiment where they know the word identity and only have to focus on letter-casing. And indeed, in pre-cueing we do observe cueing on case discrimination: congruent pre-cues presumably boost the signal to noise ratio of the visual form of the word as early as the feed-forward sweep, hence preventing the backward masks from disrupting its processing, resulting in improved case discrimination. The absence of such effect for retro-cueing, despite improved detection, suggests that masking prevents the visual form of the word to be retrieved retrospectively.

More generally, several other studies have shown strong effects of semantic cueing on visual expectations. For example Lupyan and Ward have investigated the effect of semantic cueing by auditory words on subsequent recognition of visual objects, and found that the detection of masked images was improved when the object referred to by the cue word was similar in shape to the image (Lupyan & Ward, 2013).

Thanks to this remark from the reviewer, we realized that these points had to be made explicit in the text. We have updated the manuscript accordingly lines 133-139, and then again in the discussion lines 968-969.

3. The authors refer to a dissociation between word detection/identification and case discrimination, but I still cannot figure out what the dissociation is. If I understood correctly, the authors rely on two findings: (a) the congruence effects occur for meaning and not for case – but see my above question about why they should and the alternative account of the control experiments' results; (b) On the fraction of trials where targets were retro-perceived, case discrimination was not at ceiling – but performance was rather high (around 70%) and when it was at chance, the CI was huge.

The point raised by (a) was discussed in the previous paragraphs. The dissociation between word identification and case discrimination we refer to corresponds to the fact that when individuals detect and identify the word, they appear less likely to also know accurate information about its case in the congruent condition than in the incongruent condition. Indeed, when we investigated case-discrimination performance for trials where the word was correctly identified (panel (e) in Exp 1, (f) in Exps 2 & 3) we found a very clear and replicable ***decrease*** in case discrimination performance for correctly identified words in the congruent condition compared to the incongruent one; this observation alone suggests the aforementioned dissociation (red vs. deep blue bars).

We initially went a step further and estimated performance at the case/position discrimination tasks specifically for retro-perceived trials by relying on conditional probability analyses (light blue bars in panels e/f). Dr Lamy is correct in pointing out that these estimates were noisy and difficult to interpret; it was already acknowledged in the previous version of the manuscript. Since it brought little more to the interpretation of our results than already did the comparison between congruent and incongruent conditions, we have removed this analysis from the new version of the manuscript.

4. Figure 1 suggests that sensory features are erased whereas semantic ones are preserved but there is no evidence for that: it is entirely possible that the sensory feature traces exist and remain unconscious because the cue is not useful for retrieving / enhancing them and bring them to consciousness. Alternatively, they could survive the mask but decay faster (a bit in the same vein as in Posner's classical 1969 paper on the time course of AA vs. Aa representations).

As we argue in response to point 2, the cue *should* be useful for retrieving visual features, since it sharpens visual expectations; and indeed, we demonstrate that it is effective when presented before the stimulus. However, retro-cues, despite being potentially useful, are ineffective on low-level features.

We agree that the depiction of visual masking in the previous version of our Figure 1 was too schematic. Indeed, it suggested that sensory features were completely erased by the mask, which might not be accurate. The classical view on visual masking rather suggests that while the feedforward sweep occurs early and automatically enough to be undisturbed by masking, re-entrant processes are disrupted by a conflict between what is encoded in higher-level areas (target-related information) and lower-level areas (mask only or mask and target-related information)(Enns & Di Lollo, 2000; Fahrenfort et al., 2007; Super et al., 2001). We have therefore updated Figure 1 to reflect more precisely and accurately these mechanisms. We have also improved our description of these mechanisms in the text, lines 107-109. Furthermore, we now acknowledge in the discussion the limitations of the masking paradigm, where it has been shown that unconscious activations following masking are fragile and decay rapidly, even at the semantic level (lines 1042-1045):

> *"[…] a balance must be struck between the intensity of visual masking and the unmasking potential of the cue. In particular, semantic processing of masked words is highly sensitive to masking strength (Kouider & Dehaene, 2007) and decays rapidly over time (Avneon & Lamy, 2018; Micher & Lamy, 2023)."*

The possibility that the visual features decay faster than semantic ones cannot be tested from our dataset since we did not manipulate the delay between target and response screen. However, this cannot account for the difference between congruent and incongruent conditions, since the delay was the same.

5. I found the description of the recurrent processing theory as a "local loops" framework misleading: in Lamme's theory, local loops support phenomenal awareness whereas longer-range loops support report - which is what the authors measured here. I still think that the findings cannot disentangle the two models.

We agree that the local recurrency hypothesis proposes that we can become phenomenally conscious when local recurrency happens, and that reportability is optional, comes on top of this phenomenal awareness, and is supported by longer-range loops. According to this model, there can be phenomenal consciousness in the absence of access if local recurrency occurs in the absence of long-range connectivity (a proposition that is disputed, see Dennett and Cohen(Cohen & Dennett, 2011)). But conversely, no conscious perception, neither phenomenal nor access, should be possible in the absence of local recurrency. Since masking

disrupts local recurrency and we still observe improved conscious access (improved detection sensitivity) with our retrospective cue, it suggests that awareness of the presence of the word can occur even when local recurrencies at the visual level are disrupted. This observation constitutes a challenge to the local recurrency proposition, although it is not a definitive proof.

This more precise argumentation has been added in the discussion lines 1053 to 1064

6. In short, I think that what the study shows is that different aspects of a stimulus can be selectively brought to conscious perception by retro-cues: here, the target's meaning by a cue that is useful to rescue it from unconsciousness (a finding that is in line with the framework suggested by Kouider et al., 2010). In passing, I think that this paper is relevant here not only because of the partial awareness argument but also for its theoretical claims (namely, their hypothesis that "representations at each level can be accessed independently from each other" (p. 304).

We fully agree with this statement and we added this remark to the discussion (lines 1106-1112)

We would like to highlight that, although this notion of independent access to different levels of representations has already been explored, the present studies bring additional evidence showing that such selective access can occur retrospectively, after the stimulus has disappeared, and that even the capacity to detect the presence of a visual word can be dissociated from the ability to report its visual features.

References

Kouider, S., De Gardelle, V., Sackur, J., & Dupoux, E. (2010). How rich is consciousness? The partial awareness hypothesis. Trends in cognitive sciences, 14(7), 301-307.

Posner, M. I., Boies, S. J., Eichelman, W. H., & Taylor, R. L. (1969). Retention of visual and name codes of single letters. Journal of experimental psychology, 79(1p2), 1.

Dominique Lamy

Reviewer #3 (Remarks to the Author):

The authors have addressed my previous comments adequately. I am happy to see this study added to the literature.

We would like to thank the reviewer for their help in improving our manuscript and their appreciative comments.

References

Avneon, M., & Lamy, D. (2018). Reexamining unconscious response priming: A liminal-prime paradigm. *Consciousness and Cognition*, *59*, 87–103. https://doi.org/10.1016/j.concog.2017.12.006

Cohen, M. A., & Dennett, D. C. (2011). Consciousness cannot be separated from function. *TRENDS in Cognitive Sciences*, *15*(8), 7.

Enns, J. T., & Di Lollo, V. (2000). What's new in visual masking? *Trends in Cognitive Sciences*, *4*(9), 345–352.

Fahrenfort, J. J., Scholte, H. S., & Lamme, V. A. (2007). Masking disrupts reentrant processing in human visual cortex. *Journal of Cognitive Neuroscience*, *19*(9), 1488–1497.

Kouider, S., & Dehaene, S. (2007). Levels of processing during non-conscious perception: A critical review of visual masking. *Philos Trans R Soc Lond B Biol Sci*, (362), 857–875.

Lupyan, G., & Ward, E. J. (2013). Language can boost otherwise unseen objects into visual awareness. *Proceedings of the National Academy of Sciences*, *110*(35), 14196–14201. https://doi.org/10.1073/pnas.1303312110

Micher, N., & Lamy, D. (2023). The role of conscious perception in semantic processing: Testing the action trigger hypothesis. *Consciousness and Cognition*, *107*, 103438. https://doi.org/10.1016/j.concog.2022.103438

Super, H., Spekreijse, H., & Lamme, V. A. (2001). Two distinct modes of sensory processing observed in monkey primary visual cortex (V1). *Nat Neurosci*, *4*(3), 304–310.
