# Supplementary material for: Consciously detecting and recognizing a past visual word after its sensory trace is gone
Source: Commun Psychol. 2026 Jun 9;4:137. doi: 10.1038/s44271-026-00478-9 (PMC13658779; doi:10.1038/s44271-026-00478-9)
Supplement: Supplementary file 2 [file 44271_2026_478_MOESM2_ESM.pdf]

Supplementary Information

Consciously detecting and recognizing a past visual word after its sensory trace is gone

Daphné Rimsky-Robert[1], Matteo Lisi[2], Kevin Nguy[1], Roxane Jannin[1], Thomas Hardy[1], Nathan Beraud[1], Claire Sergent[1]

[1] Integrative Neuroscience and Cognition Center - UMR 8002 CNRS/Université Paris-Cité

2 Department of Psychology, Royal Holloway, University of London, UK

Corresponding author:
Daphné Rimsky Robert
Email: drr@tuta.io, daphne.rimsky-robert@aphp.fr

Supplementary Notes

Supplementary Note 1: Instructions.

Here are the general instructions provided to the participants in the various experiments (instructions relative to Experiment 2 are in between parentheses):

On each experimental trial, you will see a circle and a dot in its center. You will have to fixate this circle. The eyetracker follows your gaze, and waits until you are fixating to start. When the trial starts, you will see character strings appear in the center of the screen (expt2: above and below the center). They will make no sense at all. Among them, most of the time, there will be a meaningful word written in uppercase or lowercase (expt2: in uppercase), but in about 10% of trials, there will be no word at all. (Please make sure to fixate the central dot, otherwise you will not be able to read properly.) After this visual sequence, you will hear another, different word in the headset you are wearing. This word will be related to the written word half the time e.g. witch, broom. This word can help you, or have no effect at all, try and pay attention to it.

The task instructions for Experiment 1 were:

You will answer 3 questions:

1) *What was the written word among the nonsensical strings? If you do not know, say whatever comes to your mind. You should always provide an answer, and try and say just one word. If you're having difficulties coming up with different words every time, you can say the same one when you really don't know, but we encourage you to be spontaneous, it's more fun. "I don't know" is not a valid answer.*
2) *Was this word in uppercase or lowercase?*
3) *How well did you see this word? You have a scale ranging from 0 to 8, choose the appropriate number. 0 is special: it means you have not seen the word at all. 1 corresponds to "maybe a glimpse of something", and 8 means you have perfectly seen the word.*

The task instructions for Experiment 2 were:

You will answer 4 questions:

1) *Was the word presented above or below the fixation point?*
2) *How well did you see the word* ***and its position****? You have a colored scale ranging from red to green, with 9 points.*

   0: I have not seen the stimulus, not even a glimpse of a letter.

   1-4: I have seen something, I may have seen a few letters, but I'm not really sure ***where*** *(focus on position) this was.*

   5-8: I have seen the word ***and its position****. Increase with stronger sensation of having seen the word and its position.*

3) *What was the word written among the nonsensical strings? If you don't know, say whatever comes to mind. Always provide an answer, try and say just one word. If you're having difficulty coming up with different words every time, you can say the same one when you really don't know, but we encourage you to be spontaneous, it's more fun. "I don't know" is not a valid answer.*
4) *How well did you see the word* ***and its identity****? You have a colored scale ranging from red to green, with 9 points.*

   0: I have not seen the stimulus, not even a glimpse of a letter.

   1-4: I have seen something, I may have seen a few letters, but I'm not really sure ***what*** *(focus on identity/reading) this was.*

   5-8: I have seen the word ***and its identity****. Increase with stronger sensation of having seen the word and its identity*

The task instructions for Experiment 3 were:

You will answer 4 questions:

1) *Was this word in uppercase or lowercase?*

2) *How confident are you in this uppercase/lowercase response? You have a colored scale ranging from red to green, with 9 points. The lowest rating (completely red) corresponds to no confidence at all, and the highest means you are fully confident.*

   0: not confident at all (reported at chance).

   1-4: I am not very confident that I correctly reported the word's case.

   5-8: I am more and more confident that I correctly reported the word's case.

3) *What was the word written among the nonsensical strings? If you don't know, say whatever comes to mind. Always provide an answer, try and say just one word. If you're having difficulty coming up with different words every time, you can say the same one when you really don't know, but we encourage you to be spontaneous, it's more fun. "I don't know" is not a valid answer.*

4) *How confident are you with your word identity response? You have a colored scale ranging from red to green, with 9 points. The lowest rating (completely red) corresponds to no confidence at all, and the highest means you are fully confident.*

   0: not confident at all (reported at chance).

   1-4: I am not very confident that I correctly reported the word's identity.

   5-8: I am more and more confident that I correctly reported the word's identity.

   The two confidence measures are distinct: you may be confident about the word's case because you caught a single letter, but not know what the word was. The reverse is also possible. Freely reply to each rating separately.

In Experiment 4 & 5, participants received near-identical instructions, however they were not warned that the neutral cues were reversed audio files for the congruent cues. Instructions for use of the visibility scale were as in Experiment 1.

Supplementary Note 2: Results of post-hoc comparisons in all Experiments

Experiment 1, word identification (2b): 12ms, 95%BCI-diff [-0.03 0.09]; 24ms, 95%BCI-diff [0.002, 0.16]; 36ms, 95%BCI-diff [0.02, 0.21]; 48ms, 95%BCI-diff [0.02, 0.25].

Experiment 1, detection sensitivity (2d): 12ms (95%CI = [-0.03, 0.04] d = 0.08, 95%CI = [-0.49 0.58]), 24ms (95%CI = [-0.009, 0.08] d =0.51, 95%CI = [-0.02 0.99]), 36ms (95%CI = [0.03, 0.08] d = 1.36, 95%CI = [0.97 2.38]), 48ms (95%CI = [0.01, 0.05] d =0.84, 95%CI = [0.49 1.49]).

Experiment 1, visibility ratings – overall (Supplementary 2): 12ms (95%CI = [-0.19 0.17] d = -0.04, 95%CI = [-0.57 0.54]), 24ms (95%CI = [0.03 0.56] d = 0.72, 95%CI = [0.22 1.71]), 36ms (95%CI = [0.77 1.55] d = 1.74, 95%CI = [1.30 2.71]), 48ms (95%CI = [0.54 1.68] d = 1.17, 95%CI = [0.81 1.99]).

Experiment 2, word identification (3b): 12ms (95%BCI-diff [-0.05 0.04]), 24ms (95%BCI-diff [-0.02 0.06]), 36ms (95%BCI-diff [-0.01 0.12]), 48ms (95%BCI-diff [-0.005 0.18]).

Experiment 2, detection sensitivity word (3d): 12ms (95%CI [-0.07 0.01], d = 0.40, 95%CI = [-0.98 0.1]), 24ms (95%CI [-0.02 0.04], d = 0.15, 95%CI = [-0.35 0.58]), 36ms (95%CI [0.02 0.08], d = 0.90, 95%CI = [0.45 1.76]), 48ms (95%CI [0.03 0.09], d = 1.03, 95%CI = [0.68 1.71]).

Experiment 2, detection sensitivity position (3e): 12ms (95%CI [-0.03 0.03], d = 0.09, 95%CI = [-0.71 0.3]), 24ms (95%CI [-0.03 0.04], d = 0.01, 95%CI = [-0.52 0.56]), 36ms (95%CI [0.01 0.07], d = 0.65, 95%CI = [0.21 1.24]), 48ms (95%CI [0.01 0.05], d = 0.79, 95%CI = [0.39 1.41]).

Experiment 2, visibility ratings – word (Supplementary 3): 12ms (95%CI = [-0.36 0.07] d = 0.35, 95%CI = [-0.83 0.11]), 24ms (95%CI = [-0.02 0.46] d = 0.46, 95%CI = [0.05 2.45]), 36ms (95%CI = [0.49 1.08] d = 1.51, 95%CI = [1.01 2.45]), 48ms (95%CI = [0.82 1.62] d = 1.65, 95%CI = [1.29 2.82]).

Experiment 2, visibility ratings – position (Supplementary 3): 12ms (95%CI = [-0.22 0.19] *d* = 0.07, 95%CI = [-0.70 0.38]), 24ms (95%CI = [-0.22 0.35] *d* = 0.12, 95%CI = [-0.36 0.59]), 36ms (95%CI = [0.21 0.69] *d* = 0.69, 95%CI = [0.59 2.12]), 48ms (95%CI = [0.36 0.74] *d* = 0.74, 95%CI = [0.83 2.11]).

Experiment 3, word identification (4b): 12ms (95%BCI-diff [-0.05 0.03]), 24ms (95%BCI-diff [-0.003 0.09]), 36ms (95%BCI-diff [0.06 0.18]), 48ms (95%BCI-diff [0.06 0.25]).

Experiment 3, confidence sensitivity word (4d): 12ms (95%CI [-0.03 0.01], *d* = 0.39, 95%CI = [-0.97 0.05]), 24ms (95%CI [-0.003 0.6], *d* = 0.53, 95%CI = [0.14 1.06]), 36ms (95%CI [0.01 0.1], *d* = 0.69, 95%CI = [0.27 1.35]), 48ms (95%CI [0.03 0.11], *d* = 0.87, 95%CI = [0.63 1.26]).

Experiment 3, confidence sensitivity case (4e): 12ms (95%CI [-0.01 0.03], *d* = 0.13, 95%CI = [-0.42 0.52]), 24ms (95%CI [-0.04 0.02], *d* = 0.26, 95%CI = [-0.85 0.26]), 36ms (95%CI [-0.03 0.05], *d* = 0.16, 95%CI = [-0.27 0.92]), 48ms (95%CI [0.02 0.08], *d* = 1.01, 95%CI = [0.62 1.69]).

Experiment 3, confidence ratings – word (Supplementary 4): 12ms (95%CI = [-0.17 0.06] *d* = 0.30, 95%CI = [-0.87 0.14]), 24ms (95%CI = [0.21 0.774] *d* = 0.74, 95%CI = [0.48 1.74]), 36ms (95%CI = [0.85 1.96] *d* = 1.38, 95%CI = [0.91 2.28]), 48ms (95%CI = [1.31 2.44] *d* = 1.80, 95%CI = [1.39 2.76]).

Experiment 3, confidence ratings – case (Supplementary 4): 12ms (95%CI = [-0.12 0.23] *d* = 0.12, 95%CI = [-0.46 0.57]), 24ms (95%CI = [-0.33 0.18] *d* = 0.14, 95%CI = [-0.64 0.34]), 36ms (95%CI = [-0.09 0.60] *d* = 0.48, 95%CI = [-0.002 1.28]), 48ms (95%CI = [0.29 0.76] *d* = 1.23, 95%CI = [0.66 2.23]).

Experiments 4-7, detection sensitivity, pre-cued trials (5a): 12ms (95%CI = 12ms [-0.06 0.01], *d* = 0.31, 95%CI = [-0.02 0.69]), 24ms (95%CI = [-0.0003 0.08] , *d* = 0.38, 95%CI = [-0.04 0.79]), 36ms (95%CI = [0.001 0.07], *d* = 0.39, 95%CI = [0.1 0.73]), 48ms (95%CI = [0.01 0.07], *d* = 0.51, 95%CI = [0.25 0.82])

Experiments 4-7, detection sensitivity, retro-cued trials (5a): 12ms (95%CI = 12ms [-0.03 0.04], *d* = 0.11, 95%CI = [-0.22 0.48]), 24ms (95%CI = [-0.01 0.07] , *d* = 0.27, 95%CI = [-0.05 0.58]), 36ms (95%CI = [0.01 0.06], *d* = 0.64, 95%CI = [0.36 0.94]), 48ms (95%CI = [0.002 0.03], *d* = 0.33, 95%CI = [0.01 0.65])

Experiments 4-7, case performance, pre-cued trials (5b): 12ms (95%BCI-diff [-0.02 0.06]), 24ms (95%BCI-diff [0.01 0.06]), 36ms (95%BCI-diff [0.01 0.06]), and 48ms (95%BCI-diff [0.004 0.04])

Experiments 4-7, case performance, retro-cued trials (5b): 12ms (95%BCI-diff [-0.02 0.05]), 24ms (95%BCI-diff [-0.02 0.03]), 36ms (95%BCI-diff [-0.02 0.03]), and 48ms (95%BCI-diff [-0.03 0.02])

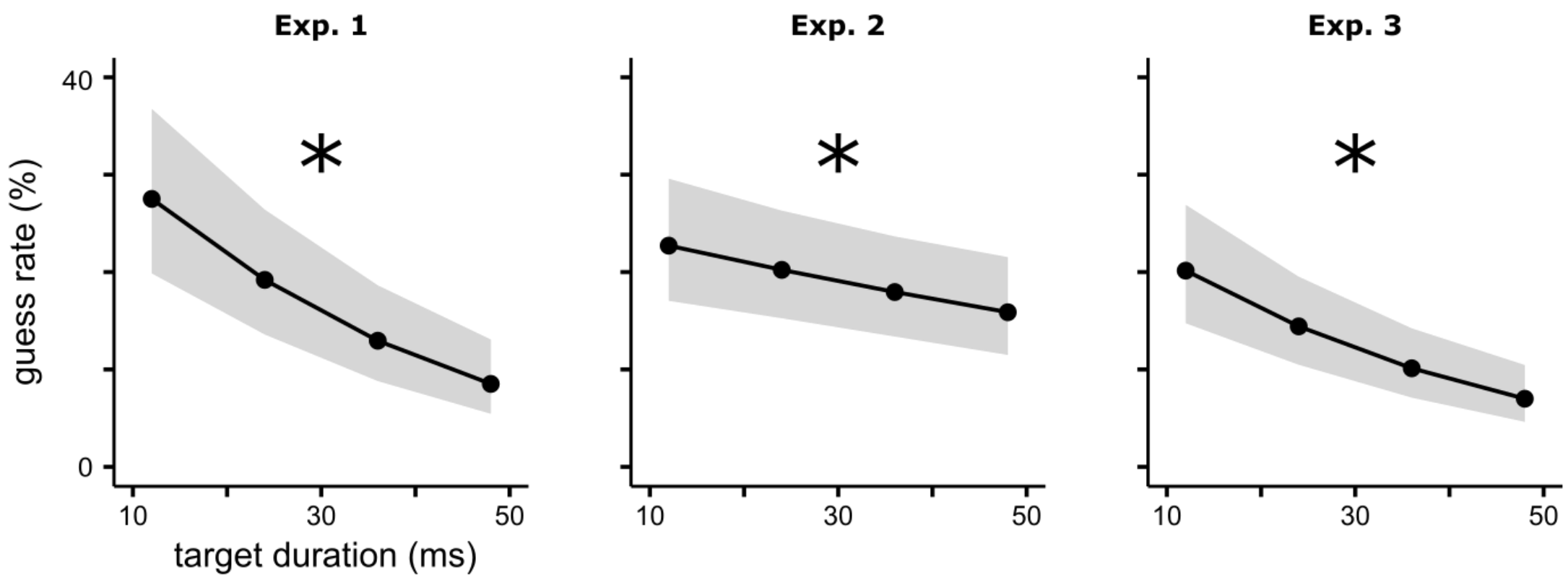

Supplementary Figure 1: guess rate as a function of target duration. Guessed trials were defined as trials in the incongruent condition in which participants reported the word associated to the cue (which is as a wrong answer in incongruent trials). A generalized linear mixed-effect model was used to model the guess rate using a logistic regression testing for the fixed-effect of target duration vs. an intercept-only model. The black line represents the posterior estimate of the guess rate, the shaded area is the 95%BCI of the estimated values. Participant number was used as a random-effect on the intercept. Stars denote a significant main effect of target duration.

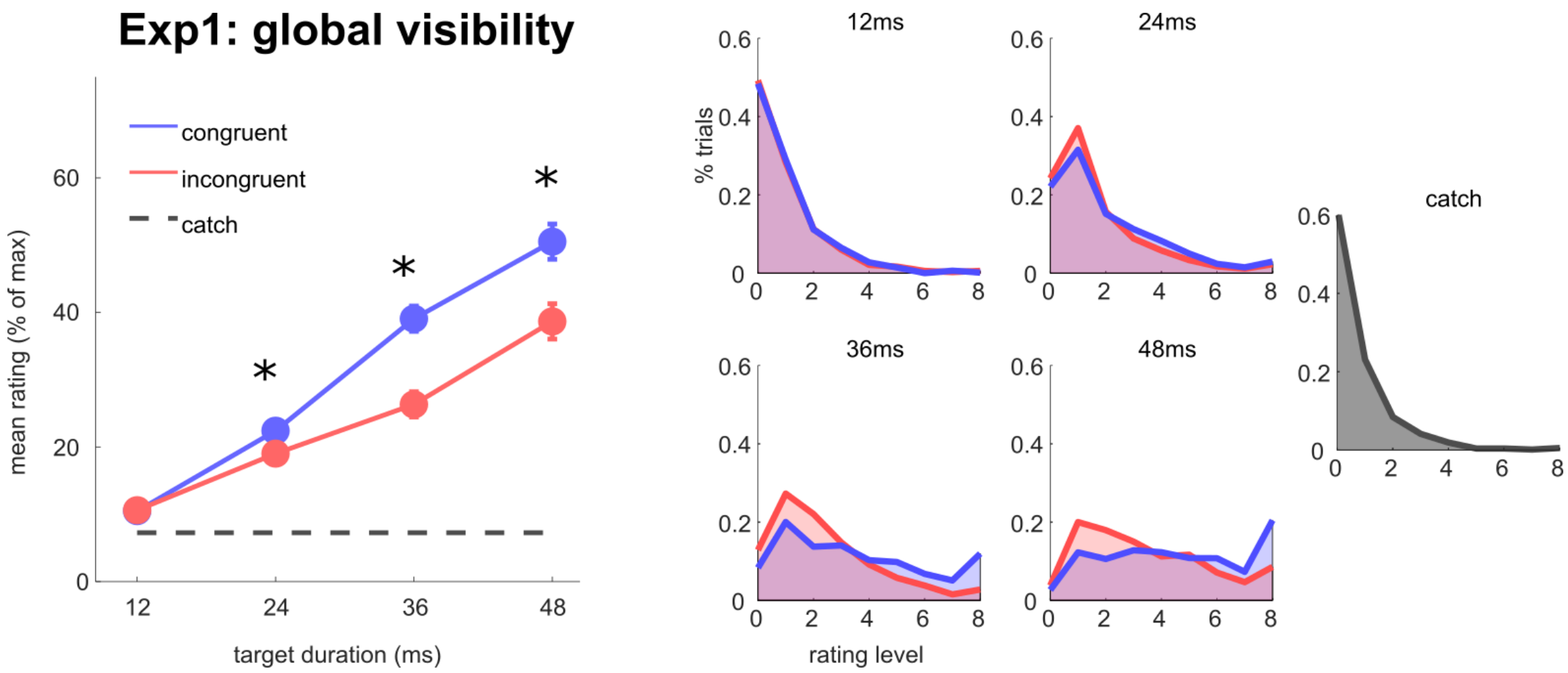

Supplementary Figure 2: Mean visibility ratings and distributions (Experiment 1). Left: each dot represents the average across participants of the mean visibility rating obtained on the scale for catch (grey), congruent trials (blue) or incongruent trials (red) as a function of target duration, as well as mean visibility obtained for catch trials (dashed grey). The error bars represent the standard error of the mean difference between congruent and incongruent trials. A repeated measures two-way ANOVA on mean subjective visibility ratings revealed a significant increase in visibility with target duration ($F(1.23,17.29)=81.34$, $p<0.001$, $\eta^2G = 0.55$, 95%CI for η^2G = [0.30 0.66]) and congruence ($F(1.00,14.00)=38.43$, $p<0.001$, $\eta^2G = 0.08$, 95%CI for η^2G = [0.00 0.33]), and a significant interaction between these two factors ($F(3.00,42.00)=15.812$, $p<0.001$, $\eta^2G = 0.05$, 95%CI for η^2G =[0.00 0.17]). Distributions of visibility ratings are shown on the right for each target duration and for catch trials. Notice that even for catch trials, participants sometimes used visibilities higher than 0 to express that they might have felt a glimpse of the stimulus. Detection sensitivity is obtained by comparing each of the distributions to the distribution obtained on catch trials (see Methods). Stars represent significant post-hoc comparisons at each target duration (see **Supplementary Note 2**).

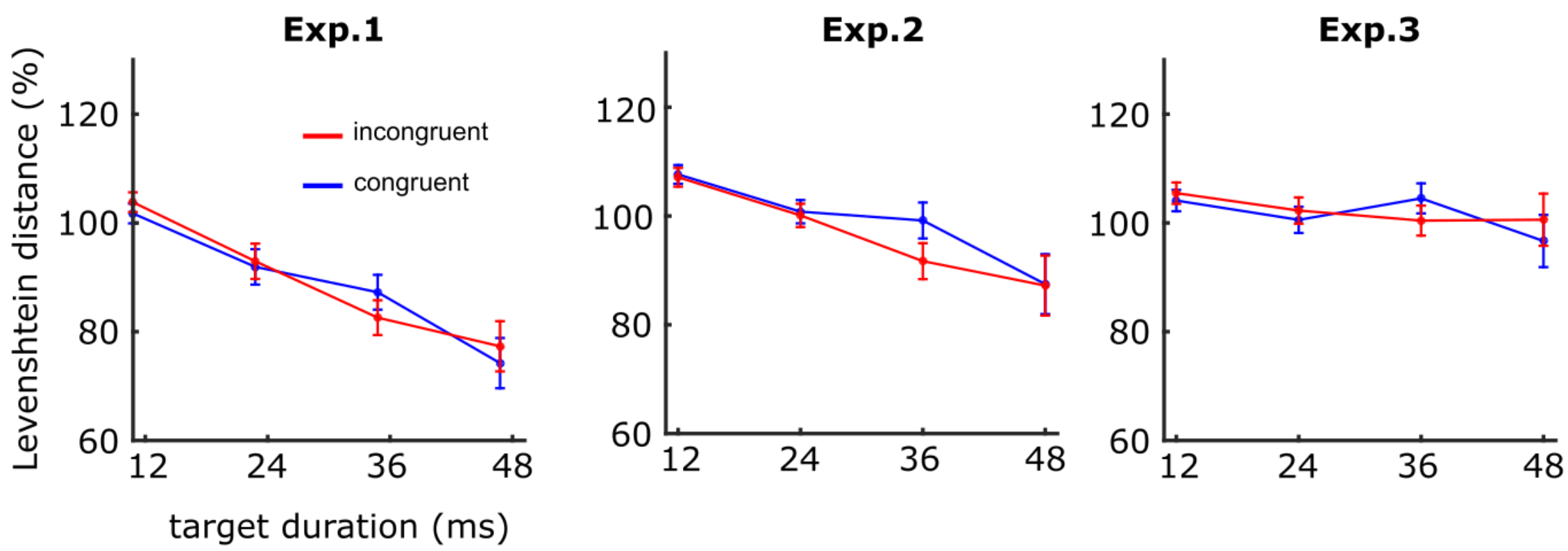

Supplementary Figure 3: orthographic distance (Levenshtein distance) between erroneous identification responses and the actual target word. The Levenshtein distance between target and response in word-incorrect trials is shown as a function of target duration and cue congruency. In Experiment 1, the repeated measures ANOVA (duration x congruency) showed an effect of duration ($F(3,42)=40.16$, $p< 0.001$, $\eta^2G = 0.42$, 95%CI for η^2G = [0.15 0.56]) but no effect of congruency ($F(1,14)=0.05$, $p=0.82$, $\eta^2G = 0.0003$, value too low for estimating CI) nor an interaction ($F(3,42)=1.055$, $p=0.38$, $\eta^2G = 0.02$, 95%CI for η^2G = [0.00 0.07]). In Experiment 2, the repeated measures ANOVA (duration x congruency) showed an effect of duration ($F(3,51)=3.58$, $p=0.02$, $\eta^2G = 0.05$, 95%CI for η^2G = [0.00 0.15]) but not effect of congruency ($F(1.00,17.00)=0.209$, $p=0.65$, $\eta^2G = 0.001$, 95%CI for η^2G = [0.00 0.04]) nor an interaction ($F(3,51)=1.15$, $p=0.34$, $\eta^2G = 0.02$, 95%CI for η^2G = [0.00 0.09]). In Experiment 3 the repeated measures ANOVA (duration x congruency) showed an effect of duration ($F(2.17,36.93)=22.790$, $p<0.001$, $\eta^2G = 0.26$, 95%CI for η^2G = [0.05 0.40]) but not effect of congruency ($F(1.00,17.00)=1.891$, $p=0.19$, $\eta^2G = 0.008$, 95%CI for η^2G = [0.00 0.09]) nor an interaction ($F(1.68,28.57)=0.960$, $p=0.39$, $\eta^2G = 0.01$, 95%CI for η^2G = [0.00 0.07]). Note that for some estimates, the number of trials per participant was quite low, especially for longer target durations as only incorrect trials for word identification are used. However, the average number of trials in the 48ms condition (out of a total of 96 trials) was still reasonable in all three experiments: Experiment 1 [43.5], Experiment 2 [63.2], Experiment 3 [42.1]). Error bars represent the standard error of the mean.

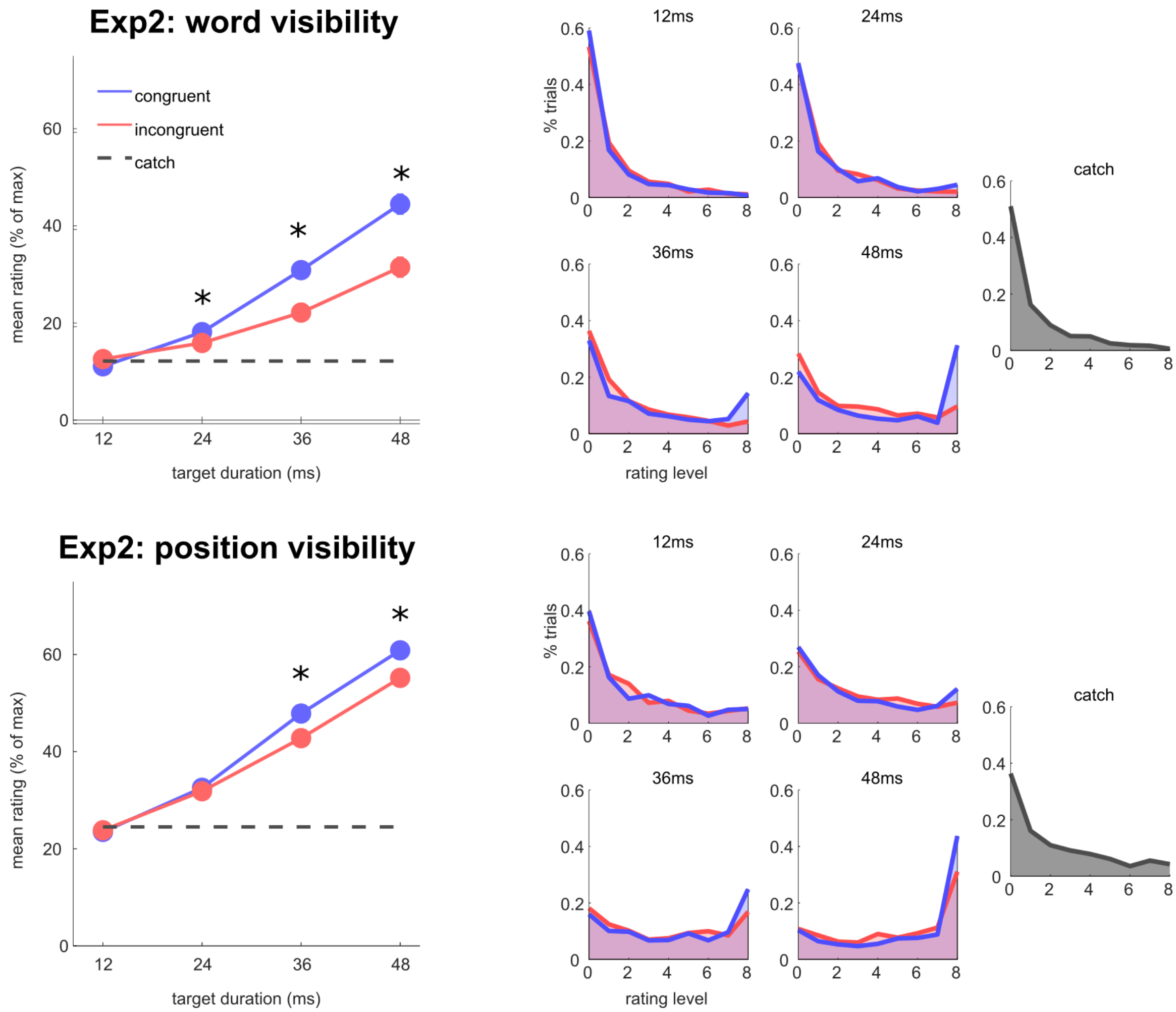

Supplementary Figure 4: Mean visibility ratings and distributions in Experiment 2, separately for word and position. In experiment 2, participants rated separately the visibility of the word's identity (upper panel) and position (lower panel). Each dot represents the average across participants of the mean visibility rating obtained for catch (grey), congruent trials (blue) or incongruent trials (red) as a function of target duration. The error bars represent the standard error of the mean difference between congruent and incongruent trials. A repeated measures two-way ANOVA on mean visibility ratings for word-meaning revealed a significant increase with target duration ($F(1.19,20.28)=41.70$, $p<0.001$, $\eta^2G = 0.26$, 95%CI for η^2G = [0.05 0.41]) and congruence ($F(1.00,17.00)=55.43$, $p<0.001$, $\eta^2G = 0.03$, 95%CI for η^2G = [0.00 0.17]), with an interaction between these two factors ($F(3.00,51.00)=24.05$, $p<0.001$, $\eta^2G = 0.03$, 95%CI for η^2G = [0.00 0.10]). A repeated measures two-way ANOVA on mean visibility ratings for position revealed a similar pattern: a significant increase with target duration ($F(1.35,22.94)=64.103$, $p<0.001$, $\eta^2G = 0.33$, 95%CI for η^2G = [0.10 0.47]) and congruence ($F(1.00,17.00)=30.305$, $p<0.001$, $\eta^2G = 0.006$, 95%CI for η^2G = [0.00 0.08]), with a significant interaction between these two factors ($F(2.46,41.82)=6.721$, $p=0.002$, $\eta^2G = 0.005$, 95%CI for η^2G = [0.00 0.01]).

Distributions of visibility ratings are shown on the right. Stars represent significant post-hoc comparisons at each target duration (see **Supplementary Note 2**).

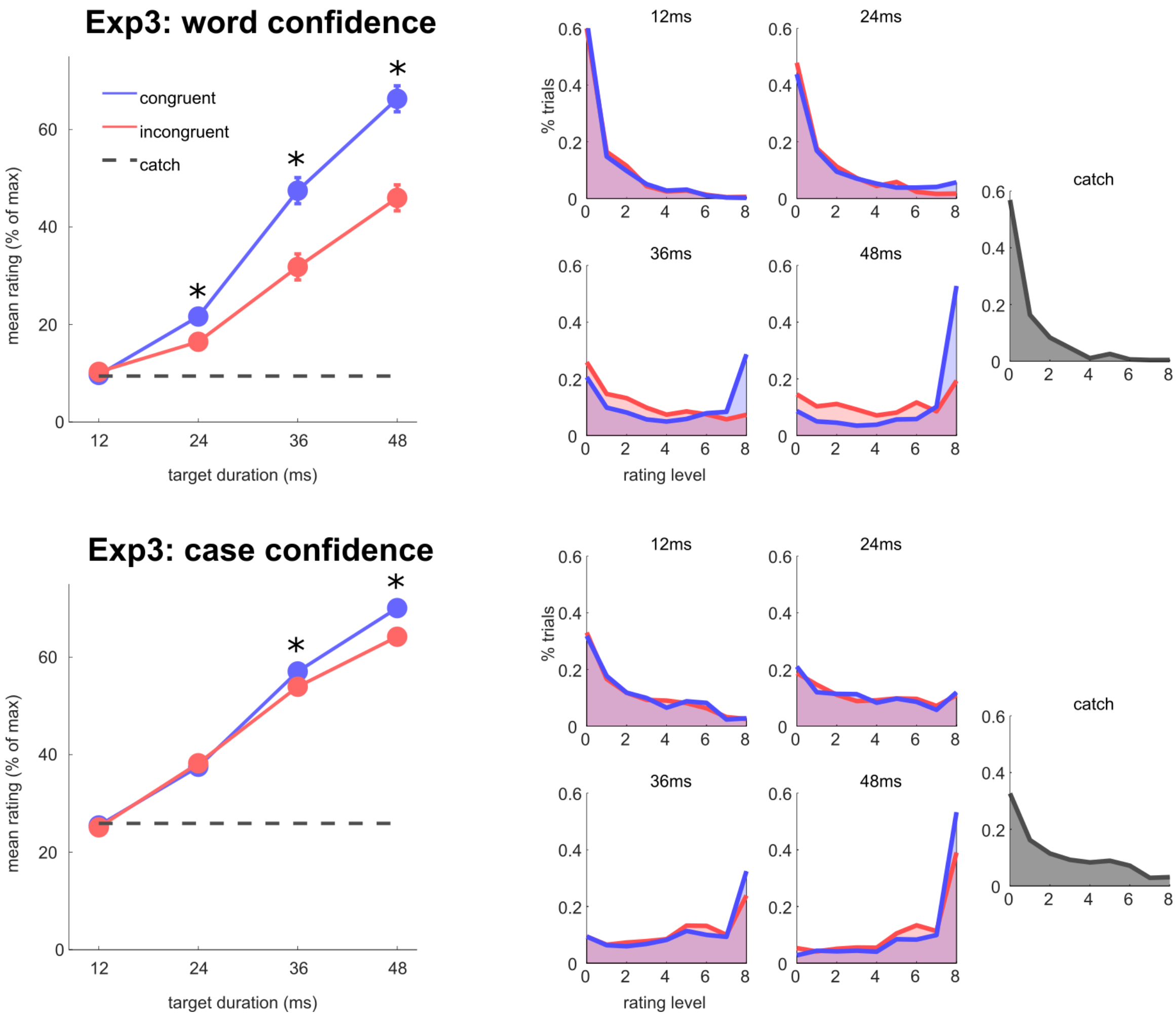

Supplementary Figure 5: Mean confidence ratings (Experiment 3) and confidence distributions, separately for word identification and case discrimination. In Experiment 3, participants were requested to rate their confidence on their word identification response (upper panel), and their case discrimination response (lower panel). Each dot represents the average across participants of the mean confidence rating obtained on the scale for catch (grey), congruent trials (blue) or incongruent trials (red) as a function of target duration. The error bars represent the standard error of the mean difference between congruent and incongruent trials. A repeated measures two-way ANOVA on confidence ratings for word identification revealed a significant increase in confidence with target duration ($F(1.24,21.16)=132.037$, $p<0.001$, $\eta^2G = 0.63$, 95%CI for η^2G = [0.44 0.72]) and congruence ($F(1.00,17.00)=65.538$, $p<0.001$, $\eta^2G = 0.12$, 95%CI for η^2G = [0.00 0.38]), with an interaction between these two factors ($F(2.12,36.12)=28.223$, $p<0.001$, $\eta^2G = 0.08$, 95%CI for η^2G = [0.00 0.21]). A repeated measures two-way ANOVA on confidence ratings for case discrimination revealed a significant increase in confidence with target duration ($F(1.31,22.31)=69.95$, $p<0.001$, $\eta^2G = 0.47$, 95%CI for η^2G = [0.24 0.59]) and congruence ($F(1.00,17.00)=9.07$, $p<0.01$, $\eta^2G = 0.004$, 95%CI for η^2G = [0.00 0.06]), with an interaction between these two factors

($F(1.31,22.31)=7.05$, $p<0.01$, $\eta^2G = 0.006$, 95%CI for η^2G = [0.00 0.02]). Distributions of visibility ratings are shown on the right. Stars represent significant post-hoc comparisons at each target duration (see **Supplementary Note 2**).

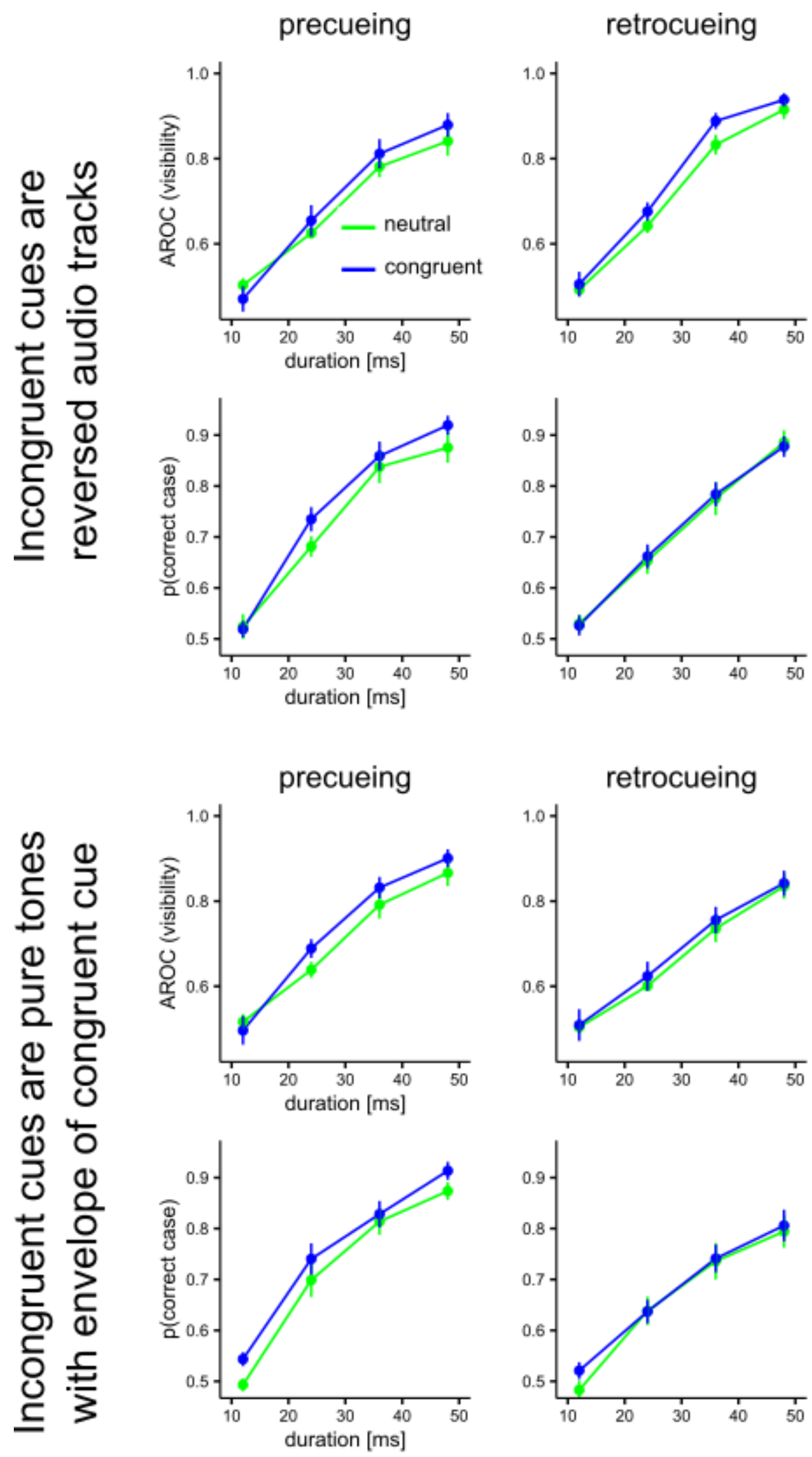

Supplementary Figure 6: Results for Experiments 4 (precue, reversed track), 5 (retrocue, reversed track), 6 (precue, pure tones), and 7 (retrocue, pure tones) shown as in Figure 5 (where data is pooled 4&6/5&7). Legend is as in Figure 5.

Congruent cue	Incongruent cue	Target	% assoc.
RUCHE (hive)	LUGE (sled)	Abeille (bee)	82
PRESENCE	LEVRE (lip)	Absence	43
SOUSTRACTION (subtraction)	TELEPHONE	Addition	54
GIGOT (leg)	GRAVE (low pitch)	Agneau (lamb)	81
SOUTENIR (support)	SALUER (greet)	Aider (help)	29
FAUCON (falcon)	CONFITURE (jam)	Aigle (eagle)	49
GRAVE (low-pitch)	GIGOT (leg)	Aigu (high pitch)	31
PREFERER (prefer)	TERMINER (end)	Aimer (love)	38
BIERE (beer)	PAGE	Alcool (alcohol)	25
COMPAGNON (companion)	CONNAITRE (know)	Ami (friend)	51
AVAL (downstream)	DESSINER (draw)	Amont (upstream)	75
HAINE (hate)	BANQUE (bank)	Amour (love)	38
VEGETAL	GAUCHE (left)	Animal	29
NOMMER (name)	POUSSER (push)	Appeler (call)	32
AMENER (bring by leading)	VITE (fast)	Apporter (bring by carrying)	28
BRANCHE (branch)	MAUVAIS (bad)	Arbre (tree)	85
FLECHE (arrow)	FREQUEMMENT (frequent)	Arc (bow)	74
BANQUE (bank)	HAINE (hatred)	Argent (money)	57
PISTOLET (pistol)	PLANCTON (plankton)	Arme (weapon)	25
OEUVRE (work)	ATTENTION	Art	49

Supplementary Table 1: Examples of stimuli. *Here is a short table showing the first 20 stimulus-pairs from our bank by alphabetical (target) order, with their English translation. The full document is available below (**Supplementary Table 2**). The two first columns correspond to possible congruent and incongruent cues, for the target shown in the third. The last column displays the percentage of association estimated between the target and the corresponding congruent cue as estimates through the procedure described in **Supplementary Note 6**. The incongruent pairs are constituted of cue switches (see example highlighted in green).*

congruent cue	incongruent cue	target	% association
RUCHE	LUGE	abeille	82
PRESENCE	LEVRE	absence	43
SOUSTRACTION	TELEPHONE	addition	54
GIGOT	GRAVE	agneau	81
SOUTENIR	SALUER	aider	29
FAUCON	CONFITURE	aigle	49
GRAVE	GIGOT	aigu	31
PREFERER	TERMINER	aimer	38
BIERE	PAGE	alcool	25
COMPAGNON	CONNAITRE	ami	51
AVAL	DESSINER	amont	75
HAINE	BANQUE	amour	38
VEGETAL	GAUCHE	animal	29
NOMMER	POUSSER	appeler	32
AMENER	VITE	apporter	28
BRANCHE	MAUVAIS	arbre	85
FLECHE	FREQUEMMENT	arc	74
BANQUE	HAINE	argent	57
PISTOLET	PLANCTON	arme	25
OEUVRE	ATTENTION	art	49
SUFFIRE	TRADITION	assez	36
DEBOUT	REGARD	assis	76
PATIENTER	NAVETTE	attendre	61
EGALEMENT	ESSAYER	aussi	50
ARRIERE	ACHETER	avant	40
ATTERRIR	AVRIL	avion	58
OBTENIR	OBSCUR	avoir	48
GIFLE	GAZON	baffe	36
SERVIETTE	CAMERA	bain	29
PLANCTON	PISTOLET	baleine	28
JONGLER	APPUYER	balle	60
FOOTBALL	BANANE	ballon	40
RASER	NAGER	barbe	54
VOILE	TIMBRE	bateau	51
JOLI	OBSERVER	beau	59
ABONDANT	EGYPTE	beaucoup	37
ETRANGE	COFFRE	bizarre	69
COULEUR	MOUFLE	bleu	26
SCIER	SEMER	bois	58
ATOMIQUE	AVEC	bombe	60
MAUVAIS	BRANCHE	bon	42
MALHEUR	MASSE	bonheur	43

SALUER	SOUTENIR	bonjour	52
CHAMEAU	CEINTURE	bosse	29
LEVRE	PRESENCE	bouche	58
APPUYER	JONGLER	bouton	66
BLOND	BOUILLIR	brun	39
OBJECTIF	NABOT	but	39
TRANQUILLE	POIGNEE	calme	30
RURAL	BOUTIQUE	campagne	34
TUMEUR	TOT	cancer	54
BOULET	MIDI	canon	37
PLAN	GRUE	carte	30
BRISER	AIGUILLE	casser	59
DIZAINE	CRACHER	centaine	27
TABLE	VACHE	chaise	44
HASARD	ARTICLE	chance	32
REMPLACER	SIMPLE	changer	32
CHANTER	ARRETER	chanson	34
CASQUETTE	CANNE	chapeau	34
MIAULER	MAJEUR	chat	86
FROID	ROI	chaud	47
PANTOUFLE	PINCEAU	chausson	38
PATRON	SINUS	chef	25
REPASSER	PEINDRE	chemise	26
COUTER	CHEMIN	cher	35
ETALON	ETALON	cheval	71
PEIGNE	FEROCE	cheveux	48
ABOYER	LIQUIDE	chien	78
NOMBRE	AME	chiffre	49
CACAO	CHOUCROUTE	chocolat	61
DECIDER	CONSTRUIRE	choisir	29
BAS/DECISION	LIMACE	haut/choix	68/31
HIBOU	CAUSE	chouette	60
NUAGE	BLOUSON	ciel	38
QUATRE	PIEU	cinq	51
TRAPEZE	ARROSER	cirque	49
PRESSER	OBTENIR	citron	47
OBSCUR	GIRAFE	clair	34
SERRURE	CHAMBRE	clef	71
LUTTE	EXCUSE	combat	34
ABSTRAIT	DEMANGER	concret	74
RECIF	COUCHAGE	corail	51
CRAVATE	SERRURE	costume	30
GIRAFE	CONTRE	cou	61

CORRIDOR	TONNEAU	couloir	75
CISEAUX	PISTE	couper	73
BRAVOURE	PENURIE	courage	52
LONG	LONGITUDE	court	41
SEMOULE	RAQUETTE	couscous	41
FOURCHETTE	SUFFIRE	couteau	49
TRADITION	HAMMAM	coutume	25
GRENOUILLE	AVAL	crapaud	25
DESSINER	MOITIE	crayon	28
CASSEROLE	VENIN	cuisine	32
CRU	OEUVRE	cuit	57
ATTENTION	SOYEUX	danger	41
TANGO	PNEU	danse	68
AUJOURDHUI	CASSEROLE	demain	53
MOITIE	CAPTIF	demi	37
CARIE	DIESEL	dent	67
DESSUS	ONCLE	dessous	82
DEMON	PROMETTRE	diable	39
REDUIRE	DEMEURER	diminuer	35
MEDECIN	NOURRIR	docteur	33
PRETER	TENDANCE	donner	31
SOMMEIL	INDIQUER	dormir	36
SOUFFRANCE	TANGO	douleur	32
SOYEUX	ACTIF	doux	63
ADDICTION	VEGETAL	drogue	60
GAUCHE	FAUX	droite	72
ARDU	BLOND	dur	35
BOUILLIR	BROUETTE	eau	46
GOMME	NUAGE	effacer	32
CAUSE	CHEVRE	effet	33
FAMILLE	PIECE	enfant	25
RETIRER	ADORABLE	enlever	48
ADVERSAIRE	IMPORTER	ennemi	39
ECOUTER	MIEUX	entendre	33
DESIR	HIBOU	envie	25
LIMACE	FRANCAIS	escargot	53
ASTRONAUTE	RATEAU	espace	48
LUEUR	NOMBRE	espoir	39
AME	DESSUS	esprit	39
DIESEL	INSPECTEUR	essence	54
DIGESTION	POULPE	estomac	25
INCLUS	LIEVRE	exclu	35
INSPIRER	ECOUTER	expirer	42

IMPORTER	REMPLACER	exporter	37
SIMPLE	SOUCOUPE	facile	38
SOIF	ANCIEN	faim	39
ERREUR	CHENILLE	faute	41
CANAPE	SECONDE	fauteuil	29
HOMME	ABDOMEN	femme	84
ACIER	ARAIGNEE	fer	49
ALLUMETTE	BRISER	feu	66
AIGUILLE	NATION	fil	38
GARCON	SERVIETTE	fille	74
CAMERA	DIRE	film	34
DEBUT	PREFERER	fin	77
TERMINER	COQ	finir	57
VASE	TRIOMPHE	fleur	63
RIVIERE	CLASSIQUE	fleuve	34
PUISSANCE	ETRANGE	force	35
COFFRE	AS	fort	42
ASILE	TIPI	fou	66
TONNERRE	CREATURE	foudre	28
MICROONDES	FRONCER	four	33
CIGALE	FAUCON	fourmi	53
CONFITURE	FAMILLE	fraise	44
CHEVRE	FOOTBALL	fromage	36
BANANE	ENDROIT	fruit	39
ECHAPPER	RAT	fuir	31
AVENIR	COULEUR	futur	60
MOUFLE	FIGURE	gant	44
MAINTENIR	SOVIETIQUE	garder	26
ANTILOPE	PLEIN	gazelle	34
TYPE	MOUSTIQUE	genre	29
MECHANT	PIGEON	gentil	45
PATIN	SCIER	glace	68
SEMER	PROBLEME	graine	56
IMMENSE	RECIF	grand	37
DEMANGER	EMPORTER	gratter	72
ENORME	SABLE	gros	44
CLAN	RAYON	groupe	30
GAZON	GIFLE	herbe	42
MONTRE	GRIPPE	heure	57
CONTENT	NECESSAIRE	heureux	47
RACONTER	AVANCER	histoire	39
ETE	CHOSE	hiver	45
VINAIGRE	VIRGULE	huile	29

CRIER	CHERCHER	hurler	53
AILLEURS	CONFIER	ici	26
ETAGE	FORCER	immeuble	24
TIPI	TONNERRE	indien	62
CAFARD	BIDET	insecte	30
BRAS	ROCHER	jambe	44
BROUETTE	GOMME	jardin	30
MERCREDI	CORRIDA	jeudi	59
ARTICLE	HASARD	journal	46
TRIBUNAL	TRINGLE	juge	26
ROBE	REMARQUER	jupe	28
PROMETTRE	REDUIRE	jurer	25
CANOE	CANTONNAIS	kayak	74
ETANG	PROTON	lac	38
MOUTON	HERISSON	laine	30
VACHE	TABLE	lait	45
HALOGENE	EPOUX	lampe	41
FRANCAIS	ASTRONAUTE	langue	34
LIEVRE	INSPIRER	lapin	70
LONGITUDE	SEMOULE	latitude	85
BIDET	CAFARD	lavabo	40
RATON	INCLINER	laveur	92
TORTUE	OURS	lent	32
ATTACHER	ALLER	lier	35
ENDROIT	ECHAPPER	lieu	66
PINCE	NORD	linge	38
FEROCE	PEIGNE	lion	28
ECRIRE	VERITE	lire	27
CHAMBRE	LUTTE	lit	39
PAGE	BIERE	livre	58
DISTANCE	EXIGER	loin	30
LEGER	JETER	lourd	42
MONSIEUR	EST	madame	74
BOUTIQUE	RURAL	magasin	34
AVRIL	ATTERRIR	mai	58
DOIGT	ENTREE	main	62
CONSTRUIRE	DECIDER	maison	30
BIEN	CREUX	mal	63
GRIPPE	MONTRE	maladie	31
PAPA	HABITER	maman	72
NOURRIR	PRETER	manger	49
PENURIE	LONG	manque	34
COURIR	DESCENDRE	marcher	27

EPOUX	HALOGENE	mari	41
FIANCAILLES	ESTIME	mariage	51
CLOU	INSTANT	marteau	43
REVEIL	LAITUE	matin	55
MIEUX	DESIR	meilleur	34
VERITE	ECRIRE	mensonge	36
VAGUE	RICHE	mer	54
PLACER	REFLECHIR	mettre	25
BACTERIE	AUTOMATE	microbe	26
ADORABLE	ADVERSAIRE	mignon	47
CENTRE	MOT	milieu	43
MAJEUR	MIAULER	mineur	45
MAXIMUM	WAGON	minimum	79
MIDI	BOULET	minuit	36
SECONDE	HOMME	minute	43
TENDANCE	SOMMEIL	mode	40
INSTANT	CLOU	moment	58
PIECE	RETIRER	monnaie	37
CREATURE	MICROONDES	monstre	27
COLLINE	EXISTENCE	montagne	43
DESCENDRE	COURIR	monter	51
INDIQUER	SOUFFRANCE	montrer	49
DEUIL	SUJET	mort	61
SCOOTER	SEPTEMBRE	moto	50
CLOISON	CERCLE	mur	56
CLASSIQUE	PUISSANCE	musique	52
NABOT	OBJECTIF	nain	60
LUGE	RUCHE	neige	74
PROTON	ETANG	neutron	49
SINUS	PATRON	nez	53
BLANC	DERNIER	noir	62
COCO	MOISI	noix	56
OUI	OTARIE	non	81
JOUR	TOUPIE	nuit	69
FORCER	ETAGE	obliger	27
SEPTEMBRE	SCOOTER	octobre	44
PARFUM	VALENTIN	odeur	56
ECHALOTTE	QUI	oignon	47
PIGEON	PATIN	oiseau	29
VERNIS	VEINE	ongle	55
AVIS	MOULIN	opinion	46
FIBRE	TRANSPIRER	optique	36
ECLAIR	ECLAIR	orage	29

TYMPAN	DEROBER	oreille	77
COUSSIN	PROCES	oreiller	28
OCCIDENT	UNIQUE	orient	61
SQUELETTE	EMPESTER	os	71
EST	MONSIEUR	ouest	39
BAGUETTE	BONDIR	pain	68
GUERRE	HARICOT	paix	40
OSIER	ORTEIL	panier	68
CEINTURE	CHAMEAU	pantalon	35
FEUILLE	PARQUET	papier	32
CHENILLE	CANAPE	papillon	49
ENFER	HUMOUR	paradis	30
EXCUSE	ABSTRAIT	pardon	36
SEMBLABLE	PARAITRE	pareil	38
MISER	QUITTER	parier	38
DIRE	DEBUT	parler	50
QUITTER	MISER	partir	49
PIETON	ARCHET	passage	34
ACTIF	ADDICTION	passif	47
RICHE	VAGUE	pauvre	53
NATION	GARCON	pays	44
PINCEAU	PANTOUFLE	peinture	39
RATEAU	LUEUR	pelle	39
OURS	TORTUE	peluche	26
INCLINER	RATON	pencher	49
REFLECHIR	PLACER	penser	68
GAGNER	DOUBLE	perdre	30
COLLIER	BUREAU	perle	42
CRAINTE	PROPRE	peur	81
OTARIE	OUI	phoque	34
ALBUM	ECROU	photo	92
MOT	CENTRE	phrase	34
CHIMIE	BANQUISE	physique	37
ORTEIL	OSIER	pied	67
ROCHER	BRAS	pierre	27
POULPE	INCLUS	pieuvre	40
FACE	PROCHAIN	pile	36
BANQUISE	CHIMIE	pingouin	28
HERISSON	MOUTON	piquant	34
AS	ASILE	pique	37
MOUSTIQUE	MECHANT	piqure	25
NAGER	RASER	piscine	52
SABLE	CLAN	plage	38

ARROSER	PRESSER	plante	34
STYLO	GORILLE	plume	29
MASSE	MALHEUR	poids	45
VIRGULE	VINAIGRE	point	75
ECAILLE	ERUPTION	poisson	64
INSPECTEUR	DIGESTION	police	26
POIRE	POIVRE	pomme	63
TELEPHONE	SOUSTRACTION	portable	36
POIGNEE	TRANQUILLE	porte	45
SOULEVER	FRERE	porter	31
TIMBRE	VOILE	poste	61
JETER	LEGER	poubelle	27
COQ	VASE	poule	56
CONTRE	CORRIDOR	pour	60
MOISI	COCO	pourri	39
DERNIER	BLANC	premier	52
EMPORTER	ENORME	prendre	28
CAPTIF	CARIE	prison	36
DICTON	POUMON	proverbe	25
EMPESTER	SQUELETTE	puer	71
EGYPTE	ABONDANT	pyramide	33
QUI	ECHALOTTE	quoi	60
PEPIN	ROMARIN	raisin	30
VITE	AMENER	rapide	47
ZEBRE	BRUIT	rayure	43
AVANCER	RACONTER	reculer	46
OBSERVER	JOLI	regarder	43
ROI	FROID	reine	60
REGRET	MONDE	remord	31
RUSE	SAIGNANT	renard	47
POUMON	DICTON	respirer	26
DEMEURER	MEDECIN	rester	50
TRINGLE	TRIBUNAL	rideau	49
TOUT	TOLE	rien	63
HUMOUR	ENFER	rire	29
CANTONNAIS	CANOE	riz	86
AUTOMATE	BACTERIE	robot	58
CERCLE	CLOISON	rond	54
TOMATE	VALISE	rouge	49
CHEMIN	COUTER	route	32
SOVIETIQUE	ANTILOPE	russe	31
COUCHAGE	CRAVATE	sac	53
VALENTIN	PARFUM	saint	54

LAITUE	REVEIL	salade	70
PROPRE	CRAINTE	sale	50
CRACHER	DIZAINE	salive	31
VEINE	VERNIS	sang	38
AVEC	ATOMIQUE	sans	67
CHOUCROUTE	CACAO	saucisse	29
HAMMAM	GRENOUILLE	sauna	35
BONDIR	BAGUETTE	sauter	52
SECOURIR	PLOMBIER	sauver	33
CONNAITRE	COMPAGNON	savoir	64
CONFIER	AILLEURS	secret	37
POITRINE	ENTRAINEMENT	sein	33
POIVRE	POIRE	sel	90
PARAITRE	SEMBLABLE	sembler	34
DIRECTION	GLISSER	sens	29
VENIN	CRU	serpent	77
UNIQUE	OCCIDENT	seul	46
GORILLE	STYLO	singe	34
ERABLE	ELEPHANT	sirop	55
PISTE	BRAVOURE	ski	38
FRERE	SOULEVER	sœur	63
ESTIME	FIANCAILLES	soi	40
PARQUET	FEUILLE	sol	47
RAYON	DECISION	soleil	42
PROBLEME	IMMENSE	solution	47
BRUIT	ZEBRE	son	29
ENTREE	DOIGT	sortie	65
FRONCER	CIGALE	sourcil	51
RAT	AVENIR	souris	39
FREQUEMMENT	FLECHE	souvent	80
NAVETTE	PATIENTER	spatiale	32
ENTRAINEMENT	POITRINE	sport	32
SAIGNANT	RUSE	steak	34
ARRETER	CHANTER	stop	28
CANNE	CASQUETTE	sucre	71
NORD	PINCE	sud	63
TRANSPIRER	FIBRE	suer	36
PROCHAIN	FACE	suivant	49
PEINDRE	REPASSER	tableau	29
ONCLE	DEMON	tante	74
TOT	TUMEUR	tard	67
CORRIDA	MERCREDI	taureau	64
RAQUETTE	FOURCHETTE	tennis	89

ESSAYER	EGALEMENT	tenter	41
MONDE	REGRET	terre	35
ROMARIN	PEPIN	thym	37
POUSSER	NOMMER	tirer	25
ARAIGNEE	ALLUMETTE	toile	40
TOLE	TOUT	toit	29
GLISSER	DIRECTION	tomber	29
TOUPIE	JOUR	tourner	44
WAGON	MAXIMUM	train	86
BUREAU	COLLIER	travail	47
GRUE	PLAN	travaux	36
DOUBLE	GAGNER	triple	26
ELEPHANT	ERABLE	trompe	39
CREUX	BIEN	trou	29
CHERCHER	CRIER	trouver	65
CHOSE	ETE	truc	42
PLOMBIER	SECOURIR	tuyau	26
NECESSAIRE	CONTENT	utile	28
PIEU	TRAPEZE	vampire	37
ACHETER	ARRIERE	vendre	31
ALLER	ATTACHER	venir	28
MOULIN	AVIS	vent	42
ABDOMEN	ACIER	ventre	50
PROCES	COUSSIN	verbal	26
SUJET	DEUIL	verbe	39
LIQUIDE	ABOYER	verser	31
HARICOT	GUERRE	vert	43
BLOUSON	QUATRE	veste	40
TRIOMPHE	RIVIERE	victoire	35
PLEIN	TYPE	vide	52
EXISTENCE	COLLINE	vie	57
ANCIEN	ERREUR	vieux	50
TONNEAU	CISEAUX	vin	73
ARCHET	PIETON	violon	36
ECROU	ALBUM	vis	44
FIGURE	MAINTENIR	visage	39
HABITER	PAPA	vivre	41
REMARQUER	ROBE	voir	44
PNEU	AUJOURDHUI	voiture	48
SOUCOUPE	SOIF	volante	74
ERUPTION	ECAILLE	volcan	68
DEROBER	TYMPAN	voler	79
EXIGER	DISTANCE	vouloir	27

VALISE	TOMATE	voyage	44
FAUX	ARDU	vrai	59
REGARD	DEBOUT	yeux	47

Supplementary Table 2: Full stimulus list.

Supplementary Table 3: Model fit diagnostics (main effects).

	Target duration	congruency	Interaction dur x congr
**Exp 1**			
R-hat identification	1.003	1.002	1.001
n_eff identification	1150	3191	2834
R-hat discrimination	1.003	1.002	1.002
n_eff discrimination	1091	2413	2132
**Exp 2**			
R-hat identification	1.003	1.000	1.001
n_eff identification	1031	2552	2457
R-hat discrimination	1.000	1.000	1.000
n_eff discrimination	1718	2735	2572
**Exp 3**			
R-hat identification	1.009	1.001	1.002
n_eff identification	725	2319	1893
Rhat discrimination	1.002	0.999	1.000
n_eff discrimination	1683	3562	3431

**Exps 4-7 full model**	Target duration	congruency	Cue type	Dur x congr	Congr x cue	Dur x cue	triple
R-hat discrimination	1.001	1.004	1.002	1.003	1.003	1.001	1.004
n_eff discrimination	1326	1832	1300	2073	1943	1208	2042

Indications of a good quality of fit are: Rhat values nearing 1.0 indicate that independent parameter estimates have converged to the same value. Rhat < 1.01 are usually considered sufficient for appropriate convergence[4]. Effective sample size (n_eff) above 1000 indicate stable estimates of parameter values[5].

SI References

1. Debrenne, M. Le dictionnaire des associations verbales du français et ses applications. *Variétés, variations and formes du français. Palaiseau: Éditions de l'Ecole polytechnique* 355–66 (2011).
2. Ferrand, L. & Alario, F.-X. Normes d'associations verbales pour 366 noms d'objets concrets. *L'Année psychologique* **98**, 659–709 (1998).
3. Ferrand, L. Normes d'associations verbales pour 260 mots «abstraits». *L'Année psychologique* **101**, 683–721 (2001).
4. Vehtari, A., Gelman, A., Simpson, D., Carpenter, B. & Bürkner, P.-C. Rank-Normalization, Folding, and Localization: An Improved Rˆ for Assessing Convergence of MCMC (with Discussion). *Bayesian Analysis* **16**, 667–718 (2021).
5. Bürkner, P.-C. brms: An R package for Bayesian multilevel models using Stan. *Journal of statistical software* **80**, 1–28 (2017).
